# Proteome of fraction from *Tityus serrulatus* venom reveals new enzymes and toxins

**DOI:** 10.1590/1678-9199-JVATITD-1482-18

**Published:** 2019-04-18

**Authors:** Fernanda Gobbi Amorim, Heloisa Tavoni Longhim, Camila Takeno Cologna, Michel Degueldre, Edwin De Pauw, Loïc Quinton, Eliane Candiani Arantes

**Affiliations:** 1 Department of Physics and Chemistry, School of Pharmaceutical Sciences of Ribeirão Preto, University of São Paulo, Av. do Café s/n, Monte Alegre, Ribeirão Preto, SP 14040-903, Brazil.; 2 University of Vila Velha, Vila Velha, ES, Brazil.; 3 Laboratory of mass spectrometry, MolSys Research Unit, Liège Université, Liège, Belgium.

**Keywords:** Tityus serrulatus, scorpion venom, enzymes, proteases, ACE inhibitors, proteome

## Abstract

**Background:**

*Tityus serrulatus* venom (*Ts* venom) is a complex mixture of several compounds with biotechnological and therapeutical potentials, which highlights the importance of the identification and characterization of these components. Although a considerable number of studies have been dedicated to the characterization of this complex cocktail, there is still a limitation of knowledge concerning its venom composition. Most of *Ts* venom studies aim to isolate and characterize their neurotoxins, which are small, basic proteins and are eluted with high buffer concentrations on cation exchange chromatography. The first and largest fraction from carboxymethyl cellulose-52 (CMC-52) chromatography of *Ts* venom, named fraction I (Fr I), is a mixture of proteins of high and low molecular masses, which do not interact with the cation exchange resin, being therefore a probable source of components still unknown of this venom. Thus, the present study aimed to perform the proteome study of Fraction I from *Ts* venom, by high resolution mass spectrometry, and its biochemical characterization, by the determination of several enzymatic activities.

**Methods:**

Fraction I was obtained by a cation exchange chromatography using 50 mg of crude venom. This fraction was subjected to a biochemical characterization, including determination of L-amino acid oxidase, phospholipase, hyaluronidase, proteases activities and inhibition of angiotensin converting enzyme (ACE) activity. Fraction I was submitted to reduction, alkylation and digestion processes, and the tryptic digested peptides obtained were analyzed in a Q-Exactive Orbitrap mass spectrometer. Data analysis was performed by PEAKS 8.5 software against NCBI database.

**Results:**

Fraction I exhibits proteolytic activity and it was able to inhibit ACE activity. Its proteome analysis identified 8 different classes of venom components, among them: neurotoxins (48%), metalloproteinases (21%), hypotensive peptides (11%), cysteine-rich venom protein (9%), antimicrobial peptides (AMP), phospholipases and other enzymes (chymotrypsin and lysozymes) (3%) and phosphodiesterases (2%).

**Conclusions:**

The combination of a proteomic and biochemical characterization strategies leads us to identify new components in the *T. serrulatus* scorpion venom. The proteome of venom´s fraction can provide valuable direction in the obtainment of components in their native forms in order to perform a preliminary characterization and, consequently, to promote advances in biological discoveries in toxinology.

## Background

Scorpion venoms are a rich source of components with diverse biological activities and high specificity for their targets [1]. Their venoms are usually composed of insoluble mucus, oligopeptides, mucopolysaccharides, nucleotides, low molecular weight molecules (serotonin or histamine), protease inhibitors, histamine releasers, amino acids and other organic compounds, enzymes and many neurotoxins [2-4]. Among this impressive cocktail, neurotoxins are the main components studied, mostly because of their interaction with Na^+^ or K^+^ channels and their importance in the scorpion envenoming [5]. In addition, several enzymes have been characterized in scorpion venoms, including phospholipase A, hyaluronidase, sphingomyelinase D, lysozyme, metalloproteases and serine proteases. Other enzymes have been related with the post-translational processing of toxin precursors and with the facilitation of venom permeation into tissues [3, 6].

The study of venom components is highly useful for elucidating the biochemical process of envenoming, but also for identifying molecules that can be used as molecular tools and/or drugs with therapeutic action. So far, the number of distinct toxins present in scorpion venoms has been estimated at about 100.000, and only less than 1% has been characterized [7, 8].

The initial fractionation of *Tityus serrulatus (Ts)* venom on a carboxymethyl cellulose-52 (CMC-52) column, described by Arantes et al. [9] and modified by Cerni et al. [5], uses ammonium bicarbonate buffer, pH 7.8, and allows separate *Ts* venom components in 18 fractions, according to their charge. The positively charged proteins at this pH (such as neurotoxins) interact with the cation exchange resin and the other proteins are eluted in the first fractions of the chromatography. Fraction I (Fr I) is the first and largest peak eluted on CMC-52 chromatography and was never in depth studied before. It is composed by a mixture of proteins of high and low molecular masses, being a probable source of components still unknown of *T. serrulatus* venom and, therefore, was chosen to be analyzed in this study [9].

Proteomic techniques have been employed to explore the diversity of toxins present in scorpion venom. Protein separation techniques, such as high performance liquid chromatography (HPLC) hyphened with soft ionization mass spectrometers, allowed a more detailed proteomic analysis of animal venoms [1, 10-18]. Those techniques provide a broad range of structural information such as the amino acid sequence for peptides, accurate determination of molecular mass, determination of disulfide bonds, and characterization of post-translational modifications [19]. Furthermore coupled techniques such as chromatography and electrospray ionization (ESI) permit mass spectrometry to become the method of choice for the analysis of complex mixtures such as animal venoms [20].

With the advent of omics techniques, studies of animal venoms have become increasingly faster and more comprehensive, often able to provide a holistic view of all components in the venom specially when combined to Next Generation Sequencing (NGS) transcriptomics [21]. Therefore, due the high complexity of scorpion venoms, a proteome fraction-directed or fraction subproteomes combined to shotgun proteomics can be useful in the identification of new molecules and to guide the obtainment of components in their native forms in order to perform a preliminary characterization. Considering this panorama, the present study carried out a high resolution proteome of Fraction I, the first fraction of the cation exchange chromatography from *Tityus serrulatus* venom, and its biochemical characterization, aiming the exploration and bioprospection of undiscovered components.

## Methods

### Venom Fractionation

*Ts* venom was obtained from the vivarium of the Faculdade de Medicina de Ribeirão Preto da Universidade de São Paulo (School of Medicine of Ribeirão Preto, University of São Paulo, Brazil), using electrical stimulation of 12 mV. The freeze-dried venom was diluted in ultra pure water (MilliQ^®^) and desiccated for 6 h. After this procedure, the sample (50 mg) was resuspended in 500 μL of 50 mM ammonium bicarbonate buffer, pH 7.8, and centrifuged at 13,000 rpm for 10 min at 4 °C (Centrifuge 5415 R). Ammonium bicarbonate buffer (500 μL) was added to the precipitate, the mixture was homogenized and centrifuged. This process was repeated twice. The supernatants from the 4 extractions (final volume of 2.0 mL) were pooled, held at 4 °C for 12 hours, and centrifuged at 13,000 rpm for 10 minutes. At the end of this process, the soluble venom (without mucus) was submitted to a fast protein liquid chromatography (FPLC), using a CMC-52 microgranular column 1.6 x 100 cm (Whatman, UK), equilibrated with buffer A (50 mM ammonium bicarbonate, pH 7.8), as described by Cerni et al. [5]. The sample (2 mL) was initially eluted with buffer A, followed by a linear concentration gradient (0 to 100%) of buffer B (0.6 M ammonium bicarbonate, pH 7.8), under a flow rate of 0.5 mL/min and temperature of 25 °C. Absorbance was automatically recorded at 280 nm. All the fractions obtained were lyophilized and stored at −20◦C.

### Eletrophoresis

The fractions obtained from the venom chromatography were analyzed by tricine sodium dodecyl sulfate polyacrylamide gel electrophoresis (Tricine-SDS-PAGE) following the method used for ultra-low mass proteins [22]. It was used a 16.5% separating gel, overlaid by a 5% stacking gel. The gels were stained with PlusOne Coomassie Blue PhastGel^®^ R-350 (GE Healthcare, UK) and destained with 10% acetic acid (v/v). The ultra-low range molecular weight marker (Sigma-Aldrich Co., USA) was used.

### Biochemical characterization

### Phospholipase activity

Phospholipase activity was assessed in Petri dishes [23], with the following modifications: agarose was replaced by agar and erythrocytes were not used. Briefly, a gel containing 0.01 M CaCl_2_, egg yolk diluted in phosphate-buffered saline (PBS) at pH 7.2 in the ratio 1:3 (v/v), 1% bacteriological agar, and 0.005% sodium azide was prepared in Petri dishes. Then, 40 µL of the fraction was applied into 5-mm diameter holes made in the gel, followed by incubation at 37 °C overnight. The formation of translucent halos around the holes in the gel indicated phospholipase activity.

### Proteolytic activity on azocasein

The azocaseinolytic activity was determined by colorimetric assay [24], in which 85 μL of an azocasein solution (5 mg/mL in 50 mM Tris-HCl pH 8.0) were incubated with 10 μL of venom fraction (Fraction I: 47 μg/μL) in 50 mM Tris-HCl, pH 8, and 5 μL of solution of 100 mM protease inhibitors (phenylmethylsulfonyl fluoride, PMSF and ethylenediamine tetraacetic acid, EDTA), for 90 minutes at 37 °C. Next, 200 μL of 5% trichloroacetic acid (TCA) (v/v) was added to the samples followed by centrifugation at 1000 x*g* for 5 minutes. Then, 150 μL of 0.5 M NaOH were added to the supernatants and read at 450 nm.

### L-amino acid (LAAO) activity

LAAO activity was detected spectrophotometrically according to Kishimoto and Takahashi [25]. Each venom fraction was incubated with 2 mM *o*-phenylenediamine (Sigma-Aldrich Co., USA), 1 U/mL horseradish peroxidase (Sigma-Aldrich Co., USA), 5 mM L-leucine (Sigma-Aldrich Co., USA), and 0.05 M Tris-HCl buffer, pH 7.0. Incubation was performed at 37 ° C for 1 hour and then the reaction was quenched with 2 M H_2_SO_4_. The absorbance was determined on a microplate reader at wavelength of 492 nm, with reference to absorbance at 630 nm. To determine the amount of H_2_O_2_ released, a calibration curve was made with H_2_O_2_ (0 - 10 mM). According to Kishimoto and Takahashi [25], 1 unit of LAAO activity is defined as the amount of enzyme required to produce 1 μmol of H_2_O_2_ per minute under the specified conditions.

### Angiotensin Converting Enzyme (ACE) inhibitory activity

ACE inhibitory activity *in vitro* was evaluated following the procedures described by Li et al. (2005) and modified by Pinheiro-Júnior (2018) to miniaturize the methodology to 2 mL micro tubes [26, 27]. The mixture of 30 µL of 5 mM Hippuryl-His-Leu (HHL, Sigma-Aldrich Co., USA) in 100 mM borate buffer, pH 8.3 (Sigma-Aldrich Co., USA), containing 300 mM NaCl, was incubated for 5 min, at 37 ºC, with 20 µL of each venom fraction solution. As negative control of inhibition (100% ACE activity), 20 µL of the same buffer was used. ACE (20 µL of 100 mU/mL solution; Sigma-Aldrich Co., USA) was later added to the mixture, which was then incubated for 2 h, at 37 ºC. The reaction was stopped with addition of 20 µL of HCl 2 N. The hippuric acid (HA), formed during ACE catalysis, was quantified by the incubation with 120 µL of quinoline (Sigma-Aldrich Co., USA) and 40 µL of benzenesulfonyl chloride (BSC, SigmaAldrich Co., USA) for 30 min, at 30 ºC. The chromogen formed by HA-quinoline-BSC was diluted with 250 µL of ethanol, followed by another incubation, under the same conditions. Then, 290 µL of the mixture was transferred to a 96-well plate and the absorbance was measured at 492 nm in a microplate absorbance reader (Sunrise Basic TECAN, Austria). The percentage of ACE inhibitory activity was calculated by the multiplication by 100 of the difference from the absorbance in the reaction without and with inhibitor divided by the difference of absorbance in the reaction without inhibitor and negative control. All the experiments were performed in triplicate.

### Hyaluronidase activity

Hyaluronidase activity was quantitatively determined by the turbidimetric assay as described by Pukrittayakamee et al. [28], adapted to a 96-well microplate and under conditions described by Amorim et al. [29]. For this assay it was used acetate buffer (200 mM sodium acetate and 150 mM NaCl pH 6.0), 10 μg hyaluronan (0.5 mg/mL in water) and the fraction I to the final volume of 200 μL. The mixture was incubated for 30 min at 37 °C, and the reaction was stopped with the addition of 100 μL of 5% cetyltrimethylammonium bromide (CTAB) and 4% NaOH (w/v). Then, the absorbance of the mixture was read at 400 nm in a microplate reader (Sunrise Basic, Tecan, Switzerland). Hyaluronidase activity was expressed as the percentage of hydrolyzed hyaluronan, considering the absorbance of the tube in which no hyaluronan was added as 100% of hydrolysis.

### Inhibition/potentiation of trypsin and chymotrypsin activity

A possible inhibitor or enhancer of trypsin/chymotrypsin activity present in venom fraction was tested. The reaction mixture was prepared as described by the supplier (PBS, enzyme, venom and chromogenic substrate specific for each enzyme). The assay was performed with bovine trypsin and α-chymotrypsin (1 mg/50 mL, 0.001 M HCl; Sigma-Aldrich Co., USA), trypsin and α-chymotrypsin substrates (10 mg/mL N-*p*-Tosyl-Gly-Pro-Lys-4-nitroanilide acetate salt and 0.2 mg/mL N-Succinyl-Ala-Ala-Pro-Phe-*p*-nitroanilide, respectively; Sigma-Aldrich Co., USA), phosphate-buffered saline (PBS) pH 7.4, and Fraction I at two concentrations (1.90 mg/mL and 5.65 mg/mL). The substrate solution was prepared at the 1 mg/20 mL assay concentration in PBS. Volumes of 5 μL of bovine trypsin and α-chymotrypsin were tested with 5 μL of substrate in the 96-well samples at room temperature with 100 μL PBS. The reaction mixtures were read at 410 nm to quantify the formation of p-nitroaniline (yellowish color) every 2 minutes during 200 minutes.

### Statistical Analyses

Experimental data are presented as mean ± SD, and they were analyzed with the GraphPad Prism software, version 6.0 for Windows (GraphPad Software, La Jolla, USA, 2012), using Student’s t-test or ANOVA followed by Sidak *post-hoc*. Values of p < 0.05 were considered statistically signiﬁcant.

### Proteomics analysis

### In solution digestion of Fraction I

The lyophilized Fraction I was dispersed with 200 μL ultrapure water. The sample remained in the water bath at 37 °C for 20 minutes and then centrifuged for 5 minutes. The sample was quantified by RC-DC protein assay (Bio-Rad, USA) and the concentration found was 8 μg/mL. For reduction of the Fraction I, an aliquot of the lyophilized sample was re-suspended in 8 mL of 25 mM NH_4_HCO_3_ and 2 μL of 100mM dithiothreitol and incubated for 1 hour at 56 °C and 300 rpm. For further alkylation, 1.5 μL of 500 mM iodoacetamide was added to the sample and incubated in the dark for 1 hour at room temperature. Then, the fraction sample was digested by trypsin in 50 mM NH_4_HCO_3_, pH 7.8, in a ratio of 1:50 and incubated overnight, at 37 °C, under shaking at 300 rpm. Reaction was stopped by adding 10% TFA (v/v) to the reaction mixture, and the sample was dried on speed vacuum. Sample was suspended in 20 µL of 0.1% TFA (v/v) for desalting on ZipTip™ pipette tips with C18 resin (Millipore, Darmstadt, Germany), using an acetonitrile/water/TFA (49.8/50/0.2 v/v) solution as eluent.

### Shotgun proteomics

For shotgun proteomics analysis, the digested material was analyzed in the Acquity UPLC^®^ M-Class (Waters, Milford, MA, USA) coupled to the Q-Exactive Orbitrap™ Mass Spectrometer (Thermo Scientiﬁc, Bremen, Germany). The chromatographic system is equipped with a 100 μm x 25 cm monolithic PepSwift capillary column (Thermo Scientific, Waltham, MA, USA). The elution of the peptides was performed with a gradient of 3-50% solution B in 80 minutes (A: H2O / 0.1% FA; B: acetonitrile) in a flow rate of 0.7 mL/min.

### Data analysis

Raw data was loaded into Peaks 8.5 software (Bioinformatics solutions, Waterloo, Canada) [30] with database created by the deposits with “Scorpion” keyword from NCBI database downloaded in June 2018 (42,656 sequences). Carbamidomethylation was set as ﬁxed modiﬁcation, while oxidation (M) and amidation were set as variable modiﬁcations. The maximum missed cleavages were set at 3 for trypsin. Parent mass and fragment mass error tolerance were fixed at 5 ppm and 0.015 Da, respectively. False discovery rate (FDR) of 1% and unique peptide ≥2 were used for ﬁltering out inaccurate proteins for the SPIDER search. Only peptides with −10lgP > 20 were used to detect the proteins from the database. The percentage of the venom components in the Fraction I was calculated over the total proteins detected using LC − MS/MS [31].

## Results

### Biochemical characterization

**Figure 1.**
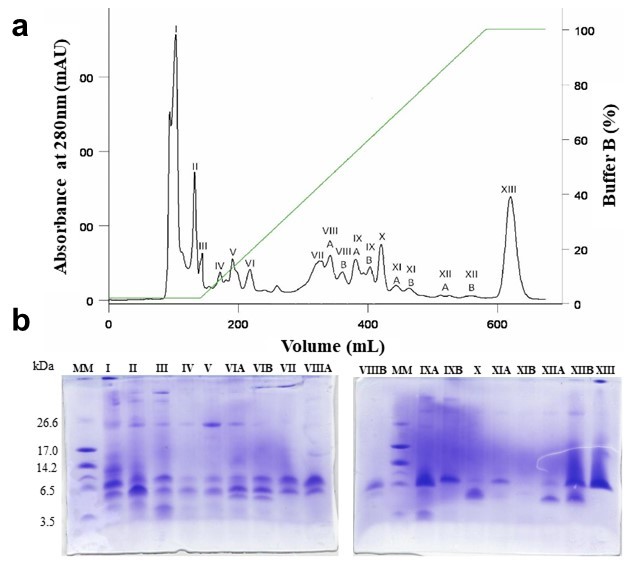
**A**: Chromatographic profile of *Tityus serrulatus* venom in FPLC system with CMC-52 column. Fractionation of *Ts* venom (50 mg) was performed in a FPLC system with CMC-52 column, equilibrated with buffer A (50 mM ammonium bicarbonate, pH 7.8), under a flow rate of 0.5 mL/min and 25 °C. Sample (2 mL) was initially eluted with buffer A, followed by a linear concentration gradient (0 to 100%) of buffer B (0.6 M ammonium bicarbonate, pH 7.8), represented by the green line. Volume collected per tube: 4.0 mL. **B**: Electrophoretic profile of the fractions from the CMC-52 chromatography in 16.5% Tricine-SDS-PAGE. Gel was stained with PlusOne Coomassie Blue PhastGel^®^ R-350 and destained with 10% acetic acid (v/v).

*Ts* venom was fractioned using a cation exchange column CMC-52, resulting in 18 fractions (Fig. 1A). Fraction I is the first to be eluted during the chromatography and represents around 30 % of the soluble venom. According to the electrophoresis gel it is possible to observe that this fraction is rich on a wide range of molecular mass components (Fig. 1B).

**Figure 2.**
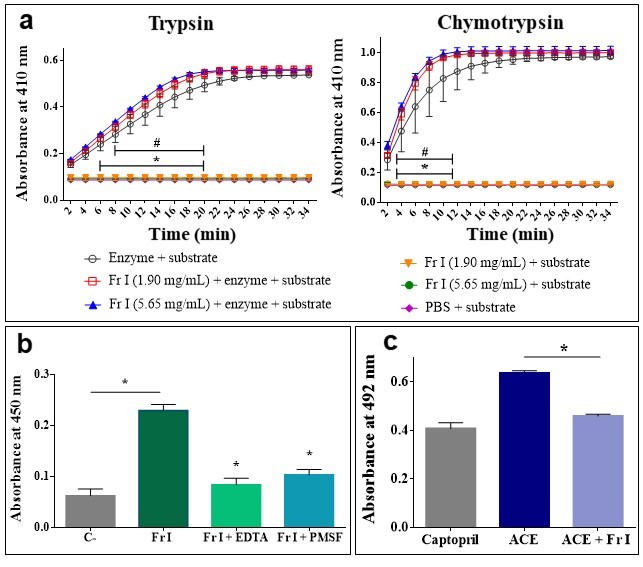
**A**: Trypsin and chymotrypsin activity assay in the presence of fraction I in two concentrations (1.90 mg/mL and 5.65 mg/mL). The absorbance was determined at 410 nm. *p<0.05 Fr I (5.65 mg/mL) + Trypsin + substrate *vs* Trypsin + substrate; #p<0.05 Fr I (1.90 mg/mL) + Trypsin + substrate *vs* Trypsin + substrate. **B**: Proteolytic activity of Fraction I (10 μL, 47 μg/μL) over azocasein in the absence and presence of inhibitors of metalloprotease (EDTA) and serine protease (PMSF). The absorbance was determined at 450 nm. *p<0.05 Fr I + EDTA and Fr I + PMSF *vs* Fr I. **C**: Inhibition assay of ACE activity by Fraction I (48.4 mg/mL). Reactions were read at 492 nm. *p<0.05 ACE + Fr I *vs* ACE. C-: negative control; Fr: Fraction I. Values are expressed as mean ± SD.

Fraction I was subjected to different assays for the identification of proteases (serine proteases, metalloproteinases), L-amino acid oxidases, hyaluronidases and bradykinin potentiating peptides. Fraction I did not demonstrated phospholipase, hyaluronidase or LAAO activities (data not shown). However, it exhibits proteolytic activity, as evidenced by increased proteolysis in trypsin and chymotrypsin assays (Fig. 2A) and by the hydrolysis of azocasein substrate (Fig. 2B). The azocasein assay indicates that metalloproteinases (inhibition by EDTA) and serine proteases (inhibition by PMSF) are present in this fraction. In addition, Fraction I was also able to inhibit 77.4% of the ACE activity (Fig. 2C).

### Proteomics

Mass spectrometry analyses resulted in 4,321 MS and 28,504 MS/MS scans for Fraction I. After applying the parameters described in the Methods section, Fraction I presented 8,017 Peptide Spectrum Matches and 882 peptide sequences that matched the database information. These peptides belong to 66 proteins, among which 56 were identified with more than 2 unique peptides and 10 proteins with 2 unique peptides.

The peptides obtained by high resolution nano-LC-ESI-MS/MS were *de novo* sequenced using the SPIDER algorithm dedicated to the searches into the “Scorpion” database created from NCBI in June 2018. We have considered as an accurate identification just the proteins that matched with at least 2 unique peptides. Therefore, with the *de novo* sequencing, it was obtained 3,269 mass spectra’s, with represented in 95 proteins that matched with the database in the SPIDER section analysis. The full list of proteins and peptides found in this study is reported in Additional file 1.

**Figure 3.**
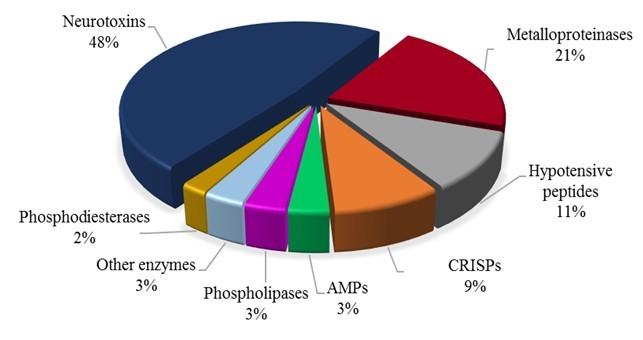
Relative distribution of venom protein classes in Fraction I from *Ts* venom determined by shotgun-proteomics. AMPs: antimicrobial peptides; CRISPs: cysteine-rich secretory proteins; Other enzymes: lysozyme, chymotrypsin and peptidylglycine alpha-amidating monooxygenase.

The results obtained by the biochemical characterization corroborates the proteomics analysis. Among them, 48% were neurotoxins, 21% metalloproteinases, 11% hypotensive peptides and 9% cysteine-rich venom protein (CRISPs). In this analysis, Fraction I also showed to have antimicrobial peptides (AMPs), phospholipases and other enzymes, such as chymotrypsin and lysozymes, which represents 3% each of all venom components identified. In addition, it was identified phosphodiesterases (2%) in this fraction (Fig. 3).

Fraction I presents several components of high molecular mass, such as enzymes and CRISPs, since it is possible to observe in the electrophoresis gel. The proteomic study identified 8 different venom components classes. De Oliveira et al. [32] described an integration of proteome and transcriptome studies of *T. serrulatus* and found 14 different classes of venom components, herein we found that 8 of them exist in Fraction I. In addition, it was observed the presence of peptides, such neurotoxins, hypotensive peptides and AMPs, which probably eluted in this fraction aggregated to other venom components. Fraction I is the first to be eluted during the process of fractionation of the venom in a CMC-52 cationic resin, this fact indicates that it is composed of toxins with less basic characters than the others.

Related to ACE inhibition observed in the biochemical analysis, the proteome revealed 11% hypotensive peptides, including bradykinin-potentiating peptides, hypotensins, PAPE peptides and one endothelin-converting enzyme 1. The higher coverage of this class was obtained for the Hypotensin-2 (P84190.1), already described by Verano-Braga et al. [33], with 48 peptides sequenced that matched with the 25 amino acids residues from this template. However, the *de novo* sequencing showed 210 *de novo* tags for this template, resulted from at least 22 amino acid residues mutations (Additional file 2). Also, this sequence has 33 possible post-translational modifications, such as the formation of pyro-glutamic ring which hamper the sequencing of these peptides by Edman’s Degradation.

In this study, we identified two peptides (YANLGEFPWMVFIR and SELDKNCSGFLLSPSFVLDHK) that matched with a putative venom chymotrypsin-like protease (AG85164.1) from *Tityus bahiensis*. These findings can explain the enhancing of proteolytic activity of trypsin and chymotrypsin observed in the biochemical analysis of this fraction.

**Figure 4.**
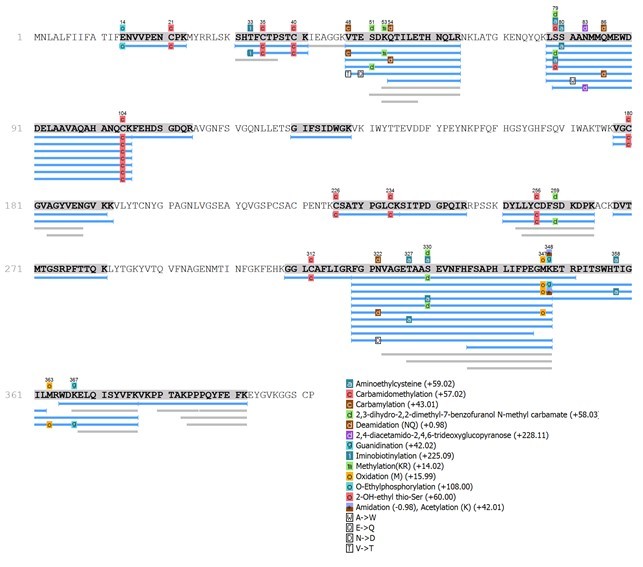
*De novo* sequencing of the CRISP identified in the Fraction I using the JAW07031.1 (putative cysteine-rich protein from *Tityus serrulatus*) as a template.

Interesting, the proteome analysis revealed the presence of several CRISPs which were never isolated before and were reported only in the proteomic study of De Oliveira et al. [32]. Herein, we found that this venom component class is eluted in Fraction I, which will be useful in directing the isolation procedure to obtain these toxins. In this study we have covered 57% of the protein sequence identified by De Oliveira et al [32] (deposit JAW07031.1). This sequence presented 14 post-translational modifications, including amidations, acetylation, among others (Fig. 4).

The proteome of Fraction I identified venom components classes that were described just in the transcriptome study of De Oliveira et al. [32], such as phospholipases A2, antimicrobial and bradykinin-potentiating peptides. This fact reinforces the importance of a previous fractionation of the venom in order to obtain a more accurate sequencing of the venom components in the proteomics studies.

## Discussion

Although the omics approaches provide a holistic (although not exhaustive) view of the venom composition from the animals, there is a gap in the obtainment of these components directly from the venom, mainly due the low yield of purified toxins from milked venom. Therefore, a proteome fraction-directed or fraction subproteomes associated to shotgun proteomics can overcome this challenge.

In this study a preliminary fractionation of the *Ts* venom was performed using the cation exchange column CMC-52, as described by Arantes et al. [9] and Cerni et al. [5]. This fractionation is based on the electrostatic interaction between the sample components and the stationary phase at pH 7.8. The positively charged venom components interact with the negatively charged carboxymethyl groups of the resin and are eluted with the increase of the buffer concentration [9]. This *Ts* venom fractionation approach has some advantages, as the isolation of Ts1 (fraction XIII), corresponding to 16% of the soluble venom. In addition, enzymes, such as hyaluronidase and proteases, especially metalloproteinases, keep their activities under these chromatographic conditions [5].

The chromatographic fractionation of the *Ts* venom resulted in 18 fractions. Fraction I is the first to be eluted, indicating that it is composed by proteins with character less basic than the other ones that are eluted with higher buffer concentrations. This fraction is rich in venom components, showing many protein bands with different molecular masses, in the Tricine-SDS-PAGE. Additionally, Fraction I corresponds to approximately 30% of the *Ts* venom, being its major fraction.

The proteolytic activity assays of fraction I in the presence of inhibitors reveal the presence of both metalloproteases (inhibited by EDTA) and serine proteases (inhibited by PMSF). The existence of these proteases in *Ts* venom was reported before in omics studies of De Oliveira et al [32] that found 16 transcripts related to trypsin-like proteases and 1 sequence of trypsin-like serine protease in the *T. serrulatus* proteome. However, the chymotrypsin peptides found in our work represents the first evidence at protein level of the presence of this protease in the *Ts* venom.

The function of scorpion venom proteases has not been fully elucidated yet. These enzymes can cleave proteins at specific sites of the amino acid sequence and are classified according to the key amino acid at the catalytic site or according to the need of the metal ion to perform its function [34]. These enzymes are important for cellular metabolism, since they participate in the post-translational process of removal of the signal peptides, among others [35].

Proteinases can also act as toxins and are well characterized in the venoms of spiders and snakes. Concerning *T. serrulatus* venom, in 2010, a metalloproteinase called antarease, showed to be able to penetrate into intact tissues and cleaves vesicle-associated membrane proteins (VAMPs), which may alter vesicular transport and secretion mechanisms [36, 37]. Thus, antarease may be one of the compounds responsible for acute pancreatitis observed after envenoming with *T. serrulatus* [36]. Recently, ten novel metalloproteinases sequences were predicted after analysis of the *T. serrulatus* venom gland transcriptome, called metaloserrulases [38].

Fraction I was also able to inhibit the ACE activity *in vitro*, and its proteome identified several components classified as hypotensive peptides. Some of these peptides are responsible for inhibiting the angiotensin converting enzyme, which converts angiotensin I to angiotensin II, a potent vasoconstrictor peptide. In addition, ACE also inactivates bradykinin, which is a vasodilator peptide [39]. These concomitant actions lead to a decrease in blood pressure, thus demonstrating the importance of these peptides as a possible molecular tool and/or therapeutic drug. Numerous studies reported already the presence of hypotensive peptides in animal’s venom [27, 40, 41].

Several scorpion venom components act on the cardiovascular system. Among them natriuretic peptides [42], PAPE peptides [18], non-disulfide-bridged peptides [43], bradykinin-potentiating peptides (BPPs) and hypotensins [33, 44] which are peptides that exhibits hypotensive properties. In our study, we found in Fraction I all these peptides, with exception of natriuretic peptide. In addition, we identified endothelin-converting enzyme, already found in the study of De Oliveira et al. [32]. Although endothelin is frequently considered as having a primarily cardiovascular role in the brain, the roles of this enzyme in the venom remains unclear [32, 45].

Another important result obtained from proteome of Fraction I was the identification of CRISP classes. This venom component has been already reported in other animal venom, such as snakes [46-48], spiders [49, 50] and scorpions [32, 51, 52]. The role of CRISPs in the venom remain unclear but they seem to interfere in the smooth-muscle contraction by inhibiting ion channels [53]. One CRISP identified in the transcriptome of *T. serrulatus* by De Oliveira [32] was sequenced in our study. These results show how subproteome can be effective to drive the researches in the isolation of components with biotechnological and therapeutic potential directly from the venom.

It is worth mentioning that some basic neurotoxins, such as Ts1, which are also found in fractions eluted at higher buffer concentrations, have been identified in Fraction I. This result shows that these neurotoxins may aggregate with other venom proteins, preventing their interactions with the resin during the chromatographic process, justifying their presence also in Fraction I. These phenomena contribute to increase the diversity of components present in this fraction, confirming our initial hypothesis that this fraction deserved to be better studied due to its diversity in bioactive components.

## Conclusion

The study of the venom components has a remarkable importance in the toxinology field, which allows identifying molecules with biotechnological and therapeutic potentials. A fraction subproteome associated to biochemical characterization has an important role in the venom elucidation. Shotgun proteomics described at least 8 different venom component classes in Fraction I, among them proteases and hypotensive peptides, which were confirmed by enzymatic assays, revealing how complex is this fraction. Venom subproteomes may serve as a roadmap to obtain specific venom components in their native forms and therefore perform its preliminary characterization, promoting advances and biological discoveries to toxinology field.

## Supplementary material

Supplementary material The following online material is available for this article:

Additional File 1: Full list of proteins and peptides identified after proteomics analysis of Fraction I from *T. serrulatus* venom against the database. Only peptides with −10lgP > 20 were used to match the proteins in this study.

Additional File 2: Hypotensin-2 (P84190.1) found in the Fraction I, in which the *de novo* sequencing showed 210 *de novo tags* resulted from at least 22 amino acid residues mutations.
