# Supplementary material for: Proteome of fraction from Tityus serrulatus venom reveals new enzymes and toxins
Source: J Venom Anim Toxins Incl Trop Dis. 2019 Apr 18;25:e148218. doi: 10.1590/1678-9199-JVATITD-1482-18 (PMC6483408; doi:10.1590/1678-9199-JVATITD-1482-18)
Supplement: Additional File 1: [file 1678-9199-jvatitd-25-e148218-s1.pdf]

Supplementary Material to "Proteome of fraction from *Tityus serrulatus* venom reveals new enzymes and toxins"

Additional file 1 - Full list of proteins and peptides identified after proteomics analysis of Fraction I from *T. serrulatus* venom against the database. Only peptides with −10lgP > 20 were used to match the proteins in this study.

Protein List

Protein Group	Protein ID	Accession	-10lgP	Coverage (%)	Coverage (%) Sample 1	Intensity Sample 1	#Peptides	#Unique	#Spec Sample 1	PTM	Avg. Mass	Description
**3**	60	P84190.1	214,15	100	100	1,28E+09	48	12	2252	Amidation; Carbamylation; Methylation(KR); Ubiquitin; Sodium adduct; 29 more	2726	RecName: Full=Hypotensin-2; AltName: Full=Hypotensin II; Short=TsHpt-II; Contains: RecName: Full=Hypotensin-4; AltName: Full=Hypotensin IV; Short=TsHpt-IV
**5**	110	P86824.1	152,49	100	100	3,08E+10	8	8	507	Amidation; Ubiquitin; Sodium adduct; Replacement of proton by potassium; Formylation (Protein N-term); 5 more	1465	RecName: Full=Hypotensin-like peptide
**4**	29	P86821.1	248,15	92	92	3,17E+10	56	44	1710	Sodium adduct; Carboxylation (E); Replacement of proton with ammonium ion; Replacement of proton by potassium; Formylation (Protein N-term); 8 more	3767	RecName: Full=Pape peptide
**9**	23	P68411.1	228,29	92	92	2,23E+10	16	12	343	Carbamidomethylation; Oxidation (M); Carbamylation; Methylation(KR); Ubiquitin; 5 more	6998	RecName: Full=Toxin Tst2; AltName: Full=P-Mice-beta* NaTx5.2; AltName: Full=Tityustoxin II; Short=TsTX-II; AltName: Full=Toxin III-8

Protein Group	Protein ID	Accession	-10lgP	Coverage (%)	Coverage (%) Sample 1	Intensity Sample 1	#Peptides	#Unique	#Spec Sample 1	PTM	Avg. Mass	Description
**9**	24	1913435A	228,29	92	92	2,23E+10	16	12	343	Carbamidomethylation; Oxidation (M); Carbamylation; Methylation(KR); Ubiquitin; 5 more	6998	beta toxin TsII
**9**	25	P68410.1	228,29	92	92	2,23E+10	16	12	343	Carbamidomethylation; Oxidation (M); Carbamylation; Methylation(KR); Ubiquitin; 5 more	6998	RecName: Full=Alpha-mammal toxin Ts2; AltName: Full=P-Mice-beta* NaTx5.1; AltName: Full=Tityustoxin II; Short=Toxin II; Short=TsTX-II; Short=Tst2; AltName: Full=Toxin T1-IV; AltName: Full=TsTX III-8; AltName: Full=TsTX-III
**9**	26	AAB36322.1	228,29	92	92	2,23E+10	16	12	343	Carbamidomethylation; Oxidation (M); Carbamylation; Methylation(KR); Ubiquitin; 5 more	6998	III-8st=toxin III-8 homolog [Tityus stigmurus venom Peptide 62 aa]
**14**	86	P60276.1	111,36	92	92	0,00E+00	8	4	182	Carbamidomethylation; Oxidation (M); Amidation; Carbamylation; Methylation(KR); 6 more	6963	RecName: Full=Toxin Tb2-II; AltName: Full=P-Mice-Ins-beta* NaTx5.4
**7**	35	P0C8W6.1	135,33	91	91	2,28E+07	33	21	805	Methylation(KR); Sodium adduct; Dihydroxy; Alpha-amino adipic acid; Carboxylation (E); 15 more	4555	RecName: Full=Pape peptide
**19**	37	Q0GY42.1	161,12	79	79	0,00E+00	14	4	111	Carbamidomethylation; Ubiquitin; Nucleophilic addition to cytopiloyne+H2O; Naphthalene-2 3-dicarboxaldehyde	5284	RecName: Full=Potassium channel toxin TcoKIK
**19**	38	ABE98265.1	161,12	79	79	0,00E+00	14	4	111	Carbamidomethylation; Ubiquitin; Nucleophilic addition to cytopiloyne+H2O; Naphthalene-2 3-dicarboxaldehyde	5284	KIK partial [Tityus costatus]
**34**	47	1NPI	151,26	77	77	3,49E+06	9	6	14	Carbamidomethylation; Alpha-amino adipic acid; 2 3-dihydro-2 2-dimethyl-7-benzofuranol N-methyl carbamate; Aminoethylcysteine; O-Diisopropylphosphorylation; LeudimethylArgGlyGly	6891	Chain A Toxin Vii
**34**	48	1B7D	151,26	77	77	3,49E+06	9	6	14	Carbamidomethylation; Alpha-amino adipic acid; 2 3-dihydro-2 2-dimethyl-7-benzofuranol N-methyl carbamate; Aminoethylcysteine; O-	6891	Chain A Neurotoxin (Ts1) From Brazilian Scorpion Tityus Serrulatus

Protein Group	Protein ID	Accession	-10lgP	Coverage (%)	Coverage (%) Sample 1	Intensity Sample 1	#Peptides	#Unique	#Spec Sample 1	PTM	Avg. Mass	Description
										Diisopropylphosphorylation; LeudimethylArgGlyGly		
**34**	49	AAB36321.1	151,26	76	76	3,49E+06	9	6	14	Carbamidomethylation; Alpha-amino adipic acid; 2 3-dihydro-2 2-dimethyl-7-benzofuranol N-methyl carbamate; Aminoethylcysteine; O-Diisopropylphosphorylation; LeudimethylArgGlyGly	6992	gamma-st=toxin gamma homolog [Tityus stigmurus venom Peptide 62 aa]
**6**	12	AAC25688.1	278,68	74	74	2,94E+10	39	33	882	Carbamidomethylation; Amidation; Carbamylation; Methylation(KR); Ubiquitin; 23 more	9176	immunogenic venom protein TsnTxp [Tityus serrulatus]
**6**	13	O77463.1	278,68	74	74	2,94E+10	39	33	882	Carbamidomethylation; Amidation; Carbamylation; Methylation(KR); Ubiquitin; 23 more	9176	RecName: Full=Toxin Ts4; AltName: Full=Non-toxic protein TsNTxP; Short=NTxP; AltName: Full=P-Allerg-alpha* NaTx4.1; AltName: Full=PT-Immun-alpha* NaTx4.2; AltName: Full=Tityustoxin VI; Short=Ts VI; Short=TsTX-VI; Short=TsTXVI; Shor...
**6**	14	AAC25689.1	278,68	74	74	2,94E+10	39	33	882	Carbamidomethylation; Amidation; Carbamylation; Methylation(KR); Ubiquitin; 23 more	9176	immunogenic protein NTxp [Tityus serrulatus]
**34**	21	AAW72453.1	159,13	73	73	0,00E+00	10	5	35	Carbamidomethylation; Oxidation (M); Carbamylation; Methylation(KR); Ubiquitin; 5 more	7254	putative long-chain toxin partial [Tityus costatus]
**34**	22	Q5G8B8.1	159,13	73	73	0,00E+00	10	5	35	Carbamidomethylation; Oxidation (M); Carbamylation; Methylation(KR); Ubiquitin; 5 more	7254	RecName: Full=Toxin Tco-gamma; AltName: Full=T-beta* NaTx6.4; AltName: Full=Toxin gamma-like
**12**	58	P0C2F3.1	198,62	70	70	5,09E+09	10	7	232	Carbamidomethylation; Amidation; Deamidation (NQ); Dehydration; Carboxymethyl (KW X@N-term); 2 more	6723	RecName: Full=Potassium channel toxin Tst-beta-KTx; Short=TstbetaKTx
**9**	27	JAW07016.1	230,09	69	69	2,23E+10	17	12	339	Carbamidomethylation; Carbamylation; Methylation(KR); Ubiquitin; 2 3-dihydro-2 2-dimethyl-7-benzofuranol N-methyl carbamate; 3 more	9349	putative sodium channel toxin [Tityus serrulatus]

Protein Group	Protein ID	Accession	-10lgP	Coverage (%)	Coverage (%) Sample 1	Intensity Sample 1	#Peptides	#Unique	#Spec Sample 1	PTM	Avg. Mass	Description
**9**	28	JAW06979.1	230,09	69	69	2,23E+10	17	12	339	Carbamidomethylation; Carbamylation; Methylation(KR); Ubiquitin; 2 3-dihydro-2 2-dimethyl-7-benzofuranol N-methyl carbamate; 3 more	9349	putative sodium channel toxin [Tityus serrulatus]
**14**	87	C0HJM9.1	115,09	69	69	0,00E+00	9	4	178	Carbamidomethylation; Amidation; Carbamylation; Ubiquitin; Deamidation (NQ); 4 more	9413	RecName: Full=Toxin Tf2; Flags: Precursor
**14**	88	JAG85185.1	115,09	69	69	0,00E+00	9	4	178	Carbamidomethylation; Amidation; Carbamylation; Ubiquitin; Deamidation (NQ); 4 more	9314	putative sodium channel toxin [Tityus bahiensis]
**14**	89	CEF39517.1	115,09	69	69	0,00E+00	9	4	178	Carbamidomethylation; Amidation; Carbamylation; Ubiquitin; Deamidation (NQ); 4 more	9413	Tf2 [Tityus fasciolatus]
**27**	93	JAW07006.1	180,06	66	66	7,25E+07	12	12	61	Carbamidomethylation; 2 3-dihydro-2 2-dimethyl-7-benzofuranol N-methyl carbamate; Deamidation (NQ); O-Diisopropylphosphorylation; Dehydroalanine (Y); 2 more	10485	putative sodium channel toxin partial [Tityus serrulatus]
**2**	44	JAW06987.1	234,34	64	64	1,23E+09	60	16	2460	Amidation; Ammonia-loss (N); Carbamylation; Methylation(KR); Ubiquitin; 43 more	7774	putative hypotensin [Tityus serrulatus]
**2**	45	JAW07038.1	234,34	61	61	1,23E+09	60	16	2460	Amidation; Ammonia-loss (N); Carbamylation; Methylation(KR); Ubiquitin; 43 more	8164	putative hypotensin [Tityus serrulatus]
**2**	46	JAW06992.1	234,34	61	61	1,23E+09	60	16	2460	Amidation; Ammonia-loss (N); Carbamylation; Methylation(KR); Ubiquitin; 43 more	8164	putative hypotensin [Tityus serrulatus]
**8**	1	JAW06995.1	256,65	59	59	4,23E+09	27	22	465	Carbamidomethylation; Oxidation (M); Amidation; Carbamylation; Methylation(KR); 10 more	44083	putative cysteine-rich protein [Tityus serrulatus]
**8**	2	JAW07031.1	256,65	57	57	4,23E+09	27	22	465	Carbamidomethylation; Oxidation (M); Amidation; Carbamylation; Methylation(KR); 10 more	44870	putative cysteine-rich protein [Tityus serrulatus]
**34**	50	AAB29128.1	151,26	56	56	3,49E+06	9	6	14	Carbamidomethylation; Alpha-amino adipic acid; 2 3-dihydro-2 2-dimethyl-7-benzofuranol N-methyl carbamate; Aminoethylcysteine; O-	9382	toxin gamma [Tityus serrulatus]

Protein Group	Protein ID	Accession	-10lgP	Coverage (%)	Coverage (%) Sample 1	Intensity Sample 1	#Peptides	#Unique	#Spec Sample 1	PTM	Avg. Mass	Description
										Diisopropylphosphorylation; LeudimethylArgGlyGly		
**34**	51	P56612.1	151,26	56	56	3,49E+06	9	6	14	Carbamidomethylation; Alpha-amino adipic acid; 2 3-dihydro-2 2-dimethyl-7-benzofuranol N-methyl carbamate; Aminoethylcysteine; O-Diisopropylphosphorylation; LeudimethylArgGlyGly	9366	RecName: Full=Toxin Tst1; AltName: Full=PT-Mice-beta* NaTx6.3; AltName: Full=Tst-gamma; Flags: Precursor
**34**	52	AIA81145.1	151,26	56	56	3,49E+06	9	6	14	Carbamidomethylation; Alpha-amino adipic acid; 2 3-dihydro-2 2-dimethyl-7-benzofuranol N-methyl carbamate; Aminoethylcysteine; O-Diisopropylphosphorylation; LeudimethylArgGlyGly	9438	toxin 1 gamma-like protein [Tityus trivittatus]
**34**	53	CAA46982.1	151,26	56	56	3,49E+06	9	6	14	Carbamidomethylation; Alpha-amino adipic acid; 2 3-dihydro-2 2-dimethyl-7-benzofuranol N-methyl carbamate; Aminoethylcysteine; O-Diisopropylphosphorylation; LeudimethylArgGlyGly	9382	Ts VII [Tityus serrulatus]
**34**	54	P15226.2	151,26	56	56	3,49E+06	9	6	14	Carbamidomethylation; Alpha-amino adipic acid; 2 3-dihydro-2 2-dimethyl-7-benzofuranol N-methyl carbamate; Aminoethylcysteine; O-Diisopropylphosphorylation; LeudimethylArgGlyGly	9382	RecName: Full=Beta-mammal/insect toxin Ts1; AltName: Full=PT-Mice-Ins-beta NaTx6.1; AltName: Full=Tityustoxin VII; Short=Ts VII; Short=Ts7; Short=TsTX-VII; AltName: Full=Toxin II-11; AltName: Full=Toxin III-10; AltName: Full=Toxin ...
**34**	55	P0DMM8.1	151,26	56	56	3,49E+06	9	6	14	Carbamidomethylation; Alpha-amino adipic acid; 2 3-dihydro-2 2-dimethyl-7-benzofuranol N-methyl carbamate; Aminoethylcysteine; O-Diisopropylphosphorylation; LeudimethylArgGlyGly	9438	RecName: Full=Beta-mammal Tt1g; AltName: Full=T.trivittatus toxin 1 gamma-like; Flags: Precursor
**34**	77	AAB36318.1	144,5	56	56	3,49E+06	8	5	13	Carbamidomethylation; Alpha-amino adipic acid; 2 3-dihydro-2 2-dimethyl-7-benzofuranol N-methyl carbamate;	6893	gamma-b=toxin gamma homolog [Tityus bahiensis venom Peptide 61 aa]

Protein Group	Protein ID	Accession	-10lgP	Coverage (%)	Coverage (%) Sample 1	Intensity Sample 1	#Peptides	#Unique	#Spec Sample 1	PTM	Avg. Mass	Description
										Aminoethylcysteine; O-Diisopropylphosphorylation; LeudimethylArgGlyGly		
**16**	3	AIJ02110.2	264,57	48	48	7,33E+09	30	17	160	Carbamidomethylation; Carbamylation; Methylation(KR); Sodium adduct; 2 3-dihydro-2 2-dimethyl-7-benzofuranol N-methyl carbamate; 10 more	44055	metalloserrulase 2 [Tityus serrulatus]
**29**	30	P86392.3	199,65	48	48	2,45E+09	12	12	44	Carbamidomethylation; Oxidation (M); Amidation; Carbamylation; Methylation(KR); 6 more	25390	RecName: Full=Venom metalloproteinase antarease TserMP_A; Short=VMPA
**29**	31	V9Z9A3.1	199,65	48	48	2,45E+09	12	12	44	Carbamidomethylation; Oxidation (M); Amidation; Carbamylation; Methylation(KR); 6 more	25445	RecName: Full=Venom metalloproteinase antarease-like TserMP_B; Short=VMPA
**29**	32	AHE40589.1	199,65	48	48	2,45E+09	12	12	44	Carbamidomethylation; Oxidation (M); Amidation; Carbamylation; Methylation(KR); 6 more	25445	antarease-like Zn-metalloprotease partial [Tityus serrulatus]
**29**	33	AHE40588.1	199,65	48	48	2,45E+09	12	12	44	Carbamidomethylation; Oxidation (M); Amidation; Carbamylation; Methylation(KR); 6 more	25390	antarease-like Zn-metalloprotease partial [Tityus serrulatus]
**12**	59	P69940.2	198,62	48	48	5,09E+09	10	7	232	Carbamidomethylation; Amidation; Deamidation (NQ); Dehydration; Carboxymethyl (KW X@N-term); 2 more	9728	RecName: Full=Potassium channel toxin TsTXK-beta; AltName: Full=Potassium channel toxin beta-KTx 1; AltName: Full=Tityustoxin K-beta; Short=TsTX K beta; Short=TsTX-K beta; Short=TsTXKbeta; AltName: Full=Ts8; AltName: Full=TsK2; Fla...
**15**	68	Q5G8A6.1	107,03	48	48	0,00E+00	7	4	161	Carbamidomethylation; Carboxylation (DKW); Dehydration; Oxidation to nitro	9776	RecName: Full=Scorpine-like peptide Tco 41.46-2; Contains: RecName: Full=Scorpine-like peptide Tco 42.14; AltName: Full=Tco-beta-KTx; Short=TcobetaKTx; Flags: Precursor
**15**	69	JAG85216.1	107,03	48	48	0,00E+00	7	4	161	Carbamidomethylation; Carboxylation (DKW); Dehydration; Oxidation to nitro	9776	putative potassium channel toxin [Tityus bahiensis]

Protein Group	Protein ID	Accession	-10lgP	Coverage (%)	Coverage (%) Sample 1	Intensity Sample 1	#Peptides	#Unique	#Spec Sample 1	PTM	Avg. Mass	Description
**15**	70	AAW72464.1	107,03	48	48	0,00E+00	7	4	161	Carbamidomethylation; Carboxylation (DKW); Dehydration; Oxidation to nitro	9760	scorpine-like peptide precursor [Tityus costatus]
**15**	71	AAW72465.1	107,03	48	48	0,00E+00	7	4	161	Carbamidomethylation; Carboxylation (DKW); Dehydration; Oxidation to nitro	9776	scorpine-like peptide precursor [Tityus costatus]
**20**	8	JAG85213.1	215,37	46	46	0,00E+00	20	7	111	Carbamidomethylation; Carbamylation; Methylation(KR); Sodium adduct; Phosphorylation (STY); 12 more	43397	putative metalloproteinase partial [Tityus bahiensis]
**4**	16	JAW07022.1	266,69	44	44	3,17E+10	88	76	1837	Amidation; Methylation(KR); Sodium adduct; Dihydroxy; Carboxylation (E); 33 more	9838	putative antimicrobial peptide [Tityus serrulatus]
**34**	78	JAG85210.1	144,5	44	44	3,49E+06	8	5	13	Carbamidomethylation; Alpha-amino adipic acid; 2 3-dihydro-2 2-dimethyl-7-benzofuranol N-methyl carbamate; Aminoethylcysteine; O-Diisopropylphosphorylation; LeudimethylArgGlyGly	8711	putative sodium channel toxin partial [Tityus bahiensis]
**11**	40	P86822.2	207,83	41	41	5,00E+09	17	7	247	Carbamidomethylation; 2 3-dihydro-2 2-dimethyl-7-benzofuranol N-methyl carbamate; Deamidation (NQ); Nucleophilic addition to cytopiloyne+H2O; Ornithine from Arginine; 3 more	10083	RecName: Full=Ts19; AltName: Full=Potassium channel toxin beta-Ktx 2; Contains: RecName: Full=Ts19 fragment I; AltName: Full=Potassium channel toxin beta-Ktx 2-1; Contains: RecName: Full=Ts19 fragment II; Short=Ts19 Frag-II; AltNam...
**24**	4	JAW07025.1	187,7	41	41	1,97E+09	13	12	77	Carbamidomethylation; Ubiquitin; 2 3-dihydro-2 2-dimethyl-7-benzofuranol N-methyl carbamate; Mutation	37728	putative metalloproteinase [Tityus serrulatus]
**19**	39	JAG85223.1	161,12	41	41	0,00E+00	14	4	111	Carbamidomethylation; Ubiquitin; Nucleophilic addition to cytopiloyne+H2O; Naphthalene-2 3-dicarboxaldehyde	10068	putative potassium channel toxin [Tityus bahiensis]
**34**	79	P56611.1	144,5	40	40	3,49E+06	8	5	13	Carbamidomethylation; Alpha-amino adipic acid; 2 3-dihydro-2 2-dimethyl-7-benzofuranol N-methyl carbamate; Aminoethylcysteine; O-	9384	RecName: Full=Toxin Tb1; AltName: Full=PT-Mice-beta* NaTx6.2; AltName: Full=Tb-gamma; Flags: Precursor

Protein Group	Protein ID	Accession	-10lgP	Coverage (%)	Coverage (%) Sample 1	Intensity Sample 1	#Peptides	#Unique	#Spec Sample 1	PTM	Avg. Mass	Description
										Diisopropylphosphorylation; LeudimethylArgGlyGly		
**34**	80	JAG85191.1	144,5	40	40	3,49E+06	8	5	13	Carbamidomethylation; Alpha-amino adipic acid; 2 3-dihydro-2 2-dimethyl-7-benzofuranol N-methyl carbamate; Aminoethylcysteine; O-Diisopropylphosphorylation; LeudimethylArgGlyGly	9384	putative sodium channel toxin [Tityus bahiensis]
**21**	65	ABV64385.1	109,8	38	38	0,00E+00	6	2	90	Carbamidomethylation; Carbamylation; Deamidation (NQ)	10067	long-chain beta-KTx-like toxin [Tityus trivittatus]
**21**	66	ABE98262.1	109,8	38	38	0,00E+00	6	2	90	Carbamidomethylation; Carbamylation; Deamidation (NQ)	10067	KIK [Tityus trivittatus]
**21**	67	Q0GY45.1	109,8	38	38	0,00E+00	6	2	90	Carbamidomethylation; Carbamylation; Deamidation (NQ)	10067	RecName: Full=Potassium channel toxin TtrKIK; Flags: Precursor
**24**	9	JAW06984.1	178,71	37	37	1,83E+09	13	12	56	Carbamidomethylation; Ubiquitin; 2 3-dihydro-2 2-dimethyl-7-benzofuranol N-methyl carbamate; Mutation	42121	putative metalloproteinase partial [Tityus serrulatus]
**5**	72	JAW07002.1	205,3	36	36	3,14E+10	24	20	881	Amidation; Ubiquitin; Sodium adduct; Phosphorylation (STY); Dihydroxy; 10 more	8033	putative hypotensin [Tityus serrulatus]
**24**	5	AMO02558.1	187,7	35	35	1,97E+09	13	12	77	Carbamidomethylation; Ubiquitin; 2 3-dihydro-2 2-dimethyl-7-benzofuranol N-methyl carbamate; Mutation	44932	metalloserrulase 16 [Tityus serrulatus]
**25**	11	JAW07039.1	171,75	35	35	0,00E+00	13	2	72	Carbamidomethylation; Phosphorylation (STY); Dihydroxy; Deamidation (NQ); Acetylation (TSCYH); 2 more	44723	putative metalloproteinase [Tityus serrulatus]
**17**	19	JAG85161.1	161,66	35	35	0,00E+00	12	7	117	Carbamidomethylation; Oxidation (M); 2 3-dihydro-2 2-dimethyl-7-benzofuranol N-methyl carbamate; Deamidation (NQ); Dimethylation(KR); 4 more	44944	putative venom cysteine-rich protein [Tityus bahiensis]
**11**	62	P0C8W4.2	206,18	34	34	5,00E+09	16	7	230	Carbamidomethylation; 2 3-dihydro-2 2-dimethyl-7-benzofuranol N-methyl carbamate; Deamidation (NQ); Nucleophilic addition to cytopiloyne+H2O; Ornithine from Arginine; 3 more	10097	RecName: Full=Potassium channel toxin TstKMK; AltName: Full=Toxin 5536; AltName: Full=beta-Ktx; Flags: Precursor

Protein Group	Protein ID	Accession	-10lgP	Coverage (%)	Coverage (%) Sample 1	Intensity Sample 1	#Peptides	#Unique	#Spec Sample 1	PTM	Avg. Mass	Description
**11**	63	AFP87551.1	206,18	34	34	5,00E+09	16	7	230	Carbamidomethylation; 2 3-dihydro-2 2-dimethyl-7-benzofuranol N-methyl carbamate; Deamidation (NQ); Nucleophilic addition to cytopiloyne+H2O; Ornithine from Arginine; 3 more	10097	potassium channel blocker-like toxin beta-KTx [Tityus stigmurus]
**42**	42	JAW06978.1	134,71	34	34	0,00E+00	5	5	8	Carbamidomethylation; 2 3-dihydro-2 2-dimethyl-7-benzofuranol N-methyl carbamate; Aminoethylcysteine; Mutation	19350	putative phospholipase A2 partial [Tityus serrulatus]
**36**	108	JAW06986.1	112,74	34	34	0,00E+00	5	5	7	Carbamidomethylation; Phenethyl isothiocyanate; Mutation	22348	putative cysteine-rich protein partial [Tityus serrulatus]
**29**	18	AIJ02109.2	201,95	33	33	2,45E+09	13	13	45	Carbamidomethylation; Oxidation (M); Amidation; Carbamylation; Methylation(KR); 6 more	42961	metalloserrulase 1 [Tityus serrulatus]
**35**	130	AAW72463.1	57,45	33	33	4,00E+09	3	2	13	Carbamidomethylation; Mutation	9036	putative long-chain toxin precursor [Tityus costatus]
**35**	131	Q5G8A8.1	57,45	33	33	4,00E+09	3	2	13	Carbamidomethylation; Mutation	9036	RecName: Full=Toxin-like TcoNTxP1; AltName: Full=Insect-like toxic peptide Tco 38.32-2; AltName: Full=PT-alpha* NaTx4.5; Flags: Precursor
**26**	10	JAG85190.1	155,55	32	32	0,00E+00	11	2	74	Carbamidomethylation; Oxidation (M); Phosphorylation (STY); 2 3-dihydro-2 2-dimethyl-7-benzofuranol N-methyl carbamate; Deamidation (NQ); 2 more	44081	putative metalloproteinase partial [Tityus bahiensis]
**23**	97	Q0GY43.1	98,84	32	32	0,00E+00	6	3	83	Carbamidomethylation; Monoglutamyl; Glycidamide adduct	9960	RecName: Full=Potassium channel toxin TdiKIK; Short=TdKIK; Flags: Precursor
**23**	98	ABE98264.1	98,84	32	32	0,00E+00	6	3	83	Carbamidomethylation; Monoglutamyl; Glycidamide adduct	9960	KIK [Tityus discrepans]
**32**	107	XP_023239442.1	156,01	30	30	1,84E+08	11	11	40	Carbamidomethylation; Carbamylation; 2 3-dihydro-2 2-dimethyl-7-benzofuranol N-methyl carbamate; Mutation	39135	peptidylglycine alpha-hydroxylating monooxygenase-like [Centruroides sculpturatus]
**42**	43	JAW07019.1	134,71	28	28	0,00E+00	5	5	8	Carbamidomethylation; 2 3-dihydro-2 2-dimethyl-7-benzofuranol N-methyl	23693	putative phospholipase A2 [Tityus serrulatus]

Protein Group	Protein ID	Accession	-10lgP	Coverage (%)	Coverage (%) Sample 1	Intensity Sample 1	#Peptides	#Unique	#Spec Sample 1	PTM	Avg. Mass	Description
										carbamate; Aminoethylcysteine; Mutation		
**54**	109	XP_023218922.1	79,29	28	28	0,00E+00	2	2	2	Carbamidomethylation; Sodium adduct	16085	lysozyme-like [Centruroides sculpturatus]
**31**	15	AIJ02113.2	186,3	26	26	7,89E+08	11	11	39	Carbamidomethylation; 2 3-dihydro-2 2-dimethyl-7-benzofuranol N-methyl carbamate; Mutation	45218	metalloserrulase 5 [Tityus serrulatus]
**43**	95	JAG85215.1	65,19	25	25	9,36E+08	6	6	15	Amidation	10712	putative BPP-like protein [Tityus bahiensis]
**18**	20	AIJ02112.2	197,51	21	21	6,90E+08	10	10	109	Carbamidomethylation; Carbamylation; Methylation(KR); Sodium adduct; 2 3-dihydro-2 2-dimethyl-7-benzofuranol N-methyl carbamate; 4 more	44149	metalloserrulase 4 [Tityus serrulatus]
**39**	101	AIJ02111.3	107,26	21	21	0,00E+00	5	5	11	Carbamidomethylation; Sodium adduct; 2 3-dihydro-2 2-dimethyl-7-benzofuranol N-methyl carbamate; Dimethylation of proline residue; N-Succinimidyl-2-morpholine acetate; Mutation	43693	metalloserrulase 3 partial [Tityus serrulatus]
**22**	61	JAW07042.1	103,02	18	18	0,00E+00	6	2	51	Phosphorylation (STY); Deamidation (NQ); Heme	45537	putative metalloproteinase partial [Tityus serrulatus]
**30**	85	XP_023242894.1	160,97	18	18	0,00E+00	11	11	45	Ethylation; EDT; Dimethylation of proline residue; Mutation	45939	ectonucleotide pyrophosphatase/phosphodiesterase family member 5-like [Centruroides sculpturatus]
**36**	81	JAW07036.1	130,66	16	16	0,00E+00	7	7	12	Carbamidomethylation; Sodium adduct; Phenethyl isothiocyanate; Mutation	53185	cysteine-rich perotein [Tityus serrulatus]
**44**	82	JAW06991.1	134,2	15	15	1,83E+08	6	6	6	Carbamidomethylation; Deamidation (NQ); Mutation	47350	putative cysteine-rich protein [Tityus serrulatus]
**44**	84	JAW07032.1	134,2	15	15	1,83E+08	6	6	6	Carbamidomethylation; Deamidation (NQ); Mutation	47350	putative cysteine-rich protein [Tityus serrulatus]
**29**	75	AHE40596.1	81,57	13	13	0,00E+00	3	3	12	Carbamidomethylation; Mutation	43062	antarease-like Zn-metalloprotease [Tityus trivittatus]
**29**	76	V9ZAY0.1	81,57	13	13	0,00E+00	3	3	12	Carbamidomethylation; Mutation	43062	RecName: Full=Venom metalloproteinase antarease-like TtrivMP_A; Short=VMPA; Flags: Precursor

Protein Group	Protein ID	Accession	-10lgP	Coverage (%)	Coverage (%) Sample 1	Intensity Sample 1	#Peptides	#Unique	#Spec Sample 1	PTM	Avg. Mass	Description
**29**	64	JAG85220.1	82,78	10	10	0,00E+00	3	3	12	Carbamidomethylation	42480	putative metalloproteinase partial [Tityus bahiensis]
**41**	114	JAW07023.1	101,34	10	10	7,38E+06	4	4	8	Amidation; Carbamylation; Deamidation (NQ)	32741	putative phospholipase A2 partial [Tityus serrulatus]
**37**	36	AIJ02118.2	163,52	8	8	1,18E+09	9	9	11	Replacement of proton with ammonium ion; Formylation; Mutation	97837	endothelin-converting enzyme 1 [Tityus serrulatus]
**44**	83	JAG85159.1	107,97	7	7	1,83E+08	4	4	4	Mutation	47590	putative cysteine-rich venom protein [Tityus bahiensis]
**47**	94	AMO02560.1	71,46	7	7	0,00E+00	2	2	4		43885	metalloserrulase 18 [Tityus serrulatus]
**48**	116	JAG85164.1	71,47	5	5	0,00E+00	2	2	3	O-Diisopropylphosphorylation	29546	putative venom chymotrypsin-like proteinase partial [Tityus bahiensis]
**30**	104	XP_023242898.1	61,02	3	3	0,00E+00	2	2	3		46644	ectonucleotide pyrophosphatase/phosphodiesterase family member 5-like [Centruroides sculpturatus]

Peptide List

Peptide	-10lgP	Mass	Length	ppm	m/z	RT	Intensity Sample 1	Frac.	Scan	Source File	#Spec	#Spec Sample 1	Accession	PTM	AScore
**TQFGC(+57.02)PAYEGYC(+57.02)NDHC(+57.02)NDIER**	93,89	2605,006	21	-1,1	869,3416	20,53	3,02E+09	1	6861	150211-Fr_I_Helo-red-alk-tryp-001.raw	26	26	P0C2F3.1:P69940.2	Carbamidomethylation	C5:Carbamidomethylation:1000.00;C12:Carbamidomethylation:1000.00;C16:Carbamidomethylation:1000.00
**FSC(+57.02)FIRPAGFC(+57.02)DGYC(+57.02)K**	89,91	1983,843	16	-0,7	992,928	21,18	3,88E+09	1	7100	150211-Fr_I_Helo-red-alk-tryp-001.raw	131	131	JAW07016.1:JAW06979.1:P68411.1:1913435A:P68410.1:AAB36322.1	Carbamidomethylation	C3:Carbamidomethylation:1000.00;C11:Carbamidomethylation:1000.00;C15:Carbamidomethylation:1000.00
**FGPNVAGETAASEVNFHFSAPHLIFPEGMK**	88,77	3200,539	30	-1	801,1412	51,2	1,03E+09	1	17590	150211-Fr_I_Helo-red-alk-tryp-001.raw	3	3	JAW07031.1:JAW06995.1		
**KLGELQDDGTVAYNTAGISLGSGVC(+57.02)K**	87,65	2652,306	26	-0,5	885,1089	36	1,97E+09	1	12195	150211-Fr_I_Helo-red-alk-tryp-001.raw	2	2	AIJ02110.2:JAG85213.1	Carbamidomethylation	C25:Carbamidomethylation:1000.00
**TEPSFIKDNLIPGPPAAFDPDVLISAMSK**	85,76	3069,573	29	-0,4	1024,198	56,92	4,48E+08	1	19475	150211-Fr_I_Helo-red-alk-tryp-001.raw	11	11	AIJ02109.2:P86392.3:V9Z9A3.1:AHE40589.1:AHE40588.1		
**QNEPSFIEDSAIPGHQQVLDPVDLVK**	85,27	2874,44	26	-0,1	959,1539	48,47	6,37E+09	1	16667	150211-Fr_I_Helo-red-alk-tryp-001.raw	10	10	AIJ02110.2		
**GSSGYC(+57.02)AWPAC(+57.02)YC(+57.02)YGLPDSVK**	83,84	2396,986	21	0,2	1199,501	45,12	1,61E+09	1	15503	150211-Fr_I_Helo-red-alk-tryp-001.raw	30	30	AAC25688.1:O77463.1:AAC25689.1	Carbamidomethylation	C6:Carbamidomethylation:1000.00;C11:Carbamidomethylation:1000.00;C13:Carbamidomethylation:1000.00
**LTSQSEYAC(+57.02)PAIDKFC(+57.02)EDHC(+57.02)AAK**	83,35	2700,162	23	0	676,0477	15,63	3,59E+09	1	5006	150211-Fr_I_Helo-red-alk-tryp-001.raw	14	14	P86822.2:P0C8W4.2:AFP87551.1	Carbamidomethylation	C9:Carbamidomethylation:1000.00;C16:Carbamidomethylation:1000.00;C20:Carbamidomethylation:1000.00
**K(+43.01)(+14.02)GSSGYC(+57.02)AWPAC(+57.02)YC(+57.02)YGLPDSVK**	83,04	2582,103	22	-1,3	1292,057	37,46	3,00E+09	1	12734	150211-Fr_I_Helo-red-alk-tryp-001.raw	3	3	AAC25688.1:O77463.1:AAC25689.1	Carbamylation; Methylation(KR); Carbamidomethylation	K1:Carbamylation:227.33;K1:Methylation(KR):227.33;C7:Carbamidomethylation:1000.00;C12:Carbamidomethylation:1000.00;C14:Carbamidomethylation:1000.00
**ASSGYC(+57.02)AWPAC(+57.02)YC(+57.02)YGVPDHIK**	82,33	2461,029	21	-1,1	1231,52	36,77	9,39E+09	1	12481	150211-Fr_I_Helo-red-alk-tryp-001.raw	19	19	JAW07016.1:JAW06979.1:P68411.1:1913435A:P68410.1:AAB36322.1	Carbamidomethylation	C6:Carbamidomethylation:1000.00;C11:Carbamidomethylation:1000.00;C13:Carbamidomethylation:1000.00
**Q(-17.03)NEPSFIEDSAIPGHQQVLDPVDLVK**	81,94	2857,413	26	-0,3	953,4781	48,89	6,41E+08	1	16810	150211-Fr_I_Helo-red-alk-tryp-001.raw	1	1	AIJ02110.2	Pyro-glu from Q	Q1:Pyro-glu from Q:1000.00
**SDVTGGFPVN(+.98)SNNC(+57.02)IYPC(+57.02)YSTQDEIQC(+57.02)EEFC(+57.02)EK**	81,01	3947,591	33	3,3	1316,875	44,42	0	1	15256	150211-Fr_I_Helo-red-alk-tryp-001.raw	3	3	JAW07006.1	Deamidation (NQ); Carbamidomethylation	N10:Deamidation (NQ):9.40;C14:Carbamidomethylation:1000.00;C18:Carbamidomethylation:1000.00;C27:C

															arbamidomethylation:1000.00;C31:Carbamidomethylation:1000.00
**KGSSGYC(+57.02)AWPAC(+57.02)YC(+57.02)YGLPDSVK**	81	2525,081	22	0,2	842,7012	37,59	8,03E+09	1	12780	150211-Fr_I_Helo-red-alk-tryp-001.raw	45	45	AAC25688.1:O77463.1:AAC25689.1	Carbamidomethylation	C7:Carbamidomethylation:1000.00;C12:Carbamidomethylation:1000.00;C14:Carbamidomethylation:1000.00
**ITC(+57.02)FLTAAGYC(+57.02)NTEC(+57.02)TLK**	80,52	2121,953	18	-0,4	708,3248	39,02	1,02E+10	1	13304	150211-Fr_I_Helo-red-alk-tryp-001.raw	37	37	AAC25688.1:O77463.1:AAC25689.1	Carbamidomethylation	C3:Carbamidomethylation:1000.00;C11:Carbamidomethylation:1000.00;C15:Carbamidomethylation:1000.00
**FFLNKDEASC(+57.02)VVSAC(+57.02)K**	79,89	1873,87	16	-2,3	937,9402	15,43	0	1	4935	150211-Fr_I_Helo-red-alk-tryp-001.raw	1	1	AIJ02112.2	Carbamidomethylation	C10:Carbamidomethylation:1000.00;C15:Carbamidomethylation:1000.00
**T(+59.02)QFGC(+57.02)PAYEGYC(+57.02)NDHC(+57.02)NDIER**	79,69	2664,025	21	1,4	889,0168	32,73	0	1	11015	150211-Fr_I_Helo-red-alk-tryp-001.raw	8	8	P0C2F3.1:P69940.2	Aminoethylcysteine; Carbamidomethylation	T1:Aminoethylcysteine:1000.00;C5:Carbamidomethylation:1000.00;C12:Carbamidomethylation:1000.00;C16:Carbamidomethylation:1000.00
**KTQFGC(+57.02)PAYEGYC(+57.02)NDHC(+57.02)NDIER**	78,94	2733,101	22	-2,2	912,0388	16,89	2,07E+09	1	5479	150211-Fr_I_Helo-red-alk-tryp-001.raw	4	4	P0C2F3.1:P69940.2	Carbamidomethylation	C6:Carbamidomethylation:1000.00;C13:Carbamidomethylation:1000.00;C17:Carbamidomethylation:1000.00
**KITC(+57.02)FLTAAGYC(+57.02)NTEC(+57.02)TLK**	78,32	2250,048	19	-0,6	1126,031	20,01	1,15E+07	1	6661	150211-Fr_I_Helo-red-alk-tryp-001.raw	3	3	AAC25688.1:O77463.1:AAC25689.1	Carbamidomethylation	C4:Carbamidomethylation:1000.00;C12:Carbamidomethylation:1000.00;C16:Carbamidomethylation:1000.00
**KQNEPSFIEDSAIPGHQQVLDPVDLVK**	78,23	3002,535	27	-2,3	751,6393	46,34	0	1	15935	150211-Fr_I_Helo-red-alk-tryp-001.raw	3	3	AIJ02110.2		
**DAC(+57.02)YC(+57.02)EHLPESVK**	78,2	1606,676	13	-0,5	804,3446	13,24	7,25E+07	1	4137	150211-Fr_I_Helo-red-alk-tryp-001.raw	41	41	JAW07006.1	Carbamidomethylation	C3:Carbamidomethylation:1000.00;C5:Carbamidomethylation:1000.00
**ESLIPGEEVFDFGHVVGNLNVLNC(+57.02)K**	77,98	2785,375	25	-0,1	697,3508	52,57	1,42E+08	1	18045	150211-Fr_I_Helo-red-alk-tryp-001.raw	19	19	AIJ02112.2	Carbamidomethylation	C24:Carbamidomethylation:1000.00
**QNEPSFIED(+21.98)SAIPGHQQVLDPVDLVK**	76,97	2896,422	26	-2,1	966,4792	46,21	3,18E+08	1	15887	150211-Fr_I_Helo-red-alk-tryp-001.raw	1	1	AIJ02110.2	Sodium adduct	D9:Sodium adduct:8.69
**EPDGSVSTNTLGVANLGGAC(+57.02)NPC(+57.02)LK**	76,93	2530,179	25	-1,3	844,3992	34,83	0	1	11775	150211-Fr_I_Helo-red-alk-tryp-001.raw	1	1	AIJ02112.2	Carbamidomethylation	C20:Carbamidomethylation:1000.00;C23:Carbamidomethylation:1000.00
**ADVDFTGIADSIIKK**	76,51	1591,846	15	-0,6	796,9297	36,15	5,88E+08	1	12249	150211-Fr_I_Helo-red-alk-tryp-001.raw	4	4	JAW07002.1		
**GSGINYLVLQVHYADVTR**	76,34	2004,043	18	-0,8	1003,028	37,03	0	1	12577	150211-Fr_I_Helo-red-alk-tryp-001.raw	1	1	XP_023239442.1		
**VTESDKQTILETHNQLR**	74,6	2011,033	17	0	671,3517	30,09	5,05E+07	1	10102	150211-Fr_I_Helo-red-alk-tryp-001.raw	33	33	JAW07031.1:JAW06995.1		
**DNLIPGPPAAFDPDVLISAMSK**	74,43	2267,151	22	0	756,7241	55,31	2,40E+08	1	18942	150211-Fr_I_Helo-red-alk-tryp-001.raw	2	2	AIJ02109.2:P86392.3:V9Z9A3.1:AHE40589.1:AHE40588.1		

**K(+43.01)(+14.02)LGELQDDGTVAYNTAGISLGSGVC(+57.02)K**	74,32	2709,328	26	-0,5	904,1161	36,21	6,06E+08	1	12273	150211-Fr_I_Helo-red-alk-tryp-001.raw	2	2	AIJ02110.2:JAG85213.1	Carbamylation; Methylation(KR); Carbamidomethylation	K1:Carbamylation:223.91;K1:Methylation(KR):223.91;C25:Carbamidomethylation:1000.00
**FGC(+57.02)PAYEGYC(+57.02)NDHC(+57.02)NDIER**	74,27	2375,899	19	-1,4	1188,955	14,47	0	1	4585	150211-Fr_I_Helo-red-alk-tryp-001.raw	1	1	P0C2F3.1:P69940.2	Carbamidomethylation	C3:Carbamidomethylation:1000.00;C10:Carbamidomethylation:1000.00;C14:Carbamidomethylation:1000.00
**ITC(+57.02)FLTAAGYC(+57.02)NTEC(+57.02)TLKK**	73,71	2250,048	19	-0,7	751,0228	18,32	2,83E+09	1	6020	150211-Fr_I_Helo-red-alk-tryp-001.raw	12	12	AAC25688.1:O77463.1:AAC25689.1	Carbamidomethylation	C3:Carbamidomethylation:1000.00;C11:Carbamidomethylation:1000.00;C15:Carbamidomethylation:1000.00
**LIGVQAFAINTEPSFIK**	73,5	1847,019	17	-1,3	924,5157	46,93	0	1	16137	150211-Fr_I_Helo-red-alk-tryp-001.raw	3	3	AIJ02111.3		
**WADEVLNLDDFETIR**	72,64	1834,874	15	0	918,4441	46,86	0	1	16112	150211-Fr_I_Helo-red-alk-tryp-001.raw	2	2	JAW07036.1:JAW06986.1		
**VVNPMVSIFTNVDITEVTR**	72,48	2133,114	19	-1,5	712,0442	51,11	0	1	17561	150211-Fr_I_Helo-red-alk-tryp-001.raw	2	2	JAW07019.1:JAW06978.1		
**TGGFPVNS(+340.09)NNC(+57.02)IYPC(+57.02)YSTQDEIQC(+57.02)EEFC(+57.02)EK**	72,3	3985,565	30	-0,6	997,3979	44,4	0	1	15248	150211-Fr_I_Helo-red-alk-tryp-001.raw	1	1	JAW07006.1	Phosphopantetheine; Carbamidomethylation	S8:Phosphopantetheine:31.37;C11:Carbamidomethylation:1000.00;C15:Carbamidomethylation:1000.00;C24:Carbamidomethylation:1000.00;C28:Carbamidomethylation:1000.00
**LNTNIGGVANYASVC(+57.02)HPC(+57.02)YK**	71,58	2237,036	20	0,7	746,6863	17,59	0	1	5742	150211-Fr_I_Helo-red-alk-tryp-001.raw	1	1	AIJ02113.2	Carbamidomethylation	C15:Carbamidomethylation:1000.00;C18:Carbamidomethylation:1000.00
**DYLLYC(+57.02)DFSDKDPK**	71,45	1777,787	14	-1,8	889,8991	34,55	0	1	11678	150211-Fr_I_Helo-red-alk-tryp-001.raw	42	42	JAW07031.1:JAW06995.1:JAG85161.1	Carbamidomethylation	C6:Carbamidomethylation:1000.00
**EALAPEPAPEPAPEPAPEA**	71,38	1881,9	19	-0,9	941,9562	18,56	8,13E+08	1	6113	150211-Fr_I_Helo-red-alk-tryp-001.raw	41	41	JAW07022.1:P86821.1		
**LATDDQC(+57.02)IVIEILSVTDKLVTK**	71,38	2473,335	22	0,7	825,4529	55,38	0	1	18964	150211-Fr_I_Helo-red-alk-tryp-001.raw	1	1	AIJ02113.2	Carbamidomethylation	C7:Carbamidomethylation:1000.00
**T(+43.01)EPSFIK(+14.02)DNLIPGPPAAFDPDVLISAMSK**	70,92	3126,595	29	-0,1	1043,205	57,08	7,10E+07	1	19527	150211-Fr_I_Helo-red-alk-tryp-001.raw	1	1	AIJ02109.2:P86392.3:V9Z9A3.1:AHE40589.1:AHE40588.1	Carbamylation; Methylation(KR)	T1:Carbamylation:115.16;K7:Methylation(KR):129.58
**GGPNSDGSYDHGLFQINDR**	70,86	2047,898	19	-2	683,6387	32,91	0	1	11080	150211-Fr_I_Helo-red-alk-tryp-001.raw	1	1	XP_023218922.1		
**S(+114.04)SGYC(+57.02)AWPAC(+57.02)YC(+57.02)YGLPDSVK**	70,78	2454,008	20	-0,3	1228,011	43,32	2,08E+09	1	14860	150211-Fr_I_Helo-red-alk-tryp-001.raw	12	12	AAC25688.1:O77463.1:AAC25689.1	Ubiquitin; Carbamidomethylation	S1:Ubiquitin:11.06;C5:Carbamidomethylation:1000.00;C10:Carbamidomethylation:1000.00;C12:Carbamidomethylation:1000.00
**NVLVIAPPDPSVFSDPDRDIELAK**	70,68	2606,359	24	-0,6	869,7932	48,74	8,30E+08	1	16758	150211-Fr_I_Helo-red-alk-tryp-001.raw	1	1	AIJ02118.2		
**ITC(+57.02)FLTAAGYC(+57.02)NTE(+21.98)C(+57.02)TLK**	70,52	2143,935	18	-0,3	1072,975	36,07	1,07E+09	1	12221	150211-Fr_I_Helo-red-alk-tryp-001.raw	9	9	AAC25688.1:O77463.1:AAC25689.1	Carbamidomethylation; Sodium adduct	C3:Carbamidomethylation:1000.00;C11:Carbamidomethylation:1000.00;E14:Sodium

															adduct:55.21;C15:Carbamidomethylation:1000.00
**C(+57.02)AWPAC(+57.02)YC(+57.02)YGVPDHIK**	69,89	1995,843	16	0,7	666,2888	34,06	0	1	11499	150211-Fr_I_Helo-red-alk-tryp-001.raw	2	2	JAW07016.1:JAW06979.1:P68411.1:1913435A:P68410.1:AAB36322.1	Carbamidomethylation	C1:Carbamidomethylation:1000.00;C6:Carbamidomethylation:1000.00;C8:Carbamidomethylation:1000.00
**FATDEALTQHMTLTYVK**	69,74	1967,966	17	-0,4	656,9958	34,59	0	1	11689	150211-Fr_I_Helo-red-alk-tryp-001.raw	1	1	AIJ02113.2		
**ETAVTLAHESAHLLGSPHDGEGDYFSLPGSPGSTK**	69,58	3563,68	35	0,3	713,7436	38,03	1,83E+09	1	12949	150211-Fr_I_Helo-red-alk-tryp-001.raw	1	1	AMO02558.1:JAW07025.1:JAW06984.1		
**GSSGYC(+57.02)AWPAC(+57.02)YC(+57.02)YGLPNWVK**	69,48	2495,05	21	-0,9	832,6897	48,83	0	1	16789	150211-Fr_I_Helo-red-alk-tryp-001.raw	1	1	AAW72453.1:Q5G8B8.1:AAB29128.1:P56612.1:AIA81145.1:CAA46982.1:P15226.2:P0DMM8.1:1NPI:1B7D:AAB36321.1:AAB36318.1:P56611.1:JAG85191.1:JAG85210.1	Carbamidomethylation	C6:Carbamidomethylation:1000.00;C11:Carbamidomethylation:1000.00;C13:Carbamidomethylation:1000.00
**SLFVGEDVFNMELIAK**	69,45	1810,918	16	0,1	604,6465	50,35	1,38E+08	1	17307	150211-Fr_I_Helo-red-alk-tryp-001.raw	20	20	AMO02558.1:JAW07025.1		
**AD(-18.01)VDFTGIADSIIK**	69,16	1445,74	14	-0,4	723,877	45,96	0	1	15800	150211-Fr_I_Helo-red-alk-tryp-001.raw	2	2	JAW07002.1:P86824.1	Dehydration	D2:Dehydration:33.18
**AEIDFSGIPE(+21.98)DIIK**	68,9	1567,775	14	-1,2	784,8936	39,48	6,22E+08	1	13472	150211-Fr_I_Helo-red-alk-tryp-001.raw	119	119	JAW06987.1:JAW07038.1:JAW06992.1:P84190.1	Sodium adduct	E10:Sodium adduct:22.65
**SSGYC(+57.02)AWPAC(+57.02)YC(+57.02)YGVPDHIK**	68,7	2389,992	20	-0,3	797,6709	36,57	1,02E+08	1	12403	150211-Fr_I_Helo-red-alk-tryp-001.raw	1	1	JAW07016.1:JAW06979.1:P68411.1:1913435A:P68410.1:AAB36322.1	Carbamidomethylation	C5:Carbamidomethylation:1000.00;C10:Carbamidomethylation:1000.00;C12:Carbamidomethylation:1000.00
**SDVTGGFPVNS(+58.03)NNC(+57.02)IYPC(+57.02)YSTQDEIQC(+57.02)EEFC(+57.02)EK**	68,51	4004,636	33	4,3	1335,892	44,62	0	1	15326	150211-Fr_I_Helo-red-alk-tryp-001.raw	1	1	JAW07006.1	2 3-dihydro-2 2-dimethyl-7-benzofuranol N-methyl carbamate; Carbamidomethylation	S11:2 3-dihydro-2 2-dimethyl-7-benzofuranol N-methyl carbamate:2.53;C14:Carbamidomethylation:1000.00;C18:Carbamidomethylation:1000.00;C27:Carbamidomethylation:1000.00;C31:Carbamidomethylation:1000.00
**NAGNENSVIEILVINPEVVK**	68,35	2150,158	20	-0,8	717,7261	46,63	0	1	16035	150211-Fr_I_Helo-red-alk-tryp-001.raw	2	2	AIJ02118.2		
**TQ(+.98)FGC(+57.02)PAYEGYC(+57.02)NDHC(+57.02)NDIER**	68,27	2605,99	21	4,4	1304,008	15,44	0	1	4937	150211-Fr_I_Helo-red-alk-tryp-001.raw	6	6	P0C2F3.1:P69940.2	Deamidation (NQ); Carbamidomethylation	Q2:Deamidation (NQ):108.57;C5:Carbamidomethylation:1000.00;C12:Carbamidomethylation:1000.00;C16:Carbamidomethylation:1000.00
**ADVDFTGIAD(-18.01)SIIK**	68,03	1445,74	14	-0,4	723,877	37,32	0	1	12682	150211-Fr_I_Helo-red-alk-tryp-001.raw	1	1	JAW07002.1:P86824.1	Dehydration	D10:Dehydration:0.00
**A(+27.99)EIDFSGIPEDIIK**	67,88	1573,788	14	-0,4	787,9007	46,97	1,16E+08	1	16151	150211-Fr_I_Helo-red-alk-tryp-001.raw	6	6	P84190.1	Formylation (Protein N-term)	A1:Formylation (Protein N-term):1000.00

**A(+27.99)EIDFSGIPEDIIK**	67,88	1573,788	14	-0,4	787,9007	46,97	1,16E+08	1	16151	150211-Fr_I_Helo-red-alk-tryp-001.raw	6	6	JAW06987.1:JAW07038.1:JAW06992.1	Formylation	A1:Formylation:111.11
**AEIDFS(-18.01)GIPEDIIK**	67,83	1527,782	14	-0,4	764,8979	44,63	0	1	15330	150211-Fr_I_Helo-red-alk-tryp-001.raw	18	18	JAW06987.1:JAW07038.1:JAW06992.1:P84190.1	Dehydration	S6:Dehydration:32.28
**QIGIFDSKGTILVGVLGVANLGR**	67,78	2326,337	23	-0,9	776,4523	51,34	0	1	17638	150211-Fr_I_Helo-red-alk-tryp-001.raw	2	2	AMO02558.1:JAW07025.1:JAW06984.1		
**LTEADIILLFSQR**	67,53	1517,845	13	-1,4	759,9289	47,19	0	1	16231	150211-Fr_I_Helo-red-alk-tryp-001.raw	2	2			
**LTS(+58.03)QSEYAC(+57.02)PAIDKFC(+57.02)EDHC(+57.02)AAK**	67,5	2758,191	23	-1,9	920,4026	23,17	0	1	7822	150211-Fr_I_Helo-red-alk-tryp-001.raw	22	22	P86822.2:P0C8W4.2:AFP87551.1	2 3-dihydro-2 2-dimethyl-7-benzofuranol N-methyl carbamate; Carbamidomethylation	S3:2 3-dihydro-2 2-dimethyl-7-benzofuranol N-methyl carbamate:14.04;C9:Carbamidomethylation:1000.00;C16:Carbamidomethylation:1000.00;C20:Carbamidomethylation:1000.00
**VGVAQDDSDYNER**	67,46	1466,627	13	-0,7	734,3204	6,38	0	1	1664	150211-Fr_I_Helo-red-alk-tryp-001.raw	32	32	AIJ02110.2:JAG85213.1		
**AEID(+21.98)FSGIPEDIIK**	67,25	1567,775	14	0,3	523,5989	38,32	4,27E+06	1	13054	150211-Fr_I_Helo-red-alk-tryp-001.raw	41	41	JAW06987.1:JAW07038.1:JAW06992.1:P84190.1	Sodium adduct	D4:Sodium adduct:48.33
**ADVDFTGIADSIIK**	67,24	1463,751	14	-0,3	732,8824	42,74	2,72E+10	1	14653	150211-Fr_I_Helo-red-alk-tryp-001.raw	288	288	JAW07002.1:P86824.1		
**LTSQSEYAC(+57.02)PAIDKFC(+57.02)EDHC(+57.02)AA**	67,16	2572,067	22	0,3	858,3632	17,5	1,08E+09	1	5708	150211-Fr_I_Helo-red-alk-tryp-001.raw	7	7	P86822.2:P0C8W4.2:AFP87551.1	Carbamidomethylation	C9:Carbamidomethylation:1000.00;C16:Carbamidomethylation:1000.00;C20:Carbamidomethylation:1000.00
**LSSAANMMQ(+.98)MEWDDELAAVAQAHANQC(+57.02)K**	67	3120,341	28	5,1	1041,126	47,71	0	1	16411	150211-Fr_I_Helo-red-alk-tryp-001.raw	1	1	JAW07031.1:JAW06995.1	Deamidation (NQ); Carbamidomethylation	Q9:Deamidation (NQ):0.00;C27:Carbamidomethylation:1000.00
**LATDDQC(+57.02)IVIEILSVTDK**	66,85	2032,04	18	-1,2	678,3531	48,38	0	1	16639	150211-Fr_I_Helo-red-alk-tryp-001.raw	1	1	AIJ02113.2	Carbamidomethylation	C7:Carbamidomethylation:1000.00
**ADVD(+21.98)FTGIADSIIK**	66,62	1485,733	14	1,2	496,2521	38,01	0	1	12941	150211-Fr_I_Helo-red-alk-tryp-001.raw	1	1	JAW07002.1:P86824.1	Sodium adduct	D4:Sodium adduct:23.04
**SHTFC(+57.02)TPSTC(+57.02)K**	66,61	1324,554	11	-0,5	663,2839	5,76	3,93E+07	1	1443	150211-Fr_I_Helo-red-alk-tryp-001.raw	22	22	JAW07031.1:JAW06995.1:JAG85161.1	Carbamidomethylation	C5:Carbamidomethylation:1000.00;C10:Carbamidomethylation:1000.00
**C(+57.02)AWPAC(+57.02)YC(+57.02)YGLPDSVK**	66,49	1945,816	16	-1,2	973,9141	37,34	0	1	12692	150211-Fr_I_Helo-red-alk-tryp-001.raw	4	4	AAC25688.1:O77463.1:AAC25689.1	Carbamidomethylation	C1:Carbamidomethylation:1000.00;C6:Carbamidomethylation:1000.00;C8:Carbamidomethylation:1000.00
**ADVDFTGIAD(+21.98)SIIK**	66,48	1485,733	14	0,9	743,8743	37,95	0	1	12915	150211-Fr_I_Helo-red-alk-tryp-001.raw	2	2	JAW07002.1:P86824.1	Sodium adduct	D10:Sodium adduct:49.80
**AEIDFSGIPEDIIK**	66,41	1545,793	14	-0,6	773,9031	43,43	6,01E+10	1	14899	150211-Fr_I_Helo-red-alk-tryp-001.raw	371	371	JAW06987.1:JAW07038.1:JAW06992.1:P84190.1		
**DSWTQ(+.98)LYVC(+57.02)NYGPAGNLDDSELYKVDKPC(+57.02)EK**	66,23	3664,634	31	4,7	1222,558	45,28	0	1	15560	150211-Fr_I_Helo-red-alk-tryp-001.raw	1	1	JAW06991.1:JAW07032.1	Deamidation (NQ); Carbamidomethylation	Q5:Deamidation (NQ):76.17;C9:Carbamidomet

															hylation:1000.00;C29:Carbamidomethylation:1000.00
**EALAPEPAPE(+21.98)PAPEPAPEA**	66,11	1903,882	19	-3,3	952,9448	18,8	0	1	6206	150211-Fr_I_Helo-red-alk-tryp-001.raw	9	9	JAW07022.1:P86821.1	Sodium adduct	E10:Sodium adduct:16.65
**QNEPS(+58.03)FIEDSAIPGHQQVLDPVDLVK**	66	2932,469	26	-1,6	978,4954	46,36	0	1	15941	150211-Fr_I_Helo-red-alk-tryp-001.raw	6	6	AIJ02110.2	2 3-dihydro-2 2-dimethyl-7-benzofuranol N-methyl carbamate	S5:2 3-dihydro-2 2-dimethyl-7-benzofuranol N-methyl carbamate:61.30
**ES(+58.03)LIPGEEVFDFGHVVGNLNVLNC(+57.02)K**	65,96	2843,404	25	-2,2	948,8065	52,93	0	1	18163	150211-Fr_I_Helo-red-alk-tryp-001.raw	3	3	AIJ02112.2	2 3-dihydro-2 2-dimethyl-7-benzofuranol N-methyl carbamate; Carbamidomethylation	S2:2 3-dihydro-2 2-dimethyl-7-benzofuranol N-methyl carbamate:1000.00;C24:Carbamidomethylation:1000.00
**FGPNVAGET(+59.02)AASEVNFHFSAPHLIFPEGMK**	65,89	3259,558	30	1,8	815,8983	52,12	0	1	17895	150211-Fr_I_Helo-red-alk-tryp-001.raw	4	4	JAW07031.1:JAW06995.1	Aminoethylcysteine	T9:Aminoethylcysteine:47.09
**C(+57.02)IVVEFLC(+57.02)VTESK**	65,79	1582,773	13	1	528,5989	37,35	0	1	12695	150211-Fr_I_Helo-red-alk-tryp-001.raw	3	3	JAW07039.1:JAG85190.1	Carbamidomethylation	C1:Carbamidomethylation:1000.00;C8:Carbamidomethylation:1000.00
**LTSQ(+.98)SEYAC(+57.02)PAIDKFC(+57.02)EDHC(+57.02)AAK**	65,5	2701,146	23	5,1	901,3938	17,99	0	1	5895	150211-Fr_I_Helo-red-alk-tryp-001.raw	4	4	P86822.2:P0C8W4.2:AFP87551.1	Deamidation (NQ); Carbamidomethylation	Q4:Deamidation (NQ):1000.00;C9:Carbamidomethylation:1000.00;C16:Carbamidomethylation:1000.00;C20:Carbamidomethylation:1000.00
**ENSWVTDVALHQVFK**	65,43	1771,889	15	-1,9	886,9503	36,66	0	1	12439	150211-Fr_I_Helo-red-alk-tryp-001.raw	1	1			
**GMGDPREDGTVDIN(+.98)TAGIANSAGVC(+57.02)KPC(+57.02)FK**	65,39	3137,422	30	3,6	1046,818	35,87	0	1	12150	150211-Fr_I_Helo-red-alk-tryp-001.raw	1	1	AIJ02109.2:P86392.3:V9Z9A3.1:AHE40589.1:AHE40588.1	Deamidation (NQ); Carbamidomethylation	N14:Deamidation (NQ):49.80;C25:Carbamidomethylation:1000.00;C28:Carbamidomethylation:1000.00
**FGPNVAGETAASEVNFHFSAPHLIFPEGM(+15.99)K(-.98)(+42.01)**	65,22	3257,56	30	0,5	815,3978	51,08	2,29E+09	1	17551	150211-Fr_I_Helo-red-alk-tryp-001.raw	3	3	JAW07031.1:JAW06995.1	Oxidation (M); Amidation; Acetylation (K)	M29:Oxidation (M):1000.00;K30:Amidation:1000.00;K30:Acetylation (K):1000.00
**S(+28.03)GAYFWPASDIPIK**	65,21	1578,808	14	-0,9	790,4106	46,22	0	1	15891	150211-Fr_I_Helo-red-alk-tryp-001.raw	5	5	XP_023242894.1	Ethylation	S1:Ethylation:99.51
**C(+57.02)VEHLLSLPR**	65,19	1222,649	10	-0,1	408,5569	31,56	0	1	10601	150211-Fr_I_Helo-red-alk-tryp-001.raw	4	4	AIJ02110.2:JAG85213.1	Carbamidomethylation	C1:Carbamidomethylation:1000.00
**AEIDFSGIPED(-18.01)IIK**	65,19	1527,782	14	0,1	764,8983	45,55	2,80E+08	1	15654	150211-Fr_I_Helo-red-alk-tryp-001.raw	27	27	JAW06987.1:JAW07038.1:JAW06992.1:P84190.1	Dehydration	D11:Dehydration:51.47
**A(+27.99)DVDFTGIADSIIK**	65,13	1491,746	14	-1,2	746,8792	46,68	0	1	16052	150211-Fr_I_Helo-red-alk-tryp-001.raw	1	1	P86824.1	Formylation (Protein N-term)	A1:Formylation (Protein N-term):1000.00
**A(+27.99)DVDFTGIADSIIK**	65,13	1491,746	14	-1,2	746,8792	46,68	0	1	16052	150211-Fr_I_Helo-red-alk-tryp-001.raw	1	1	JAW07002.1	Formylation	A1:Formylation:89.58
**LLGNREPDGS(+59.02)VSTNTLGVANLGGAC(+57.02)NPC(+57.02)LK**	65,01	3142,532	30	-0,4	1048,518	37,02	0	1	12575	150211-Fr_I_Helo-red-alk-tryp-001.raw	1	1	AIJ02112.2	Aminoethylcysteine; Carbamidomethylation	S10:Aminoethylcysteine:6.59;C25:Carbamidomethylation:1000.00;C28:Carbamidomethylation:1000.00

**AEIDFSGIP(+31.99)EDIIK**	64,66	1577,782	14	-1,1	789,8976	38,48	0	1	13110	150211-Fr_I_Helo-red-alk-tryp-001.raw	8	8	JAW06987.1:JAW07038.1:JAW06992.1:P84190.1	Dihydroxy	P9:Dihydroxy:28.02
**LIGVQAFAINTE(+21.98)PSFIK**	64,62	1869,001	17	1,2	935,509	46,84	0	1	16105	150211-Fr_I_Helo-red-alk-tryp-001.raw	1	1	AIJ02111.3	Sodium adduct	E12:Sodium adduct:47.64
**ITC(+57.02)FLTAAGYC(+57.02)NTE(+37.96)C(+57.02)TLK**	64,4	2159,909	18	-1,4	1080,96	34,89	0	1	11796	150211-Fr_I_Helo-red-alk-tryp-001.raw	3	3	AAC25688.1:O77463.1:AAC25689.1	Carbamidomethylation; Replacement of proton by potassium	C3:Carbamidomethylation:1000.00;C11:Carbamidomethylation:1000.00;E14:Replacement of proton by potassium:46.21;C15:Carbamidomethylation:1000.00
**AEIDFSGIPEDIIKQIK**	64,38	1915,03	17	-0,3	639,3505	46,59	3,17E+08	1	16021	150211-Fr_I_Helo-red-alk-tryp-001.raw	8	8	JAW06987.1:JAW07038.1:JAW06992.1		
**EDGTVDIN(+.98)TAGIANSAGVC(+57.02)KPC(+57.02)FK**	64,34	2524,157	24	2,7	842,3953	35,03	0	1	11848	150211-Fr_I_Helo-red-alk-tryp-001.raw	2	2	AIJ02109.2:P86392.3:V9Z9A3.1:AHE40589.1:AHE40588.1	Deamidation (NQ); Carbamidomethylation	N8:Deamidation (NQ):72.73;C19:Carbamidomethylation:1000.00;C22:Carbamidomethylation:1000.00
**EALAPEPAPEPAPEPAPEAAPEA**	63,72	2250,069	23	-1,8	1126,04	35,78	0	1	12115	150211-Fr_I_Helo-red-alk-tryp-001.raw	1	1	JAW07022.1:P86821.1		
**AEIDFSGIPE(+21.98)DIIKQ**	63,53	1695,833	15	-2,1	848,9221	42,31	0	1	14499	150211-Fr_I_Helo-red-alk-tryp-001.raw	6	6	JAW06987.1:JAW07038.1:JAW06992.1	Sodium adduct	E10:Sodium adduct:15.10
**SEYAC(+57.02)PAIDKFC(+57.02)EDHC(+57.02)AA**	63,23	2142,845	18	1,6	1072,431	15,86	0	1	5093	150211-Fr_I_Helo-red-alk-tryp-001.raw	4	4	P86822.2:P0C8W4.2:AFP87551.1:Q0GY42.1:ABE98265.1:JAG85223.1	Carbamidomethylation	C5:Carbamidomethylation:1000.00;C12:Carbamidomethylation:1000.00;C16:Carbamidomethylation:1000.00
**SVYDIEANPILLK**	63,21	1473,808	13	0,6	737,9116	36,17	0	1	12257	150211-Fr_I_Helo-red-alk-tryp-001.raw	1	1	AIJ02118.2		
**KGS(+58.03)SGYC(+57.02)AWPAC(+57.02)YC(+57.02)YGLPDSVK**	62,81	2583,111	22	-1,7	1292,56	37,66	0	1	12807	150211-Fr_I_Helo-red-alk-tryp-001.raw	9	9	AAC25688.1:O77463.1:AAC25689.1	2 3-dihydro-2 2-dimethyl-7-benzofuranol N-methyl carbamate; Carbamidomethylation	S3:2 3-dihydro-2 2-dimethyl-7-benzofuranol N-methyl carbamate:0.00;C7:Carbamidomethylation:1000.00;C12:Carbamidomethylation:1000.00;C14:Carbamidomethylation:1000.00
**KEALAPEPAPEPAPEPAPEA**	62,72	2009,995	20	-1	1006,004	18,8	5,06E+09	1	6203	150211-Fr_I_Helo-red-alk-tryp-001.raw	79	79	JAW07022.1:P86821.1		
**GIFDIADEILPITTNFFR**	62,65	2081,083	18	-0,4	694,7014	61,18	6,52E+06	1	20835	150211-Fr_I_Helo-red-alk-tryp-001.raw	3	3	JAW07023.1		
**AWPAC(+57.02)YC(+57.02)YGLPDSVK**	62,65	1785,785	15	-1,2	893,8989	36,71	0	1	12456	150211-Fr_I_Helo-red-alk-tryp-001.raw	3	3	AAC25688.1:O77463.1:AAC25689.1	Carbamidomethylation	C5:Carbamidomethylation:1000.00;C7:Carbamidomethylation:1000.00
**AEIDFSGIPE(+37.96)DIIK**	62,6	1583,748	14	-3,2	792,879	38,29	0	1	13043	150211-Fr_I_Helo-red-alk-tryp-001.raw	29	29	JAW06987.1:JAW07038.1:JAW06992.1:P84190.1	Replacement of proton by potassium	E10:Replacement of proton by potassium:40.57
**KQNEPSFIEDSAIPGHQ(+.98)QVLDPVDLVK**	62,57	3003,519	27	4,5	1002,185	41,71	0	1	14282	150211-Fr_I_Helo-red-alk-tryp-001.raw	1	1	AIJ02110.2	Deamidation (NQ)	Q17:Deamidation (NQ):0.00
**LTSQSEYAC(+57.02)PAIDK**	62,56	1581,735	14	-1,1	791,8737	30,48	3,37E+08	1	10232	150211-Fr_I_Helo-red-alk-tryp-001.raw	60	60	P86822.2:P0C8W4.2:AFP87551.1	Carbamidomethylation	C9:Carbamidomethylation:1000.00

**DGYFQDPDDC(+57.02)QK**	62,56	1486,567	12	-2,2	744,2891	30,43	0	1	10215	150211-Fr_I_Helo-red-alk-tryp-001.raw	5	5		Carbamidomethylation	C10:Carbamidomethylation:1000.00
**VGC(+57.02)GVAGYVENGVK**	62,28	1407,682	14	0	704,8481	30,73	0	1	10317	150211-Fr_I_Helo-red-alk-tryp-001.raw	16	16	JAW07031.1:JAW06995.1:JAG85161.1	Carbamidomethylation	C3:Carbamidomethylation:1000.00
**GTILVGVLGVANLGR**	62,17	1437,867	15	1,8	480,297	37,94	0	1	12914	150211-Fr_I_Helo-red-alk-tryp-001.raw	2	2	AMO02558.1:JAW07025.1:JAW06984.1		
**KEALAPEPAPEPAPEPAPEAAPEAAPEPAAAAPE**	62,09	3282,593	34	0,6	1095,206	37,62	5,41E+09	1	12793	150211-Fr_I_Helo-red-alk-tryp-001.raw	3	3	JAW07022.1:P86821.1		
**WWGGEPIWVTAR**	61,86	1456,725	12	1	729,3705	39,96	0	1	13645	150211-Fr_I_Helo-red-alk-tryp-001.raw	24	24	XP_023242894.1		
**DADIIMLISNR**	61,35	1259,654	11	-0,4	630,8342	35,29	0	1	11939	150211-Fr_I_Helo-red-alk-tryp-001.raw	11	11	AIJ02110.2:JAG85213.1		
**FGC(+57.02)PAYEGYC(+57.02)N(+.98)DHC(+57.02)NDIER**	61,26	2376,883	19	4,7	1189,455	14,25	0	1	4503	150211-Fr_I_Helo-red-alk-tryp-001.raw	2	2	P0C2F3.1:P69940.2	Carbamidomethylation; Deamidation (NQ)	C3:Carbamidomethylation:1000.00;C10:Carbamidomethylation:1000.00;N11:Deamidation (NQ):38.03;C14:Carbamidomethylation:1000.00
**VGC(+57.02)GVAGYVENGVKK**	61,16	1535,777	15	0,1	512,9329	30,28	0	1	10164	150211-Fr_I_Helo-red-alk-tryp-001.raw	4	4	JAW07031.1:JAW06995.1	Carbamidomethylation	C3:Carbamidomethylation:1000.00
**VGVSLDETDYNER**	61,1	1495,679	13	-0,2	748,8467	31,05	0	1	10428	150211-Fr_I_Helo-red-alk-tryp-001.raw	13	13	JAW07039.1:JAG85190.1:JAW07042.1		
**EALAPEPAPE(+37.96)PAPEPAPEA**	60,78	1919,855	19	-2,5	960,9326	18,89	0	1	6239	150211-Fr_I_Helo-red-alk-tryp-001.raw	3	3	JAW07022.1:P86821.1	Replacement of proton by potassium	E10:Replacement of proton by potassium:32.11
**AIITVVDNGIMIGC(+57.02)GIFDVASPSVK**	60,62	2575,339	25	-1,2	859,4525	53,4	0	1	18316	150211-Fr_I_Helo-red-alk-tryp-001.raw	1	1	JAW07019.1:JAW06978.1	Carbamidomethylation	C14:Carbamidomethylation:1000.00
**KEALAPEPAPEPAPEPAPEAAPEA**	60,6	2378,164	24	-0,6	1190,089	35,12	0	1	11877	150211-Fr_I_Helo-red-alk-tryp-001.raw	4	4	JAW07022.1:P86821.1		
**KEALAPEPAPEPAPEPAPEAAPEAAPEPAA**	60,49	2914,423	30	-1	1458,218	36,5	6,23E+08	1	12377	150211-Fr_I_Helo-red-alk-tryp-001.raw	1	1	JAW07022.1:P86821.1		
**FGPN(+.98)VAGETAASEVNFHFSAPHLIFPEGM(+15.99)K**	60,42	3217,518	30	3,6	805,3896	49,32	0	1	16959	150211-Fr_I_Helo-red-alk-tryp-001.raw	1	1	JAW07031.1:JAW06995.1	Deamidation (NQ); Oxidation (M)	N4:Deamidation (NQ):67.73;M29:Oxidation (M):1000.00
**AEIDFSGIPED(+21.98)IIKQ**	60,35	1695,833	15	-1,1	848,9229	42,83	0	1	14684	150211-Fr_I_Helo-red-alk-tryp-001.raw	3	3	JAW06987.1:JAW07038.1:JAW06992.1	Sodium adduct	D11:Sodium adduct:0.00
**ANAEYLLSLTK**	60,28	1221,661	11	-0,1	611,8375	33,32	0	1	11228	150211-Fr_I_Helo-red-alk-tryp-001.raw	6	6	AIJ02113.2		
**GS(+58.03)SGYC(+57.02)AWPAC(+57.02)YC(+57.02)YGLPDSVK**	60,2	2455,016	21	-2,3	819,3439	43,54	0	1	14939	150211-Fr_I_Helo-red-alk-tryp-001.raw	43	43	AAC25688.1:O77463.1:AAC25689.1	2 3-dihydro-2 2-dimethyl-7-benzofuranol N-methyl carbamate; Carbamidomethylation	S2:2 3-dihydro-2 2-dimethyl-7-benzofuranol N-methyl carbamate:0.00;C6:Carbamidomethylation:1000.00;C11:Carbamidomethylation:1000.00;C13:Carbamidomethylation:1000.00
**IAFLPYGYILDKWR**	59,93	1753,955	14	-0,8	585,6586	49,94	0	1	17170	150211-Fr_I_Helo-red-alk-tryp-001.raw	1	1			
**YAC(+57.02)PAIDKFC(+57.02)EDHC(+57.02)AAK**	59,87	2054,865	17	0,5	514,7237	31,29	0	1	10510	150211-Fr_I_Helo-red-alk-tryp-001.raw	1	1	P86822.2:P0C8W4.2:AFP87551.1:Q0GY4	Carbamidomethylation	C3:Carbamidomethylation:1000.00;C10:Carbamidomethylati

													2.1:ABE98265.1:JAG 85223.1		on:1000.00;C14:Carbamidomethylation:1000.00
**E(+226.08)PSFIEDSAIPGHQQVLDPVDLVK**	59,83	2858,416	24	0,2	1430,216	48,71	0	1	16749	150211-Fr_I_Helo-red-alk-tryp-001.raw	2	2	AIJ02110.2	Biotinylation	E1:Biotinylation:165.29
**DC(+57.02)PESDGYIMGSGNNK**	59,83	1742,688	16	-0,2	872,3509	30,54	0	1	10254	150211-Fr_I_Helo-red-alk-tryp-001.raw	1	1	AIJ02110.2	Carbamidomethylation	C2:Carbamidomethylation:1000.00
**YANLGEFPWMVFIR**	59,82	1741,865	14	-1,6	871,9384	56,69	0	1	19398	150211-Fr_I_Helo-red-alk-tryp-001.raw	2	2	JAG85164.1		
**APEPAPEAAPEAAPEPAAAAPE**	59,69	2052,964	22	0,3	1027,49	17,04	3,08E+09	1	5532	150211-Fr_I_Helo-red-alk-tryp-001.raw	27	27	JAW07022.1:P86821.1		
**AEIDFSGIPEDIIK(+114.04)QIK**	59,51	2029,073	17	-0,9	677,3644	47,36	3,74E+08	1	16290	150211-Fr_I_Helo-red-alk-tryp-001.raw	1	1	JAW06987.1:JAW07038.1:JAW06992.1	Ubiquitin	K14:Ubiquitin:12.16
**YFFTLPQASC(+57.02)LR**	59,43	1501,739	12	0,4	751,877	38,7	0	1	13190	150211-Fr_I_Helo-red-alk-tryp-001.raw	7	7	JAW07039.1	Carbamidomethylation	C10:Carbamidomethylation:1000.00
**KNVLVIAPPDPSVFSDPDRDIELAK**	59,33	2734,454	25	-0,6	684,6204	46,58	3,02E+08	1	16017	150211-Fr_I_Helo-red-alk-tryp-001.raw	1	1	AIJ02118.2		
**DVDFTGIADSIIK**	59,05	1392,714	13	-0,5	697,3638	41,74	0	1	14292	150211-Fr_I_Helo-red-alk-tryp-001.raw	19	19	JAW07002.1:P86824.1		
**A(+43.01)EIDFSGIPEDIIK**	59	1588,798	14	-0,9	795,4058	46,5	0	1	15989	150211-Fr_I_Helo-red-alk-tryp-001.raw	1	1	JAW06987.1:JAW07038.1:JAW06992.1:P84190.1	Carbamylation	A1:Carbamylation:98.05
**FC(+57.02)EDHC(+57.02)AAK**	58,91	1136,438	9	-0,1	569,2261	5,6	0	1	1389	150211-Fr_I_Helo-red-alk-tryp-001.raw	69	69	P86822.2:P0C8W4.2:AFP87551.1:Q0GY42.1:ABE98265.1:JAG85223.1:ABV64385.1:ABE98262.1:Q0GY45.1:Q0GY43.1:ABE98264.1	Carbamidomethylation	C2:Carbamidomethylation:1000.00;C6:Carbamidomethylation:1000.00
**IRPAGFC(+57.02)DGYC(+57.02)K**	58,88	1442,643	12	0,1	481,8885	30,09	0	1	10104	150211-Fr_I_Helo-red-alk-tryp-001.raw	7	7	JAW07016.1:JAW06979.1:P68411.1:1913435A:P68410.1:AAB36322.1	Carbamidomethylation	C7:Carbamidomethylation:1000.00;C11:Carbamidomethylation:1000.00
**SSGYC(+57.02)AWPAC(+57.02)YC(+57.02)YGLPDSVK**	58,73	2339,965	20	-1,1	1170,988	43,38	0	1	14882	150211-Fr_I_Helo-red-alk-tryp-001.raw	2	2	AAC25688.1:O77463.1:AAC25689.1	Carbamidomethylation	C5:Carbamidomethylation:1000.00;C10:Carbamidomethylation:1000.00;C12:Carbamidomethylation:1000.00
**KTQ(+.98)FGC(+57.02)PAYEGYC(+57.02)NDHC(+57.02)NDIER**	58,71	2734,085	22	3,5	912,372	19,95	0	1	6641	150211-Fr_I_Helo-red-alk-tryp-001.raw	13	13	P0C2F3.1:P69940.2	Deamidation (NQ); Carbamidomethylation	Q3:Deamidation (NQ):108.57;C6:Carbamidomethylation:1000.00;C13:Carbamidomethylation:1000.00;C17:Carbamidomethylation:1000.00
**APEAAPEAAPE(+21.98)PAAAAPE**	58,57	1680,761	18	-3,4	841,3848	13,58	0	1	4259	150211-Fr_I_Helo-red-alk-tryp-001.raw	3	3	JAW07022.1:P86821.1	Sodium adduct	E11:Sodium adduct:55.25
**K(+58.01)TQFGC(+57.02)PAYEGYC(+57.02)NDHC(+57.02)NDIER**	58,44	2791,106	22	4,8	931,3804	14,21	0	1	4488	150211-Fr_I_Helo-red-alk-tryp-001.raw	4	4	P0C2F3.1:P69940.2	Carboxymethyl (KW X@N-term); Carbamidomethylation	K1:Carboxymethyl (KW X@N-term):1000.00;C6:Carbamidomethylation:1000.00;C13:Carbamidomethylation:1000.00;C1

															7:Carbamidomethylation:1000.00
**VKPPTAKPPPQYFEFK**	58,34	1873,014	16	-1,1	469,2602	18,18	5,49E+08	1	5966	150211-Fr_I_Helo-red-alk-tryp-001.raw	5	5	JAW07031.1:JAW06995.1		
**AEIDFSGIPE(+37.95)DIIK**	58,18	1583,74	14	-0,2	792,8769	38,09	0	1	12973	150211-Fr_I_Helo-red-alk-tryp-001.raw	28	28	JAW06987.1:JAW07038.1:JAW06992.1:P84190.1	Replacement of 2 protons by calcium	E10:Replacement of 2 protons by calcium:24.44
**EALAPEPAPEPAPEP**	58,18	1513,73	15	-0,4	757,8719	34,05	0	1	11495	150211-Fr_I_Helo-red-alk-tryp-001.raw	51	51	JAW07022.1:P86821.1		
**APEPAPEAAPEAAPE(+21.98)PAAAAPE**	58,04	2074,946	22	-3,5	1038,477	15,93	0	1	5119	150211-Fr_I_Helo-red-alk-tryp-001.raw	1	1	JAW07022.1:P86821.1	Sodium adduct	E15:Sodium adduct:25.39
**S(+59.02)SGYC(+57.02)AWPAC(+57.02)YC(+57.02)YGLPDSVKIWTSETNK**	57,97	3358,456	28	2,6	1120,495	49,46	0	1	17008	150211-Fr_I_Helo-red-alk-tryp-001.raw	1	1	AAC25688.1:O77463.1:AAC25689.1	Aminoethylcysteine; Carbamidomethylation	S1:Aminoethylcysteine:0.00;C5:Carbamidomethylation:1000.00;C10:Carbamidomethylation:1000.00;C12:Carbamidomethylation:1000.00
**LVLIGLTVLKDDLPFIR**	57,73	1924,176	17	2	963,0972	54,72	0	1	18743	150211-Fr_I_Helo-red-alk-tryp-001.raw	2	2	AMO02560.1		
**E(-18.01)IDFSGIPEDIIK**	57,66	1456,745	13	-0,3	729,3795	46,88	8,48E+08	1	16121	150211-Fr_I_Helo-red-alk-tryp-001.raw	15	15	JAW06987.1:JAW07038.1:JAW06992.1:P84190.1	Pyro-glu from E	E1:Pyro-glu from E:1000.00
**GAPAQYLDISYIR**	57,57	1465,757	13	-0,6	733,8851	33,44	1,83E+08	1	11272	150211-Fr_I_Helo-red-alk-tryp-001.raw	1	1	JAW06991.1:JAW07032.1:JAG85159.1		
**FSAPHLIFPEGMK**	57,54	1472,749	13	0,4	491,9236	36,59	0	1	12411	150211-Fr_I_Helo-red-alk-tryp-001.raw	1	1	JAW07031.1:JAW06995.1		
**LTAAGYC(+57.02)NTEC(+57.02)TLK**	57,52	1600,723	14	-0,8	801,3679	30,71	0	1	10311	150211-Fr_I_Helo-red-alk-tryp-001.raw	37	37	AAC25688.1:O77463.1:AAC25689.1	Carbamidomethylation	C7:Carbamidomethylation:1000.00;C11:Carbamidomethylation:1000.00
**S(+28.03)GAYFWPASDIPIKDTF**	57,18	1941,951	17	-0,5	971,9824	51,92	0	1	17830	150211-Fr_I_Helo-red-alk-tryp-001.raw	1	1	XP_023242894.1	Ethylation	S1:Ethylation:140.79
**C(+57.02)ISANLVGILFYDILR**	57,08	1866,007	16	0	623,0097	59,53	0	1	20310	150211-Fr_I_Helo-red-alk-tryp-001.raw	1	1	JAW07019.1:JAW06978.1	Carbamidomethylation	C1:Carbamidomethylation:1000.00
**VQNIFDTLELGIK**	57,06	1488,819	13	-0,7	745,4161	42,22	0	1	14464	150211-Fr_I_Helo-red-alk-tryp-001.raw	11	11	AIJ02113.2		
**DTDLIFLITAR**	56,92	1276,703	11	-1,1	639,3579	43,34	0	1	14868	150211-Fr_I_Helo-red-alk-tryp-001.raw	10	10	AIJ02109.2:P86392.3:V9Z9A3.1:AHE40589.1:AHE40588.1:JAG85220.1:AHE40596.1:V9ZAY0.1		
**FSC(+57.02)FIRPAGFC(+57.02)D**	56,84	1475,633	12	0,1	738,8237	33,03	0	1	11126	150211-Fr_I_Helo-red-alk-tryp-001.raw	1	1	JAW07016.1:JAW06979.1:P68411.1:1913435A:P68410.1:AAB36322.1	Carbamidomethylation	C3:Carbamidomethylation:1000.00;C11:Carbamidomethylation:1000.00
**LTSESEYAC(+57.02)PAIDKFC(+57.02)EDHC(+57.02)AA**	56,72	2573,051	22	4,8	858,695	18,79	0	1	6199	150211-Fr_I_Helo-red-alk-tryp-001.raw	2	2	Q0GY42.1:ABE98265.1:JAG85223.1	Carbamidomethylation	C9:Carbamidomethylation:1000.00;C16:Carbamidomethylation:1000.00;C20:Carbamidomethylation:1000.00
**S(+58.03)SGYC(+57.02)AWPAC(+57.02)YC(+57.02)YGLPDSVK**	56,68	2397,994	20	-3,2	1200,001	45,7	0	1	15708	150211-Fr_I_Helo-red-alk-tryp-001.raw	35	35	AAC25688.1:O77463.1:AAC25689.1	2 3-dihydro-2 2-dimethyl-7-benzofuranol N-methyl carbamate; Carbamidomethylation	S1:2 3-dihydro-2 2-dimethyl-7-benzofuranol N-methyl carbamate:0.00;C5:Carbamidomethylation:1000.00;C10:Carb

															amidomethylation:1000.00;C12:Carbamidomethylation:1000.00
**FTGVQNLLDTLNLGIK**	56,67	1744,972	16	-0,3	582,6646	49,73	0	1	17101	150211-Fr_I_Helo-red-alk-tryp-001.raw	1	1	AIJ02110.2		
**KT(+59.02)QFGC(+57.02)PAYEGYC(+57.02)NDHC(+57.02)NDIER**	56,53	2792,12	22	-3,8	931,7104	14,88	0	1	4731	150211-Fr_I_Helo-red-alk-tryp-001.raw	3	3	P0C2F3.1:P69940.2	Aminoethylcysteine; Carbamidomethylation	T2:Aminoethylcysteine:1000.00;C6:Carbamidomethylation:1000.00;C13:Carbamidomethylation:1000.00;C17:Carbamidomethylation:1000.00
**QNEPSFIEDS(+58.03)AIPGHQQVLDPVDLVK**	56,4	2932,469	26	-2,3	978,4948	45,99	0	1	15810	150211-Fr_I_Helo-red-alk-tryp-001.raw	4	4	AIJ02110.2	2 3-dihydro-2 2-dimethyl-7-benzofuranol N-methyl carbamate	S10:2 3-dihydro-2 2-dimethyl-7-benzofuranol N-methyl carbamate:1.86
**AEIDFSGIPEDIIKQ**	55,67	1673,851	15	-0,1	837,9328	39,05	3,37E+09	1	13315	150211-Fr_I_Helo-red-alk-tryp-001.raw	92	92	JAW06987.1:JAW07038.1:JAW06992.1		
**AEIDFSGIPEDIIKEI**	55,59	1787,919	16	-0,5	894,9664	51,12	1,28E+09	1	17564	150211-Fr_I_Helo-red-alk-tryp-001.raw	20	20	P84190.1		
**LSS(+59.02)AANMMQMEWDDELAAVAQAHANQC(+57.02)K**	55,13	3178,376	28	1,2	795,6023	37,1	0	1	12604	150211-Fr_I_Helo-red-alk-tryp-001.raw	1	1	JAW07031.1:JAW06995.1	Aminoethylcysteine; Carbamidomethylation	S3:Aminoethylcysteine:0.00;C27:Carbamidomethylation:1000.00
**YFFTLPQASC(+57.02)LREDC(+57.02)PNSG**	55,13	2260,988	19	1,1	1131,503	42,77	0	1	14661	150211-Fr_I_Helo-red-alk-tryp-001.raw	1	1	JAW07039.1	Carbamidomethylation	C10:Carbamidomethylation:1000.00;C15:Carbamidomethylation:1000.00
**VDFTGIADSIIK**	55,01	1277,687	12	-0,1	639,8505	37,38	0	1	12706	150211-Fr_I_Helo-red-alk-tryp-001.raw	9	9	JAW07002.1:P86824.1		
**FGPNVAGETAAS(+58.03)EVNFHFSAPHLIFPEGMK**	54,97	3258,568	30	-1,5	652,7199	51,93	0	1	17834	150211-Fr_I_Helo-red-alk-tryp-001.raw	2	2	JAW07031.1:JAW06995.1	2 3-dihydro-2 2-dimethyl-7-benzofuranol N-methyl carbamate	S12:2 3-dihydro-2 2-dimethyl-7-benzofuranol N-methyl carbamate:38.64
**KC(+57.02)VEHLLSLPR**	54,77	1350,744	11	0,1	451,2554	30,7	0	1	10307	150211-Fr_I_Helo-red-alk-tryp-001.raw	1	1	AIJ02110.2:JAG85213.1	Carbamidomethylation	C2:Carbamidomethylation:1000.00
**KEALAPEPAPEPAPEP**	54,53	1641,825	16	-0,7	821,9192	23,26	2,16E+09	1	7854	150211-Fr_I_Helo-red-alk-tryp-001.raw	90	90	JAW07022.1:P86821.1		
**AEID(+37.95)FSGIPEDIIK**	54,46	1583,74	14	1,7	528,9213	38,76	9,13E+05	1	13212	150211-Fr_I_Helo-red-alk-tryp-001.raw	12	12	JAW06987.1:JAW07038.1:JAW06992.1:P84190.1	Replacement of 2 protons by calcium	D4:Replacement of 2 protons by calcium:24.55
**FFLNKDEASC(+57.02)VVSAC(+57.02)**	54,34	1745,775	15	-2,5	873,8928	17,34	0	1	5649	150211-Fr_I_Helo-red-alk-tryp-001.raw	1	1	AIJ02112.2	Carbamidomethylation	C10:Carbamidomethylation:1000.00;C15:Carbamidomethylation:1000.00
**EALAPEPAPE(+21.98)PAPEP**	54,09	1535,712	15	-1,5	768,8621	16,76	0	1	5428	150211-Fr_I_Helo-red-alk-tryp-001.raw	2	2	JAW07022.1:P86821.1	Sodium adduct	E10:Sodium adduct:21.91
**IDFSGIPEDIIK**	53,98	1345,713	12	0,8	673,8643	38	2,12E+09	1	12936	150211-Fr_I_Helo-red-alk-tryp-001.raw	93	93	JAW06987.1:JAW07038.1:JAW06992.1:P84190.1		
**AEIDFSGIPEDIIK(+27.99)QIK**	53,88	1943,025	17	3,4	972,5231	49,22	0	1	16925	150211-Fr_I_Helo-red-alk-tryp-001.raw	1	1	JAW06987.1:JAW07038.1:JAW06992.1	Formylation	K14:Formylation:18.00
**T(+58.01)QFGC(+57.02)PAYEGYC(+57.02)NDHC(+57.02)NDIER**	53,76	2663,011	21	4,9	1332,519	15,32	0	1	4895	150211-Fr_I_Helo-red-alk-tryp-001.raw	2	2	P0C2F3.1:P69940.2	Carboxymethyl (KW X@N-term); Carbamidomethylation	T1:Carboxymethyl (KW X@N-term):1000.00;C5:Carbamidomethylation:1000.00;C12:Carbamidomethylation:1000.00;C1

															6:Carbamidomethylation:1000.00
**DGEC(+57.02)HGFK**	53,73	948,3759	8	-0,3	475,1951	15,39	0	1	4921	150211-Fr_I_Helo-red-alk-tryp-001.raw	126	126	P0C2F3.1:P69940.2:Q5G8A6.1:JAG85216.1:AAW72464.1:AAW72465.1	Carbamidomethylation	C4:Carbamidomethylation:1000.00
**ADVDFTGIAESIIK(+43.01)**	53,65	1520,772	14	1	761,3942	37,94	0	1	12913	150211-Fr_I_Helo-red-alk-tryp-001.raw	1	1		Carbamylation	K14:Carbamylation:90.97
**ALAPEPAPEPAPEPAPEA**	53,49	1752,857	18	-0,6	877,4352	34,89	0	1	11798	150211-Fr_I_Helo-red-alk-tryp-001.raw	7	7	JAW07022.1:P86821.1		
**T(+114.04)ILVGVLGVANLGR**	53,38	1494,888	14	0	748,4514	37,96	0	1	12921	150211-Fr_I_Helo-red-alk-tryp-001.raw	2	2	AMO02558.1:JAW07025.1:JAW06984.1	Ubiquitin	T1:Ubiquitin:1000.00
**AEIDFSGIP(+15.99)EDIIK**	53,36	1561,788	14	-1,8	781,8996	38,47	0	1	13109	150211-Fr_I_Helo-red-alk-tryp-001.raw	19	19	JAW06987.1:JAW07038.1:JAW06992.1:P84190.1	Oxidation or Hydroxylation	P9:Oxidation or Hydroxylation:41.39
**FGPNVAGETAAS(+59.02)EVNFHFSAPHLIFPEGMK**	53,05	3259,558	30	1,8	652,92	50,99	0	1	17522	150211-Fr_I_Helo-red-alk-tryp-001.raw	4	4	JAW07031.1:JAW06995.1	Aminoethylcysteine	S12:Aminoethylcysteine:0.00
**KGSSGYC(+57.02)AWPAC(+57.02)Y**	53,05	1505,607	13	-2	753,8091	33,07	0	1	11140	150211-Fr_I_Helo-red-alk-tryp-001.raw	1	1	AAC25688.1:O77463.1:AAC25689.1:AAW72453.1:Q5G8B8.1:AAB29128.1:P56612.1:AIA81145.1:CAA46982.1:P15226.2:P0DMM8.1:1NPI:1B7D:AAB36321.1:AAB36318.1:P56611.1:JAG85191.1:JAG85210.1	Carbamidomethylation	C7:Carbamidomethylation:1000.00;C12:Carbamidomethylation:1000.00
**AEID(+37.96)FSGIPEDIIK**	52,97	1583,748	14	-1,8	528,9225	40,89	0	1	13981	150211-Fr_I_Helo-red-alk-tryp-001.raw	4	4	JAW06987.1:JAW07038.1:JAW06992.1:P84190.1	Replacement of proton by potassium	D4:Replacement of proton by potassium:14.08
**AEIDFSGIPEDIIKQI**	52,93	1786,935	16	0,1	894,4749	49,99	1,74E+08	1	17187	150211-Fr_I_Helo-red-alk-tryp-001.raw	2	2	JAW06987.1:JAW07038.1:JAW06992.1		
**S(+162.08)GYC(+57.02)AWPAC(+57.02)YC(+57.02)YGLPDSVK**	52,8	2415,014	19	0,2	806,0121	42,39	0	1	14525	150211-Fr_I_Helo-red-alk-tryp-001.raw	15	15	AAC25688.1:O77463.1:AAC25689.1	O-Pinacolylmethylphosphonylation; Carbamidomethylation	S1:O-Pinacolylmethylphosphonylation:0.00;C4:Carbamidomethylation:1000.00;C9:Carbamidomethylation:1000.00;C11:Carbamidomethylation:1000.00
**ESLIPGEEVFDFGHVVGNL(+21.98)**	52,6	2078,992	19	-0,3	694,0045	52,18	0	1	17917	150211-Fr_I_Helo-red-alk-tryp-001.raw	1	1	AIJ02112.2	Sodium adduct	L19:Sodium adduct:7.49
**S(+28.03)GAYFWPASDIPIKD**	52,44	1693,835	15	-1,5	847,9235	46	0	1	15815	150211-Fr_I_Helo-red-alk-tryp-001.raw	2	2	XP_023242894.1	Ethylation	S1:Ethylation:91.09
**LS(+58.03)SAANMMQMEWDDELAAVAQAHANQC(+57.02)K**	52,36	3177,386	28	-2,9	795,3515	43,02	0	1	14752	150211-Fr_I_Helo-red-alk-tryp-001.raw	1	1	JAW07031.1:JAW06995.1	2 3-dihydro-2 2-dimethyl-7-benzofuranol N-methyl carbamate; Carbamidomethylation	S2:2 3-dihydro-2 2-dimethyl-7-benzofuranol N-methyl carbamate:0.00;C27:Carbamidomethylation:1000.00
**EIDFSGIPEDIIK**	52,35	1474,756	13	-0,4	738,3847	42,25	0	1	14475	150211-Fr_I_Helo-red-alk-tryp-001.raw	25	25	JAW06987.1:JAW07038.1:JAW06992.1:P84190.1		

**APEPAPEAAPE(+37.96)AAPEPAAAPE**	52,35	2090,92	22	-0,8	1046,466	16,14	0	1	5196	150211-Fr_I_Helo-red-alk-tryp-001.raw	1	1	JAW07022.1:P86821.1	Replacement of proton by potassium	E11:Replacement of proton by potassium:0.00
**QN(+.98)EPSFIEDSAIPGHQQVLDPVDLVK**	52,29	2875,424	26	4,8	959,4865	48,09	0	1	16538	150211-Fr_I_Helo-red-alk-tryp-001.raw	2	2	AIJ02110.2	Deamidation (NQ)	N2:Deamidation (NQ):0.00
**GT(+114.04)ILVGVLGVANLGR**	52,14	1551,91	15	1,7	776,9634	47,39	0	1	16301	150211-Fr_I_Helo-red-alk-tryp-001.raw	1	1	AMO02558.1:JAW07025.1:JAW06984.1	Ubiquitin	T2:Ubiquitin:1000.00
**VTGSAIGIALGASAC(+57.02)NK**	52,12	1588,824	17	0,5	795,4198	14,82	0	1	4712	150211-Fr_I_Helo-red-alk-tryp-001.raw	5	5	JAG85190.1	Carbamidomethylation	C15:Carbamidomethylation:1000.00
**GS(+59.02)SGYC(+57.02)AWPAC(+57.02)YC(+57.02)YGLPDSVK**	51,93	2456,006	21	2,7	819,6781	43,95	0	1	15088	150211-Fr_I_Helo-red-alk-tryp-001.raw	3	3	AAC25688.1:O77463.1:AAC25689.1	Aminoethylcysteine; Carbamidomethylation	S2:Aminoethylcysteine:0.00;C6:Carbamidomethylation:1000.00;C11:Carbamidomethylation:1000.00;C13:Carbamidomethylation:1000.00
**FS(+58.03)C(+57.02)FIRPAGFC(+57.02)DGYC(+57.02)K**	51,5	2041,872	16	-0,9	681,6307	37,5	0	1	12749	150211-Fr_I_Helo-red-alk-tryp-001.raw	6	6	JAW07016.1:JAW06979.1:P68411.1:1913435A:P68410.1:AAB36322.1	2 3-dihydro-2 2-dimethyl-7-benzofuranol N-methyl carbamate; Carbamidomethylation	S2:2 3-dihydro-2 2-dimethyl-7-benzofuranol N-methyl carbamate:1000.00;C3:Carbamidomethylation:1000.00;C11:Carbamidomethylation:1000.00;C15:Carbamidomethylation:1000.00
**LTSQ(+.98)SEYAC(+57.02)PAIEK(+43.01)FC(+57.02)EDHC(+57.02)AAK**	51,45	2758,167	23	5,1	920,401	32,48	0	1	10927	150211-Fr_I_Helo-red-alk-tryp-001.raw	1	1	ABV64385.1:ABE98262.1:Q0GY45.1	Deamidation (NQ); Carbamidomethylation; Carbamylation	Q4:Deamidation (NQ):1000.00;C9:Carbamidomethylation:1000.00;K14:Carbamylation:46.57;C16:Carbamidomethylation:1000.00;C20:Carbamidomethylation:1000.00
**ADVDFTGIADSIIK(+114.04)**	51,38	1577,794	14	0,1	789,9042	38,06	0	1	12959	150211-Fr_I_Helo-red-alk-tryp-001.raw	1	1	JAW07002.1:P86824.1	Ubiquitin	K14:Ubiquitin:22.45
**C(+57.02)SATYPGLC(+57.02)K**	51,35	1155,505	10	-0,3	578,7597	25,51	0	1	8624	150211-Fr_I_Helo-red-alk-tryp-001.raw	98	98	JAW07031.1:JAW06995.1	Carbamidomethylation	C1:Carbamidomethylation:1000.00;C9:Carbamidomethylation:1000.00
**KLGELQDDGTVAYNT(+59.02)AGISLGSGVC(+57.02)K**	51,32	2711,326	26	2,7	904,785	35,83	0	1	12136	150211-Fr_I_Helo-red-alk-tryp-001.raw	1	1	AIJ02110.2:JAG85213.1	Aminoethylcysteine; Carbamidomethylation	T15:Aminoethylcysteine:17.91;C25:Carbamidomethylation:1000.00
**AEID(+15.99)FSGIPEDIIK**	51,2	1561,788	14	1,1	781,9019	36,58	1,22E+09	1	12408	150211-Fr_I_Helo-red-alk-tryp-001.raw	20	20	JAW06987.1:JAW07038.1:JAW06992.1:P84190.1	Oxidation or Hydroxylation	D4:Oxidation or Hydroxylation:29.24
**KEGYAMDHEGC(+57.02)K**	51,13	1423,586	12	-0,1	712,8002	5,69	3,25E+06	1	1417	150211-Fr_I_Helo-red-alk-tryp-001.raw	7	7	JAW07016.1:JAW06979.1:P68411.1:1913435A:P68410.1:AAB36322.1:AAW72453.1:Q5G8B8.1:C0HJM9.1:JAG85185.1:CEF39517.1:P60276.1	Carbamidomethylation	C11:Carbamidomethylation:1000.00
**GS(+58.03)SGYC(+57.02)AWPAC(+57.02)YC(+57.02)YGLPNWVK**	50,99	2553,079	21	-3,7	852,0304	48,9	0	1	16814	150211-Fr_I_Helo-red-alk-tryp-001.raw	1	1	AAW72453.1:Q5G8B8.1:AAB29128.1:P56612.1:AIA81145.1:CAA46982.1:P15226.2:P0DMM8.1:1NPI:1	2 3-dihydro-2 2-dimethyl-7-benzofuranol N-methyl carbamate; Carbamidomethylation	S2:2 3-dihydro-2 2-dimethyl-7-benzofuranol N-methyl carbamate:0.00;C6:Carbamidomethylation:1000.00;C11:Carbamidomethylation:1000.00;C1

													B7D:AAB36321.1:AAB36318.1:P56611.1:JAG85191.1:JAG85210.1		3:Carbamidomethylation:1000.00
**F(+43.01)SC(+57.02)FIR(+14.02)PAGFC(+57.02)DGYC(+57.02)K**	50,81	2040,864	16	-0,2	1021,439	36,11	4,11E+09	1	12234	150211-Fr_I_Helo-red-alk-tryp-001.raw	5	5	JAW07016.1:JAW06979.1:P68411.1:1913435A:P68410.1:AAB36322.1	Carbamylation; Carbamidomethylation; Methylation(KR)	F1:Carbamylation:170.25;C3:Carbamidomethylation:1000.00;R6:Methylation(KR):228.69;C11:Carbamidomethylation:1000.00;C15:Carbamidomethylation:1000.00
**ADIIMLLTGR**	50,76	1101,622	10	0,2	551,8182	36,06	0	1	12218	150211-Fr_I_Helo-red-alk-tryp-001.raw	7	7	AMO02558.1:JAW07025.1:JAW06984.1		
**ADIIMLITGR**	50,76	1101,622	10	0,2	551,8182	36,06	0	1	12218	150211-Fr_I_Helo-red-alk-tryp-001.raw	7	7			
**S(+58.03)GINYLVLQVHYADVTR**	50,73	2005,051	17	-2,3	669,356	37	0	1	12567	150211-Fr_I_Helo-red-alk-tryp-001.raw	3	3	XP_023239442.1	2 3-dihydro-2 2-dimethyl-7-benzofuranol N-methyl carbamate	S1:2 3-dihydro-2 2-dimethyl-7-benzofuranol N-methyl carbamate:1000.00
**AS(+58.03)SGYC(+57.02)AWPAC(+57.02)YC(+57.02)YGVPDHIK**	50,58	2519,058	21	-2,3	1260,533	37	0	1	12565	150211-Fr_I_Helo-red-alk-tryp-001.raw	6	6	JAW07016.1:JAW06979.1:P68411.1:1913435A:P68410.1:AAB36322.1	2 3-dihydro-2 2-dimethyl-7-benzofuranol N-methyl carbamate; Carbamidomethylation	S2:2 3-dihydro-2 2-dimethyl-7-benzofuranol N-methyl carbamate:0.00;C6:Carbamidomethylation:1000.00;C11:Carbamidomethylation:1000.00;C13:Carbamidomethylation:1000.00
**AEIDFSGIPE(+21.98)DII**	50,55	1439,68	13	-1	720,8463	47,03	0	1	16175	150211-Fr_I_Helo-red-alk-tryp-001.raw	1	1	JAW06987.1:JAW07038.1:JAW06992.1:P84190.1	Sodium adduct	E10:Sodium adduct:14.02
**TQFGC(+57.02)PAYEGYC(+57.02)NNHC(+57.02)QD(+43.99)IER**	50,51	2662,027	21	-0,4	888,3492	17,15	0	1	5575	150211-Fr_I_Helo-red-alk-tryp-001.raw	1	1	Q5G8A6.1:JAG85216.1:AAW72464.1:AAW72465.1	Carbamidomethylation; Carboxylation (DKW)	C5:Carbamidomethylation:1000.00;C12:Carbamidomethylation:1000.00;C16:Carbamidomethylation:1000.00;D18:Carboxylation (DKW):1000.00
**LS(+59.02)SAANMMQMEWDDELAAVAQAHANQC(+57.02)K**	50,47	3178,376	28	0	795,6013	43,24	0	1	14832	150211-Fr_I_Helo-red-alk-tryp-001.raw	2	2	JAW07031.1:JAW06995.1	Aminoethylcysteine; Carbamidomethylation	S2:Aminoethylcysteine:0.00;C27:Carbamidomethylation:1000.00
**I(+58.01)TC(+57.02)FLTAAGYC(+57.02)NTEC(+57.02)TLKK**	50,42	2308,054	19	4,5	770,3619	34,76	0	1	11751	150211-Fr_I_Helo-red-alk-tryp-001.raw	2	2	AAC25688.1:O77463.1:AAC25689.1	Carboxymethyl (KW X@N-term); Carbamidomethylation	I1:Carboxymethyl (KW X@N-term):137.15;C3:Carbamidomethylation:1000.00;C11:Carbamidomethylation:1000.00;C15:Carbamidomethylation:1000.00
**GS(+58.03)GINYLVLQVHYADVTR**	50,37	2062,072	18	-1,3	688,3637	37,03	0	1	12578	150211-Fr_I_Helo-red-alk-tryp-001.raw	2	2	XP_023239442.1	2 3-dihydro-2 2-dimethyl-7-benzofuranol N-methyl carbamate	S2:2 3-dihydro-2 2-dimethyl-7-benzofuranol N-methyl carbamate:1000.00
**SSLLFLTPIFVK**	50,16	1363,812	12	-0,1	682,913	50,22	0	1	17266	150211-Fr_I_Helo-red-alk-tryp-001.raw	3	3	JAW07036.1		
**KYFFTLPQASC(+57.02)LR**	50,12	1629,834	13	0,2	544,2853	36,08	0	1	12226	150211-Fr_I_Helo-red-alk-tryp-001.raw	1	1	JAW07039.1	Carbamidomethylation	C11:Carbamidomethylation:1000.00

**APEAAPEAAPEPAAAAPE**	50,01	1658,779	18	-0,5	830,3962	19,26	5,28E+08	1	6381	150211-Fr_I_Helo-red-alk-tryp-001.raw	68	68	JAW07022.1:P86821.1		
**ETRPITSWHTIGILM(+15.99)RWDK(+42.02)ELQISYVFK**	49,97	3504,834	28	2,5	701,9758	48,27	0	1	16600	150211-Fr_I_Helo-red-alk-tryp-001.raw	1	1	JAW07031.1:JAW06995.1	Oxidation (M); Guanidination	M15:Oxidation (M):1000.00;K19:Guanidination:77.76
**AEIDFS(+79.96)GIPEDIIK**	49,77	1625,749	14	-1,7	813,8806	45,24	0	1	15545	150211-Fr_I_Helo-red-alk-tryp-001.raw	1	1	JAW06987.1:JAW07038.1:JAW06992.1:P84190.1	Sulfation	S6:Sulfation:1000.00
**EALAPEPAPEP**	49,56	1119,545	11	-0,6	560,7793	15,53	1,20E+09	1	4970	150211-Fr_I_Helo-red-alk-tryp-001.raw	126	126	JAW07022.1:P86821.1		
**WDKELQISYVFK**	49,52	1554,808	12	-0,6	778,4109	36,04	0	1	12209	150211-Fr_I_Helo-red-alk-tryp-001.raw	1	1	JAW07031.1:JAW06995.1		
**ITAVEIPDPFWVDAIR**	49,42	1840,972	16	0,8	921,4941	52,38	0	1	17983	150211-Fr_I_Helo-red-alk-tryp-001.raw	1	1	JAW06991.1:JAW07032.1:JAG85159.1		
**KLGELQDDGTVAYN(+.98)TAGISLGSGVC(+57.02)K(+21.98)**	49,38	2675,273	26	4,9	892,7691	35,91	0	1	12163	150211-Fr_I_Helo-red-alk-tryp-001.raw	1	1	AIJ02110.2:JAG85213.1	Deamidation (NQ); Carbamidomethylation; Sodium adduct	N14:Deamidation (NQ):0.00;C25:Carbamidomethylation:1000.00;K26:Sodium adduct:0.00
**E(+41.03)GYPADSKGC(+57.02)KITC(+57.02)FLTAAGYC(+57.02)NTEC(+57.02)TLKK**	49,24	3483,593	30	-4,4	871,9017	36,89	0	1	12526	150211-Fr_I_Helo-red-alk-tryp-001.raw	1	1	AAC25688.1:O77463.1:AAC25689.1	Amidination of lysines or N-terminal amines with methyl acetimidate; Carbamidomethylation	E1:Amidination of lysines or N-terminal amines with methyl acetimidate:8.82;C10:Carbamidomethylation:1000.00;C14:Carbamidomethylation:1000.00;C22:Carbamidomethylation:1000.00;C26:Carbamidomethylation:1000.00
**KGSSGYC(+57.02)AWPAC(+57.02)YC(+57.02)YGLPD**	49,17	2210,886	19	0,2	1106,45	38,98	0	1	13290	150211-Fr_I_Helo-red-alk-tryp-001.raw	1	1	AAC25688.1:O77463.1:AAC25689.1	Carbamidomethylation	C7:Carbamidomethylation:1000.00;C12:Carbamidomethylation:1000.00;C14:Carbamidomethylation:1000.00
**EALAPEPAPEPAPE(+37.95)PAPEA**	49,07	1919,846	19	-1,7	640,955	35,08	0	1	11863	150211-Fr_I_Helo-red-alk-tryp-001.raw	1	1	JAW07022.1:P86821.1	Replacement of 2 protons by calcium	E14:Replacement of 2 protons by calcium:16.51
**AEIDFSGIPEDIIKQIKETNAKPPA**	48,98	2723,438	25	0,3	681,867	48,7	4,93E+08	1	16745	150211-Fr_I_Helo-red-alk-tryp-001.raw	1	1	JAW06987.1:JAW07038.1:JAW06992.1		
**FSTC(+57.02)SVENIK**	48,7	1183,554	10	-0,2	592,7843	30,02	0	1	10078	150211-Fr_I_Helo-red-alk-tryp-001.raw	20	20	AMO02558.1:JAW07025.1:JAW06984.1	Carbamidomethylation	C4:Carbamidomethylation:1000.00
**AIIT(+59.02)VVDNGIMIGC(+57.02)GIFDVASPSVK**	48,7	2634,358	25	1,1	879,1277	49,29	0	1	16949	150211-Fr_I_Helo-red-alk-tryp-001.raw	1	1	JAW07019.1:JAW06978.1	Aminoethylcysteine; Carbamidomethylation	T4:Aminoethylcysteine:88.40;C14:Carbamidomethylation:1000.00
**YC(+57.02)YGLPNWVK**	48,69	1298,612	10	-1	650,3126	33,91	0	1	11445	150211-Fr_I_Helo-red-alk-tryp-001.raw	4	4	AAW72453.1:Q5G8B8.1:AAB29128.1:P56612.1:AIA81145.1:CAA46982.1:P15226.2:P0DMM8.1:1NPI:1B7D:AAB36321.1:AAB36318.1:P56611.1:JAG85191.1:JAG85210.1	Carbamidomethylation	C2:Carbamidomethylation:1000.00

**E(sub Q)NEPSFIEDSAIPGHQQVLDPVDLVK**	48,67	2875,424	26	5,5	959,4872	48,86	0	1	16798	150211-Fr_I_Helo-red-alk-tryp-001.raw	6	6	AIJ02110.2		
**DVTMTGSRPFTTQK**	48,66	1567,767	14	0,1	523,5961	30,34	0	1	10185	150211-Fr_I_Helo-red-alk-tryp-001.raw	6	6	JAW07031.1:JAW06995.1		
**TQ(+.98)FGC(+57.02)PAYEGYC(+57.02)NDHC(+57.02)N(+.98)DIER(-.98)**	48,57	2605,99	21	4,9	869,6748	26,57	0	1	8963	150211-Fr_I_Helo-red-alk-tryp-001.raw	1	1	P0C2F3.1:P69940.2	Deamidation (NQ); Carbamidomethylation; Amidation	Q2:Deamidation (NQ):98.13;C5:Carbamidomethylation:1000.00;C12:Carbamidomethylation:1000.00;C16:Carbamidomethylation:1000.00;N17:Deamidation (NQ):61.69;R21:Amidation:1000.00
**LSC(+57.02)FIRPSGYC(+57.02)GR**	48,46	1571,734	13	-0,3	524,9183	31,38	0	1	10540	150211-Fr_I_Helo-red-alk-tryp-001.raw	1	1	AAW72453.1:Q5G8B8.1:AAB29128.1:P56612.1:AIA81145.1:CAA46982.1:P15226.2:P0DMM8.1:1NPI:1B7D:AAB36321.1	Carbamidomethylation	C3:Carbamidomethylation:1000.00;C11:Carbamidomethylation:1000.00
**GGLC(+57.02)AFLIGR**	48,46	1062,565	10	0,2	532,2896	31,56	0	1	10602	150211-Fr_I_Helo-red-alk-tryp-001.raw	45	45	JAW07031.1:JAW06995.1	Carbamidomethylation	C4:Carbamidomethylation:1000.00
**IFDIADEILPITTNFFR**	48,32	2024,062	17	-0,6	1013,038	58,23	0	1	19899	150211-Fr_I_Helo-red-alk-tryp-001.raw	3	3	JAW07023.1		
**EAAPEAAPEPAAAAPE**	48,26	1490,689	16	-0,4	746,3514	31,33	0	1	10523	150211-Fr_I_Helo-red-alk-tryp-001.raw	6	6	JAW07022.1:P86821.1		
**V(+43.01)TESDK(+14.02)QTILETHNQLR**	48,25	2068,055	17	0,2	518,0211	30,18	0	1	10131	150211-Fr_I_Helo-red-alk-tryp-001.raw	3	3	JAW07031.1:JAW06995.1	Carbamylation; Methylation(KR)	V1:Carbamylation:1000.00;K6:Methylation(KR):88.86
**FSC(+57.02)FIRPAGF**	48,24	1200,575	10	0,1	601,2948	33,41	0	1	11260	150211-Fr_I_Helo-red-alk-tryp-001.raw	13	13	JAW07016.1:JAW06979.1:P68411.1:1913435A:P68410.1:AAB36322.1	Carbamidomethylation	C3:Carbamidomethylation:1000.00
**ITC(+57.02)FLTAAGYC(+57.02)NTE(+17.03)C(+57.02)TLK**	48,19	2138,98	18	-1,6	713,9995	35,79	0	1	12121	150211-Fr_I_Helo-red-alk-tryp-001.raw	1	1	AAC25688.1:O77463.1:AAC25689.1	Carbamidomethylation; Replacement of proton with ammonium ion	C3:Carbamidomethylation:1000.00;C11:Carbamidomethylation:1000.00;E14:Replacement of proton with ammonium ion:31.27;C15:Carbamidomethylation:1000.00
**FGPNVAGETAASEVNFHFSAPHLIFPE**	48,1	2884,382	27	-1,3	962,4667	52,41	2,82E+08	1	17993	150211-Fr_I_Helo-red-alk-tryp-001.raw	3	3	JAW07031.1:JAW06995.1		
**KKQNEPS(+58.03)FIEDSAIPGHQQVLDPVDLVK**	48,1	3188,659	28	-1,2	798,1711	37,85	0	1	12881	150211-Fr_I_Helo-red-alk-tryp-001.raw	1	1	AIJ02110.2	2 3-dihydro-2 2-dimethyl-7-benzofuranol N-methyl carbamate	S7:2 3-dihydro-2 2-dimethyl-7-benzofuranol N-methyl carbamate:39.44
**IDFSGIPE(+21.98)DIIK**	47,94	1367,695	12	1,1	684,8554	37,77	0	1	12847	150211-Fr_I_Helo-red-alk-tryp-001.raw	1	1	JAW06987.1:JAW07038.1:JAW06992.1:P84190.1	Sodium adduct	E8:Sodium adduct:12.28
**S(+58.03)SGYC(+57.02)AWPAC(+57.02)YC(+57.02)YGLPNWVK**	47,94	2496,058	20	-1,7	1249,034	48,84	0	1	16792	150211-Fr_I_Helo-red-alk-tryp-001.raw	1	1	AAW72453.1:Q5G8B8.1:AAB29128.1:P56612.1:AIA81145.1:CAA46982.1:P15226.	2 3-dihydro-2 2-dimethyl-7-benzofuranol N-methyl carbamate; Carbamidomethylation	S1:2 3-dihydro-2 2-dimethyl-7-benzofuranol N-methyl carbamate:0.00;C5:Carbamidomethylation:1000.00;C10:Carb

													2:P0DMM8.1:1NPI:1B7D:AAB36321.1:AAB36318.1:P56611.1:JAG85191.1:JAG85210.1		amidomethylation:1000.00;C12:Carbamidomethylation:1000.00
**FEHDSGDQR**	47,9	1089,448	9	0,4	545,7313	5,56	0	1	1377	150211-Fr_I_Helo-red-alk-tryp-001.raw	23	23	JAW07031.1:JAW06995.1:JAG85161.1		
**EALAPE(+21.98)PAPEP**	47,87	1141,527	11	-0,6	571,7703	31,88	0	1	10715	150211-Fr_I_Helo-red-alk-tryp-001.raw	8	8	JAW07022.1:P86821.1	Sodium adduct	E6:Sodium adduct:69.81
**AEIDF(+31.99)SGIPEDIIK**	47,84	1577,782	14	-0,4	789,8981	56,7	0	1	19401	150211-Fr_I_Helo-red-alk-tryp-001.raw	1	1	JAW06987.1:JAW07038.1:JAW06992.1:P84190.1	Dihydroxy	F5:Dihydroxy:42.68
**AEIDFSGIPEDIIK(+14.02)QIK(+43.01)**	47,82	1972,052	17	1,4	658,3588	47,26	7,40E+08	1	16254	150211-Fr_I_Helo-red-alk-tryp-001.raw	7	7	JAW06987.1:JAW07038.1:JAW06992.1	Methylation(KR); Carbamylation	K14:Methylation(KR):0.00;K17:Carbamylation:0.00
**AQVIGITPFK**	47,78	1072,628	10	2,3	537,3225	32,93	0	1	11087	150211-Fr_I_Helo-red-alk-tryp-001.raw	2	2	AIJ02110.2:JAG85213.1		
**EALAPEPAPEPAPE(+17.03)PAPEA**	47,77	1898,926	19	-0,8	950,4695	21,04	3,43E+09	1	7049	150211-Fr_I_Helo-red-alk-tryp-001.raw	45	45	JAW07022.1:P86821.1	Replacement of proton with ammonium ion	E14:Replacement of proton with ammonium ion:9.95
**VDTVAHETAHLIGAPH(+72.02)DGEGPEETGLSGSPGAK**	47,62	3307,559	33	4,9	662,5223	18	0	1	5901	150211-Fr_I_Helo-red-alk-tryp-001.raw	1	1	JAG85213.1	Ethoxyformylation	H16:Ethoxyformylation:41.57
**LS(+60.00)SAANMMQMEWDDELAAVAQAHANQC(+57.02)K**	47,5	3179,36	28	3,6	1060,798	43,27	0	1	14844	150211-Fr_I_Helo-red-alk-tryp-001.raw	1	1	JAW07031.1:JAW06995.1	2-OH-ethyl thio-Ser; Carbamidomethylation	S2:2-OH-ethyl thio-Ser:0.00;C27:Carbamidomethylation:1000.00
**APEAAPEAAPE(+37.96)PAAAAPE**	47,46	1696,735	18	-2,2	849,3727	32,03	0	1	10769	150211-Fr_I_Helo-red-alk-tryp-001.raw	1	1	JAW07022.1:P86821.1	Replacement of proton by potassium	E11:Replacement of proton by potassium:52.34
**AEIDFSGIP(-30.01)EDIIK**	47,41	1515,782	14	0,3	758,8985	38,2	0	1	13011	150211-Fr_I_Helo-red-alk-tryp-001.raw	7	7	JAW06987.1:JAW07038.1:JAW06992.1:P84190.1	Proline oxidation to pyrrolidinone	P9:Proline oxidation to pyrrolidinone:1000.00
**AEIDFSGIPED(+37.96)IIKQ**	47,22	1711,807	15	-3,3	856,908	42,68	0	1	14632	150211-Fr_I_Helo-red-alk-tryp-001.raw	1	1	JAW06987.1:JAW07038.1:JAW06992.1	Replacement of proton by potassium	D11:Replacement of proton by potassium:0.00
**GSSGYC(+57.02)AWPAC(+57.02)YC(+57.02)YGLPDS(+59.02)VKIWTSETNK**	47,09	3415,477	29	1,6	1139,502	49,38	0	1	16981	150211-Fr_I_Helo-red-alk-tryp-001.raw	1	1	AAC25688.1:O77463.1:AAC25689.1	Carbamidomethylation; Aminoethylcysteine	C6:Carbamidomethylation:1000.00;C11:Carbamidomethylation:1000.00;C13:Carbamidomethylation:1000.00;S19:Aminoethylcysteine:7.64
**A(+119.04)EIDFSGIPEDIIK**	46,81	1664,83	14	-2,7	833,4199	45,36	0	1	15589	150211-Fr_I_Helo-red-alk-tryp-001.raw	3	3	JAW06987.1:JAW07038.1:JAW06992.1:P84190.1	Pyridylacetyl	A1:Pyridylacetyl:93.42
**AEIDFS(+136.03)GIPEDIIK**	46,8	1681,822	14	-0,7	841,9175	46,16	0	1	15870	150211-Fr_I_Helo-red-alk-tryp-001.raw	2	2	JAW06987.1:JAW07038.1:JAW06992.1:P84190.1	O-Diethylphosphorylation	S6:O-Diethylphosphorylation:142.27
**MGDPREDGT(+59.02)VDINTAGIANSAGVC(+57.02)KPC(+57.02)FK**	46,79	3138,436	29	1,2	785,6171	35,87	0	1	12148	150211-Fr_I_Helo-red-alk-tryp-001.raw	1	1	AIJ02109.2:P86392.3:V9Z9A3.1:AHE40589.1:AHE40588.1	Aminoethylcysteine; Carbamidomethylation	T9:Aminoethylcysteine:39.44;C24:Carbamidomethylation:1000.00;C27:Carbamidomethylation:1000.00
**RAEIDFSGIPEDIIK**	46,72	1701,894	15	-0,5	568,3049	36,77	0	1	12479	150211-Fr_I_Helo-red-alk-tryp-001.raw	2	2	JAW06987.1:JAW07038.1:JAW06992.1		

**PAGFC(+57.02)DGYC(+57.02)K**	46,48	1173,458	10	-0,5	587,7361	25,52	0	1	8627	150211-Fr_I_Helo-red-alk-tryp-001.raw	8	8	JAW07016.1:JAW06979.1:P68411.1:1913435A:P68410.1:AAB36322.1	Carbamidomethylation	C5:Carbamidomethylation:1000.00;C9:Carbamidomethylation:1000.00
**KTQFGC(+57.02)PAYEGYC(+57.02)N(+.98)DHC(+57.02)NDIERK**	46,38	2862,179	23	4,7	573,4459	31,33	0	1	10524	150211-Fr_I_Helo-red-alk-tryp-001.raw	1	1	P0C2F3.1:P69940.2	Carbamidomethylation; Deamidation (NQ)	C6:Carbamidomethylation:1000.00;C13:Carbamidomethylation:1000.00;N14:Deamidation (NQ):31.56;C17:Carbamidomethylation:1000.00
**YFFTLPQASC(+57.02)LREDC(+57.02)PN(+.98)SG**	46,33	2261,972	19	0,8	1131,994	43,31	0	1	14858	150211-Fr_I_Helo-red-alk-tryp-001.raw	1	1	JAW07039.1	Carbamidomethylation; Deamidation (NQ)	C10:Carbamidomethylation:1000.00;C15:Carbamidomethylation:1000.00;N17:Deamidation (NQ):55.44
**LTS(+58.03)QSEYAC(+57.02)PAIDKFC(+57.02)EDHC(+57.02)AA**	46,3	2630,096	22	-2,6	877,7037	34,45	0	1	11641	150211-Fr_I_Helo-red-alk-tryp-001.raw	1	1	P86822.2:P0C8W4.2:AFP87551.1	2 3-dihydro-2 2-dimethyl-7-benzofuranol N-methyl carbamate; Carbamidomethylation	S3:2 3-dihydro-2 2-dimethyl-7-benzofuranol N-methyl carbamate:14.04;C9:Carbamidomethylation:1000.00;C16:Carbamidomethylation:1000.00;C20:Carbamidomethylation:1000.00
**YLFLTDSNFR**	46,26	1274,63	10	-0,7	638,3216	34,27	1,61E+08	1	11574	150211-Fr_I_Helo-red-alk-tryp-001.raw	69	69	AIJ02112.2		
**VTES(+58.03)DKQTILETHNQLR**	46,2	2069,063	17	-3,1	690,6927	30,15	0	1	10120	150211-Fr_I_Helo-red-alk-tryp-001.raw	3	3	JAW07031.1:JAW06995.1	2 3-dihydro-2 2-dimethyl-7-benzofuranol N-methyl carbamate	S4:2 3-dihydro-2 2-dimethyl-7-benzofuranol N-methyl carbamate:1000.00
**APEAAPE(+21.98)PAAAAPE**	46,07	1312,591	14	-1,8	657,3016	6,07	2,99E+08	1	1554	150211-Fr_I_Helo-red-alk-tryp-001.raw	56	56	JAW07022.1:P86821.1:P0C8W6.1	Sodium adduct	E7:Sodium adduct:56.15
**GSSGYC(+57.02)AWPAC(+57.02)YC(+57.02)YGLPD(+17.03)SVK**	45,93	2414,013	21	-0,9	805,6776	42,82	0	1	14680	150211-Fr_I_Helo-red-alk-tryp-001.raw	6	6	AAC25688.1:O77463.1:AAC25689.1	Carbamidomethylation; Replacement of proton with ammonium ion	C6:Carbamidomethylation:1000.00;C11:Carbamidomethylation:1000.00;C13:Carbamidomethylation:1000.00;D18:Replacement of proton with ammonium ion:10.22
**EALAPEPAPEPAPEPAPEAAPEAAPEPAAAAPE**	45,91	3154,498	33	1	631,9075	40,9	0	1	13987	150211-Fr_I_Helo-red-alk-tryp-001.raw	3	3	JAW07022.1:P86821.1		
**EALAPEPAPEPAPEPAPEAAPE(+17.03)AAPEPAAAAPE**	45,83	3171,525	33	3,5	1058,186	44,1	0	1	15141	150211-Fr_I_Helo-red-alk-tryp-001.raw	1	1	JAW07022.1:P86821.1	Replacement of proton with ammonium ion	E22:Replacement of proton with ammonium ion:0.00
**TITAVEIPDPFWVDAIR**	45,74	1942,02	17	2,9	972,0201	52,98	0	1	18178	150211-Fr_I_Helo-red-alk-tryp-001.raw	1	1	JAW06991.1:JAW07032.1:JAG85159.1		
**ESLIPGEEVFD(+21.98)FGHVVGN(+.98)LNVLNC(+57.02)K**	45,73	2808,34	25	4,2	703,0953	52,44	0	1	18003	150211-Fr_I_Helo-red-alk-tryp-001.raw	1	1	AIJ02112.2	Sodium adduct; Deamidation (NQ); Carbamidomethylation	D11:Sodium adduct:0.00;N18:Deamidation (NQ):22.85;C24:Carbamidomethylation:1000.00
**WM(sub L)PLSGPVSGILPHEGK**	45,67	1803,934	17	2	602,3198	37,75	0	1	12841	150211-Fr_I_Helo-red-alk-tryp-001.raw	1	1	XP_023242894.1		
**AEIDFSGIPE(+53.92)DIIK**	45,59	1599,712	14	-2,1	534,2435	38,37	0	1	13072	150211-Fr_I_Helo-red-alk-tryp-001.raw	8	8	JAW06987.1:JAW07038.1:JAW06992.1:P84190.1	Replacement of 2 protons by iron	E10:Replacement of 2 protons by iron:12.28

**Q(sub A)DVDFTGIADSIIK**	45,55	1520,772	14	-0,4	761,3931	45,02	3,12E+09	1	15469	150211-Fr_I_Helo-red-alk-tryp-001.raw	44	44	JAW07002.1:P86824.1		
**ESLIPGEER(sub V)FDFGHVVGNLNVLNC(+57.02)K**	45,52	2842,407	25	-3,3	948,4733	53,33	0	1	18292	150211-Fr_I_Helo-red-alk-tryp-001.raw	2	2	AIJ02112.2	Carbamidomethylation	C24:Carbamidomethylation:1000.00
**G(sub A)DVDFTGIADSIIK**	45,51	1449,735	14	-0,9	725,8742	43,05	0	1	14764	150211-Fr_I_Helo-red-alk-tryp-001.raw	4	4	JAW07002.1:P86824.1		
**KQNEPSFIEDS(+58.03)AIPGHQQVLDPVDLVK**	45,5	3060,564	27	-2,8	1021,192	43,62	0	1	14966	150211-Fr_I_Helo-red-alk-tryp-001.raw	1	1	AIJ02110.2	2 3-dihydro-2 2-dimethyl-7-benzofuranol N-methyl carbamate	S11:2 3-dihydro-2 2-dimethyl-7-benzofuranol N-methyl carbamate:0.00
**SPC(+57.02)IIIDYLC(+57.02)VTE(+356.19)TTF**	45,41	2287,094	16	-2,2	763,3702	51,23	0	1	17601	150211-Fr_I_Helo-red-alk-tryp-001.raw	1	1	AIJ02110.2	Carbamidomethylation; Biotin polyethyleneoxide amine	C3:Carbamidomethylation:1000.00;C10:Carbamidomethylation:1000.00;E13:Biotin polyethyleneoxide amine:109.28
**QTILETHNQLR**	45,34	1351,721	11	-0,9	451,5805	5,93	0	1	1502	150211-Fr_I_Helo-red-alk-tryp-001.raw	8	8	JAW07031.1:JAW06995.1		
**PEPAPEPAPEAAPEAAPEPAAAAPER(+290.18)**	45,3	2822,39	26	0,4	941,8044	37,92	0	1	12905	150211-Fr_I_Helo-red-alk-tryp-001.raw	1	1	JAW07022.1	Levuglandinyl - arginine lactam adduct	R26:Levuglandinyl - arginine lactam adduct:1000.00
**NVLVIAPPDPSVFSDPDRDIELA**	45,18	2478,264	23	-0,1	827,0952	51,86	4,67E+07	1	17811	150211-Fr_I_Helo-red-alk-tryp-001.raw	1	1	AIJ02118.2		
**FGPD(sub N)VAGETAASEVNFHFSAPHLIFPEGMK**	45,12	3201,523	30	5,3	641,3152	51,07	0	1	17548	150211-Fr_I_Helo-red-alk-tryp-001.raw	2	2	JAW07031.1:JAW06995.1		
**APEPAPEPAPEPAPEAAPEAAPEPAAAAPE(+37.03)RR**	45,1	3190,568	32	-2,8	1064,527	40,7	0	1	13913	150211-Fr_I_Helo-red-alk-tryp-001.raw	3	3	JAW07022.1	Propargylamine	E30:Propargylamine:0.00
**SITPDGPQIR**	44,96	1082,572	10	-0,1	542,2932	29,93	3,84E+07	1	10051	150211-Fr_I_Helo-red-alk-tryp-001.raw	105	105	JAW07031.1:JAW06995.1		
**AEIDFSGIPEDIIKEIKE(+43.99)**	44,87	2089,047	18	1	1045,532	49,51	0	1	17027	150211-Fr_I_Helo-red-alk-tryp-001.raw	1	1	P84190.1	Carboxylation (E)	E18:Carboxylation (E):0.00
**ESLIPGEEVFDFGHVVGNL**	44,83	2057,011	19	-0,4	1029,512	52,18	3,88E+08	1	17915	150211-Fr_I_Helo-red-alk-tryp-001.raw	2	2	AIJ02112.2		
**EID(-18.01)FSGIPEDIIK**	44,77	1456,745	13	0,7	729,3802	51,07	0	1	17547	150211-Fr_I_Helo-red-alk-tryp-001.raw	2	2	JAW06987.1:JAW07038.1:JAW06992.1:P84190.1	Dehydration	D3:Dehydration:39.76
**IWTSETNK**	44,76	977,4818	8	-0,8	489,7478	16,29	4,90E+08	1	5254	150211-Fr_I_Helo-red-alk-tryp-001.raw	257	257	AAC25688.1:O77463.1:AAC25689.1		
**C(+57.02)YGLPDSVK**	44,65	1037,485	9	0,3	519,75	30,6	0	1	10274	150211-Fr_I_Helo-red-alk-tryp-001.raw	3	3	AAC25688.1:O77463.1:AAC25689.1	Carbamidomethylation	C1:Carbamidomethylation:1000.00
**F(+43.01)FLNK(+14.02)DEASC(+57.02)VVSAC(+57.02)K**	44,62	1930,892	16	2	966,4551	17,15	0	1	5574	150211-Fr_I_Helo-red-alk-tryp-001.raw	1	1	AIJ02112.2	Carbamylation; Methylation(KR); Carbamidomethylation	F1:Carbamylation:65.28;K5:Methylation(KR):69.77;C10:Carbamidomethylation:1000.00;C15:Carbamidomethylation:1000.00
**Q(sub A)EIDFSGIPEDIIK**	44,48	1602,814	14	-0,3	802,4141	42,09	1,23E+10	1	14418	150211-Fr_I_Helo-red-alk-tryp-001.raw	576	576	JAW06987.1:JAW07038.1:JAW06992.1:P84190.1		
**ADIIMLIVSR**	44,41	1129,653	10	1	565,8342	35,95	0	1	12176	150211-Fr_I_Helo-red-alk-tryp-001.raw	2	2	JAW07039.1:JAG85190.1		

**AEIDFSGIPE(+37.96)DIIKEI**	44,38	1825,875	16	-1,1	913,9438	51,02	0	1	17530	150211-Fr_I_Helo-red-alk-tryp-001.raw	1	1	P84190.1	Replacement of proton by potassium	E10:Replacement of proton by potassium:0.00
**AEIDFSGIPEDIIG(sub K)QIK**	44,32	1843,957	17	-0,3	922,9853	50,22	2,98E+08	1	17265	150211-Fr_I_Helo-red-alk-tryp-001.raw	4	4	JAW06987.1:JAW07038.1:JAW06992.1		
**ET(-18.01)NAKPPA**	44,31	808,4079	8	0,8	405,2115	74,97	0	1	25270	150211-Fr_I_Helo-red-alk-tryp-001.raw	1	1	JAW06987.1:JAW07038.1:JAW06992.1:P84190.1:JAW07002.1	Dehydration	T2:Dehydration:1000.00
**I(+43.01)DKPLILHPFAFR**	44,19	1608,914	13	-0,4	537,3117	34,02	0	1	11486	150211-Fr_I_Helo-red-alk-tryp-001.raw	4	4	XP_023239442.1	Carbamylation	I1:Carbamylation:0.00
**VGVAQDDSDYNERVD**	44,16	1680,723	15	-0,9	841,3679	29,83	0	1	10020	150211-Fr_I_Helo-red-alk-tryp-001.raw	3	3	AIJ02110.2:JAG85213.1		
**AEIDFSGIPED(+21.98)IIK**	43,98	1567,775	14	-0,3	784,8943	72,94	6,55E+06	1	24621	150211-Fr_I_Helo-red-alk-tryp-001.raw	28	28	JAW06987.1:JAW07038.1:JAW06992.1:P84190.1	Sodium adduct	D11:Sodium adduct:0.00
**H(+163.05)QIGDYTHVEQNFAVATYSIGTEIKPSQR**	43,9	3451,662	29	-3,1	691,3375	37,72	0	1	12828	150211-Fr_I_Helo-red-alk-tryp-001.raw	1	1	JAW07036.1:JAW06986.1	Phenethyl isothiocyanate	H1:Phenethyl isothiocyanate:84.74
**GGREGYPADSK**	43,71	1135,526	11	0,4	568,7704	6,06	4,36E+07	1	1548	150211-Fr_I_Helo-red-alk-tryp-001.raw	66	66	AAC25688.1:O77463.1:AAC25689.1		
**ITC(+57.02)FLTAAGYC(+57.02)N(+.98)TEC(+57.02)TLKK**	43,56	2251,032	19	4,5	1126,528	18,14	0	1	5953	150211-Fr_I_Helo-red-alk-tryp-001.raw	1	1	AAC25688.1:O77463.1:AAC25689.1	Carbamidomethylation; Deamidation (NQ)	C3:Carbamidomethylation:1000.00;C11:Carbamidomethylation:1000.00;N12:Deamidation (NQ):1000.00;C15:Carbamidomethylation:1000.00
**LKEALAPEPAPEPAPEP**	43,55	1754,909	17	3,8	878,4651	16,9	0	1	5482	150211-Fr_I_Helo-red-alk-tryp-001.raw	1	1	JAW07022.1:P86821.1		
**AEIDFSGIPEDIIK(+58.01)QIK**	43,54	1973,036	17	1,2	658,6866	48,59	0	1	16707	150211-Fr_I_Helo-red-alk-tryp-001.raw	2	2	JAW06987.1:JAW07038.1:JAW06992.1	Carboxymethyl (KW X@N-term)	K14:Carboxymethyl (KW X@N-term):11.12
**PEPAPEPAPEAAPEAAPEPAAAAP(+31.99)ERRR**	43,47	2876,405	28	-5,1	959,8041	36,79	0	1	12487	150211-Fr_I_Helo-red-alk-tryp-001.raw	1	1	JAW07022.1	Dihydroxy	P24:Dihydroxy:0.00
**NM(sub E)PSFIEDSAIPGHQQVLDPVDLVK**	43,47	2748,379	25	1,6	917,1351	46,01	0	1	15816	150211-Fr_I_Helo-red-alk-tryp-001.raw	1	1	AIJ02110.2		
**DFSGIPEDIIK**	43,37	1232,629	11	-0,5	617,3214	31,14	0	1	10460	150211-Fr_I_Helo-red-alk-tryp-001.raw	6	6	JAW06987.1:JAW07038.1:JAW06992.1:P84190.1		
**LAPEPAPEPAPEPAPEAAPEAAPEPAAAAPE**	43,32	2954,419	31	0,7	985,8141	39	0	1	13296	150211-Fr_I_Helo-red-alk-tryp-001.raw	1	1	JAW07022.1:P86821.1		
**VWDYATNK**	43,31	995,4713	8	-0,1	498,7429	7,95	1,43E+08	1	2233	150211-Fr_I_Helo-red-alk-tryp-001.raw	113	113	JAW07016.1:JAW06979.1:P68411.1:1913435A:P68410.1:AAB36322.1:C0HJM9.1:JAG85185.1:CEF39517.1:P60276.1		
**ADVDFTGIADSIIF(sub K)K**	43,17	1610,819	15	-1,3	806,4158	50,39	0	1	17321	150211-Fr_I_Helo-red-alk-tryp-001.raw	2	2	JAW07002.1		
**G(+42.01)SSGYC(+57.02)AW(+15.99)PAC(+57.02)YC(+57.02)YGLPDSVK**	43,07	2454,992	21	4,1	819,3412	45,52	0	1	15644	150211-Fr_I_Helo-red-alk-tryp-001.raw	2	2	AAC25688.1:O77463.1:AAC25689.1	Acetylation (N-term); Carbamidomethylation; Oxidation (HW)	G1:Acetylation (N-term):1000.00;C6:Carbamidomethylation:1000.00;W8:Oxidation (HW):1000.00;C11:Carbamidomethylation:1000.00;C13:Carbamidomethylation:1000.00

**EALAPEPAPEPAPE(+53.92)PAPEA**	43,03	1935,819	19	-3,5	968,9133	35,36	0	1	11966	150211-Fr_I_Helo-red-alk-tryp-001.raw	1	1	JAW07022.1:P86821.1	Replacement of 2 protons by iron	E14:Replacement of 2 protons by iron:0.00
**KEALAPEPAPEP**	42,94	1247,64	12	-0,3	624,8269	23,3	1,37E+09	1	7867	150211-Fr_I_Helo-red-alk-tryp-001.raw	153	153	JAW07022.1:P86821.1		
**AEIDFSGIPEDIIKEIKE(-.98)**	42,81	2044,073	18	1,4	682,3658	38,22	0	1	13018	150211-Fr_I_Helo-red-alk-tryp-001.raw	1	1	P84190.1	Amidation	E18:Amidation:1000.00
**RLTS(+58.03)QSEYAC(+57.02)PAIDKFC(+57.02)EDHC(+57.02)AAK**	42,77	2914,292	24	-2,1	729,5788	32,4	0	1	10900	150211-Fr_I_Helo-red-alk-tryp-001.raw	1	1	P86822.2:P0C8W4.2:AFP87551.1	2 3-dihydro-2 2-dimethyl-7-benzofuranol N-methyl carbamate; Carbamidomethylation	S4:2 3-dihydro-2 2-dimethyl-7-benzofuranol N-methyl carbamate:0.00;C10:Carbamidomethylation:1000.00;C17:Carbamidomethylation:1000.00;C21:Carbamidomethylation:1000.00
**A(+43.01)SSGYC(+57.02)AWPAC(+57.02)YC(+57.02)YGVPSN(+.98)IK**	42,7	2454,008	21	-1,9	819,0083	41,88	9,86E+08	1	14340	150211-Fr_I_Helo-red-alk-tryp-001.raw	2	2	C0HJM9.1:JAG85185.1:CEF39517.1:P60276.1	Carbamylation; Carbamidomethylation; Deamidation (NQ)	A1:Carbamylation:76.28;C6:Carbamidomethylation:1000.00;C11:Carbamidomethylation:1000.00;C13:Carbamidomethylation:1000.00;N19:Deamidation (NQ):1000.00
**RLTSQSEYAC(+57.02)PAIDK**	42,62	1737,836	15	-1	869,9242	30,28	0	1	10165	150211-Fr_I_Helo-red-alk-tryp-001.raw	2	2	P86822.2:P0C8W4.2:AFP87551.1	Carbamidomethylation	C10:Carbamidomethylation:1000.00
**FFTLPQASC(+57.02)LR**	42,6	1338,675	11	0,2	670,3451	34,05	0	1	11497	150211-Fr_I_Helo-red-alk-tryp-001.raw	1	1	JAW07039.1	Carbamidomethylation	C9:Carbamidomethylation:1000.00
**T(sub V)N(sub D)TVAHETAHLIGAPHDEEGPEQTGISGSPGAK**	42,56	3307,571	33	1,9	1103,533	15,64	0	1	5013	150211-Fr_I_Helo-red-alk-tryp-001.raw	2	2	AIJ02110.2		
**AEIDFSGIPEDIIK(+28.03)Q**	42,29	1701,882	15	4,9	851,9527	37,05	6,68E+08	1	12585	150211-Fr_I_Helo-red-alk-tryp-001.raw	1	1	JAW06987.1:JAW07038.1:JAW06992.1	Dimethylation(KR)	K14:Dimethylation(KR):1000.00
**TEPSFIKDNLIPGPPAAFDPDVLISAM(+15.99)SK(-.98)(+42.01)**	42,28	3126,595	29	-0,3	1043,205	50,89	1,69E+09	1	17486	150211-Fr_I_Helo-red-alk-tryp-001.raw	2	2	AIJ02109.2:P86392.3:V9Z9A3.1:AHE40589.1:AHE40588.1	Oxidation (M); Amidation; Acetylation (K)	M27:Oxidation (M):1000.00;K29:Amidation:1000.00;K29:Acetylation (K):22.04
**ASSGYC(+57.02)AWPAC(+57.02)YC(+57.02)YGVPDHIK(+114.04)**	42	2575,072	21	-1,8	859,363	37,08	0	1	12596	150211-Fr_I_Helo-red-alk-tryp-001.raw	2	2	JAW07016.1:JAW06979.1:P68411.1:1913435A:P68410.1:AAB36322.1	Carbamidomethylation; Ubiquitin	C6:Carbamidomethylation:1000.00;C11:Carbamidomethylation:1000.00;C13:Carbamidomethylation:1000.00;K21:Ubiquitin:47.57
**APEAAPEPAAAAPE**	42	1290,609	14	-0,7	646,3114	19,41	3,13E+09	1	6436	150211-Fr_I_Helo-red-alk-tryp-001.raw	241	241	JAW07022.1:P86821.1:P0C8W6.1		
**AEIDFSGIPEDIIKR(sub E)**	41,83	1701,894	15	0,2	568,3053	37,12	1,85E+09	1	12611	150211-Fr_I_Helo-red-alk-tryp-001.raw	4	4	P84190.1		
**N(sub G)APAQYLDISYIR**	41,72	1522,778	13	0,7	762,3968	33,61	0	1	11335	150211-Fr_I_Helo-red-alk-tryp-001.raw	1	1	JAW06991.1:JAW07032.1:JAG85159.1		
**AEID(+53.92)FSGIPEDIIK**	41,7	1599,712	14	-0,4	534,2444	38,58	0	1	13148	150211-Fr_I_Helo-red-alk-tryp-001.raw	19	19	JAW06987.1:JAW07038.1:JAW06992.1:P84190.1	Replacement of 2 protons by iron	D4:Replacement of 2 protons by iron:0.00

**AEIDFSGIPEDIIK(+114.04)**	41,65	1659,835	14	-2,1	830,9232	42,79	0	1	14669	150211-Fr_I_Helo-red-alk-tryp-001.raw	19	19	JAW06987.1:JAW07038.1:JAW06992.1:P84190.1	Ubiquitin	K14:Ubiquitin:26.68
**KVLLISFDGFR**	41,49	1293,745	11	1,3	647,8804	36,06	0	1	12219	150211-Fr_I_Helo-red-alk-tryp-001.raw	1	1	XP_023242894.1:XP_023242898.1		
**KGS(+59.02)SGYC(+57.02)AWPAC(+57.02)YC(+57.02)YGLPDSVK**	41,49	2584,101	22	1	862,375	38	0	1	12937	150211-Fr_I_Helo-red-alk-tryp-001.raw	3	3	AAC25688.1:O77463.1:AAC25689.1	Aminoethylcysteine; Carbamidomethylation	S3:Aminoethylcysteine:0.00;C7:Carbamidomethylation:1000.00;C12:Carbamidomethylation:1000.00;C14:Carbamidomethylation:1000.00
**EPAPEPAPEAAPEAAPEPAAAAPE**	41,42	2279,059	24	-3,2	1140,533	15,97	0	1	5132	150211-Fr_I_Helo-red-alk-tryp-001.raw	5	5	JAW07022.1:P86821.1		
**KGSSGYC(+57.02)AWPAC(+57.02)**	41,41	1342,544	12	-0,2	672,2789	31,04	0	1	10425	150211-Fr_I_Helo-red-alk-tryp-001.raw	1	1	AAC25688.1:O77463.1:AAC25689.1:AAW72453.1:Q5G8B8.1:AAB29128.1:P56612.1:AIA81145.1:CAA46982.1:P15226.2:P0DMM8.1:1NPI:1B7D:AAB36321.1:AAB36318.1:P56611.1:JAG85191.1:JAG85210.1	Carbamidomethylation	C7:Carbamidomethylation:1000.00;C12:Carbamidomethylation:1000.00
**A(+380.15)C(+57.02)PAIDKFC(+57.02)EDHC(+57.02)AAK**	41,32	2271,949	16	-3,4	758,3209	31,74	0	1	10665	150211-Fr_I_Helo-red-alk-tryp-001.raw	3	3	P86822.2:P0C8W4.2:AFP87551.1:Q0GY42.1:ABE98265.1:JAG85223.1	Nucleophilic addition to cytopiloyne+H2O; Carbamidomethylation	A1:Nucleophilic addition to cytopiloyne+H2O:90.57;C2:Carbamidomethylation:1000.00;C9:Carbamidomethylation:1000.00;C13:Carbamidomethylation:1000.00
**APEPAPEAAPEAAPEPAA**	41,19	1684,794	18	-0,9	843,4037	14,3	0	1	4521	150211-Fr_I_Helo-red-alk-tryp-001.raw	13	13	JAW07022.1:P86821.1		
**LAPEPAPEPAPEPAPEA**	40,92	1681,82	17	-0,8	841,9165	17,89	2,71E+08	1	5856	150211-Fr_I_Helo-red-alk-tryp-001.raw	26	26	JAW07022.1:P86821.1		
**S(+58.03)LFVGEDVFNMELIAK**	40,89	1868,947	16	-3	935,4778	50,9	0	1	17490	150211-Fr_I_Helo-red-alk-tryp-001.raw	2	2	AMO02558.1:JAW07025.1	2 3-dihydro-2 2-dimethyl-7-benzofuranol N-methyl carbamate	S1:2 3-dihydro-2 2-dimethyl-7-benzofuranol N-methyl carbamate:1000.00
**LIGIQAFTK**	40,71	989,5909	9	-0,1	495,8027	32,56	0	1	10956	150211-Fr_I_Helo-red-alk-tryp-001.raw	32	32	JAW07039.1:JAG85190.1:JAW07042.1		
**FINGGTDNSGIIITLLPGS(sub T)DS(sub P)SVTKR**	40,71	2661,397	26	-1,4	888,1384	44,72	0	1	15361	150211-Fr_I_Helo-red-alk-tryp-001.raw	2	2	XP_023239442.1		
**APEAAPEAAPEPAAAAP(-27.99)ER**	40,63	1786,885	19	-1,4	894,4484	30,99	0	1	10408	150211-Fr_I_Helo-red-alk-tryp-001.raw	1	1	JAW07022.1	Pyrrolidone from Proline	P17:Pyrrolidone from Proline:22.07
**KEALAPEPAPEPAPE(+37.96)PAPEA**	40,58	2047,95	20	-2,4	1024,98	33,54	0	1	11307	150211-Fr_I_Helo-red-alk-tryp-001.raw	1	1	JAW07022.1:P86821.1	Replacement of proton by potassium	E15:Replacement of proton by potassium:9.81
**PEAAPEPAAAAPERR**	40,53	1531,774	15	-1,8	766,8929	39,39	0	1	13439	150211-Fr_I_Helo-red-alk-tryp-001.raw	3	3	JAW07022.1		
**VTESDKQ(+.98)TILETHNQLR**	40,45	2012,018	17	4,3	671,6827	27,72	0	1	9333	150211-Fr_I_Helo-red-alk-tryp-001.raw	2	2	JAW07031.1:JAW06995.1	Deamidation (NQ)	Q7:Deamidation (NQ):79.54

**LAPEPAPEPAPEPAPEAAPEAAPE(+17.03)PAAAAPE**	40,39	2971,445	31	0,7	991,4896	39	0	1	13298	150211-Fr_I_Helo-red-alk-tryp-001.raw	1	1	JAW07022.1:P86821.1	Replacement of proton with ammonium ion	E24:Replacement of proton with ammonium ion:8.82
**LTS(+58.03)QSEYAC(+57.02)PAIDK**	40,37	1639,764	14	-1,5	820,8879	7,79	0	1	2176	150211-Fr_I_Helo-red-alk-tryp-001.raw	8	8	P86822.2:P0C8W4.2:AFP87551.1	2 3-dihydro-2 2-dimethyl-7-benzofuranol N-methyl carbamate; Carbamidomethylation	S3:2 3-dihydro-2 2-dimethyl-7-benzofuranol N-methyl carbamate:26.31;C9:Carbamidomethylation:1000.00
**SEYVSAPL(sub T)C(+57.02)AQ(sub S)GSQVIYAWAR**	40,3	2355,132	21	-0,7	1178,572	44,12	0	1	15149	150211-Fr_I_Helo-red-alk-tryp-001.raw	1	1	XP_023239442.1	Carbamidomethylation	C9:Carbamidomethylation:1000.00
**PEPAPEPAPEAAPEAAPEPAAAAPER(+199.07)RR**	40,27	3043,482	28	-0,1	1015,501	40,61	0	1	13879	150211-Fr_I_Helo-red-alk-tryp-001.raw	3	3	JAW07022.1	Oxidized Arginine biotinylated with biotin hydrazide	R26:Oxidized Arginine biotinylated with biotin hydrazide:0.00
**EALAPE(+37.96)PAPEP**	40,26	1157,501	11	-2,7	579,756	31,92	0	1	10728	150211-Fr_I_Helo-red-alk-tryp-001.raw	2	2	JAW07022.1:P86821.1	Replacement of proton by potassium	E6:Replacement of proton by potassium:59.24
**LATDDQC(+57.02)IVIEILS(+58.03)VTDKLVTK**	40,23	2531,364	22	-4,1	844,7919	55,27	0	1	18926	150211-Fr_I_Helo-red-alk-tryp-001.raw	1	1	AIJ02113.2	Carbamidomethylation; 2 3-dihydro-2 2-dimethyl-7-benzofuranol N-methyl carbamate	C7:Carbamidomethylation:1000.00;S14:2 3-dihydro-2 2-dimethyl-7-benzofuranol N-methyl carbamate:1000.00
**GSSGYC(+57.02)AWPAC(+57.02)YC(+57.02)YGLPD**	39,95	2082,791	18	-1,5	1042,401	44,64	0	1	15332	150211-Fr_I_Helo-red-alk-tryp-001.raw	2	2	AAC25688.1:O77463.1:AAC25689.1	Carbamidomethylation	C6:Carbamidomethylation:1000.00;C11:Carbamidomethylation:1000.00;C13:Carbamidomethylation:1000.00
**QIKETN(+.98)AKPPA**	39,9	1196,64	11	-3	599,3255	9,57	0	1	2825	150211-Fr_I_Helo-red-alk-tryp-001.raw	47	47	JAW06987.1:JAW07038.1:JAW06992.1	Deamidation (NQ)	N6:Deamidation (NQ):21.59
**ADVDFTGIADSIIL(sub K)K**	39,89	1576,835	15	-0,8	789,4241	49,69	0	1	17085	150211-Fr_I_Helo-red-alk-tryp-001.raw	12	12	JAW07002.1		
**AQVIGITPFKK**	39,8	1200,723	11	-0,1	601,3687	14,29	0	1	4518	150211-Fr_I_Helo-red-alk-tryp-001.raw	2	2	AIJ02110.2:JAG85213.1		
**AEF(ins)IDFSGIPEDIIK**	39,71	1692,861	15	0,1	847,4379	46,9	0	1	16126	150211-Fr_I_Helo-red-alk-tryp-001.raw	1	1	JAW06987.1:JAW07038.1:JAW06992.1		
**AEIDFSGIPEDIIK(+70.04)QIK**	39,67	1985,072	17	-0,3	662,6978	47,16	0	1	16218	150211-Fr_I_Helo-red-alk-tryp-001.raw	1	1	JAW06987.1:JAW07038.1:JAW06992.1	Crotonaldehyde	K14:Crotonaldehyde:5.08
**AEIDFSGIPEDIIKR(sub Q)**	39,65	1701,894	15	-0,4	851,9538	37,23	6,68E+08	1	12650	150211-Fr_I_Helo-red-alk-tryp-001.raw	1	1			
**EIDFS(+150.04)GIPEDIIK**	39,44	1624,797	13	-1,7	542,6053	41,33	2,18E+08	1	14142	150211-Fr_I_Helo-red-alk-tryp-001.raw	29	29	JAW06987.1:JAW07038.1:JAW06992.1:P84190.1	S-guanylation-2	S5:S-guanylation-2:1000.00
**EGYPADSK**	39,36	865,3817	8	1,7	433,6989	5,4	2,49E+07	1	1328	150211-Fr_I_Helo-red-alk-tryp-001.raw	10	10	AAC25688.1:O77463.1:AAC25689.1:AAW72463.1:Q5G8A8.1		
**S(+59.02)SGYC(+57.02)AWPAC(+57.02)YC(+57.02)YGLPDSVK**	39,32	2398,984	20	1,2	1200,501	48,27	0	1	16599	150211-Fr_I_Helo-red-alk-tryp-001.raw	4	4	AAC25688.1:O77463.1:AAC25689.1	Aminoethylcysteine; Carbamidomethylation	S1:Aminoethylcysteine:0.00;C5:Carbamidomethylation:1000.00;C10:Carbamidomethylation:1000.00;C12:Carbamidomethylation:1000.00
**APEPAPEPAPEPAPEAAPEAAPEPAAAAP(+29.04)ER**	39,32	3026,475	31	-4,5	1009,828	39,14	0	1	13347	150211-Fr_I_Helo-red-alk-tryp-001.raw	2	2	JAW07022.1	Dimethylation of proline residue	P29:Dimethylation of proline residue:8.82
**AEIDFL(sub S)GIPEDIIK**	39,32	1571,845	14	0,1	786,9297	48,4	0	1	16645	150211-Fr_I_Helo-red-alk-tryp-001.raw	1	1	JAW06987.1:JAW07038.1:JAW06992.1		

**SC(+57.02)PYDDGYIMGS(+58.03)TIEPNMLK**	39,29	2348,025	20	-1,6	1175,018	20,82	0	1	6967	150211-Fr_I_Helo-red-alk-tryp-001.raw	8	8	JAG85190.1	Carbamidomethylation; 2 3-dihydro-2 2-dimethyl-7-benzofuranol N-methyl carbamate	C2:Carbamidomethylation:1000.00;S12:2 3-dihydro-2 2-dimethyl-7-benzofuranol N-methyl carbamate:0.00
**FSGIPEDIIK**	39,23	1117,602	10	-0,1	559,8082	33,88	0	1	11433	150211-Fr_I_Helo-red-alk-tryp-001.raw	19	19	JAW06987.1:JAW07038.1:JAW06992.1:P84190.1		
**L(ins)AEIDFSGIPEDIIK**	39,22	1658,877	15	-0,5	830,4451	45,6	0	1	15672	150211-Fr_I_Helo-red-alk-tryp-001.raw	1	1	P84190.1		
**IDKPLV(sub I)LHPFAFR**	39,19	1551,893	13	1	518,3053	33,99	0	1	11473	150211-Fr_I_Helo-red-alk-tryp-001.raw	11	11	XP_023239442.1		
**IDFSGIPEDIIKQ**	39,14	1473,772	13	0,5	737,8934	37,84	0	1	12873	150211-Fr_I_Helo-red-alk-tryp-001.raw	1	1	JAW06987.1:JAW07038.1:JAW06992.1		
**ITC(+57.02)FLTAAGYC(+57.02)NTE(+37.95)C(+57.02)TLK**	39,13	2159,9	18	0,2	540,9825	35,05	0	1	11853	150211-Fr_I_Helo-red-alk-tryp-001.raw	2	2	AAC25688.1:O77463.1:AAC25689.1	Carbamidomethylation; Replacement of 2 protons by calcium	C3:Carbamidomethylation:1000.00;C11:Carbamidomethylation:1000.00;E14:Replacement of 2 protons by calcium:7.28;C15:Carbamidomethylation:1000.00
**VLLISFDGFR**	39,05	1165,65	10	-2	583,8309	38,21	0	1	13015	150211-Fr_I_Helo-red-alk-tryp-001.raw	2	2	XP_023242894.1:XP_023242898.1		
**VLLLSFDGFR**	39,05	1165,65	10	-2	583,8309	38,21	0	1	13015	150211-Fr_I_Helo-red-alk-tryp-001.raw	2	2			
**ADVD(+53.92)FTGIADSIIK**	38,92	1517,67	14	1,7	506,8982	37,97	0	1	12925	150211-Fr_I_Helo-red-alk-tryp-001.raw	1	1	JAW07002.1:P86824.1	Replacement of 2 protons by iron	D4:Replacement of 2 protons by iron:12.81
**LMAETSIGVQNIIDTLDMGIK**	38,61	2261,165	21	1	754,7296	53,07	0	1	18208	150211-Fr_I_Helo-red-alk-tryp-001.raw	1	1			
**EAAPEPAAAAPE**	38,59	1122,519	12	-0,2	562,2668	29,69	1,26E+07	1	9972	150211-Fr_I_Helo-red-alk-tryp-001.raw	1	1	JAW07022.1:P86821.1:P0C8W6.1		
**AEIDFSGIPE(+21.98)D**	38,5	1213,512	11	-0,2	607,7629	35,73	0	1	12099	150211-Fr_I_Helo-red-alk-tryp-001.raw	11	11	JAW06987.1:JAW07038.1:JAW06992.1:P84190.1	Sodium adduct	E10:Sodium adduct:27.42
**ETNAKPPA**	38,47	826,4185	8	0	414,2165	11,43	4,91E+08	1	3492	150211-Fr_I_Helo-red-alk-tryp-001.raw	150	150	JAW06987.1:JAW07038.1:JAW06992.1:P84190.1:JAW07002.1		
**F(+58.01)GC(+57.02)PAYEGYC(+57.02)NDHC(+57.02)NDIER**	38,43	2433,905	19	5,2	1217,966	14,6	0	1	4631	150211-Fr_I_Helo-red-alk-tryp-001.raw	1	1	P0C2F3.1:P69940.2	Carboxymethyl (KW X@N-term); Carbamidomethylation	F1:Carboxymethyl (KW X@N-term):1000.00;C3:Carbamidomethylation:1000.00;C10:Carbamidomethylation:1000.00;C14:Carbamidomethylation:1000.00
**SSAAN(+228.11)MMQMEWDDELAAVAQAHANQC(+57.02)K**	38,29	3234,384	27	5	809,6072	33,77	0	1	11392	150211-Fr_I_Helo-red-alk-tryp-001.raw	1	1	JAW07031.1:JAW06995.1	2 4-diacetamido-2 4 6-trideoxyglucopyranose; Carbamidomethylation	N5:2 4-diacetamido-2 4 6-trideoxyglucopyranose:106.58;C26:Carbamidomethylation:1000.00
**PAPEPAPEPAPEAAPEAAPEPAAAAPER(+160.04)RR**	38,16	3172,542	30	-3,1	1058,518	40,36	0	1	13790	150211-Fr_I_Helo-red-alk-tryp-001.raw	2	2	JAW07022.1	Condensation product of glucosone	R28:Condensation product of glucosone:0.00
**ITC(+57.02)FLTAAGYC(+57.02)NTE**	38,14	1619,696	14	-1	810,8544	32,69	0	1	11003	150211-Fr_I_Helo-red-alk-tryp-001.raw	23	23	AAC25688.1:O77463.1:AAC25689.1	Carbamidomethylation	C3:Carbamidomethylation:1000.00;C11:Carbamidomethylation:1000.00

**AEIDFSGIPEDIIKL(sub E)IK**	38,07	1900,056	17	-0,2	951,0349	50,95	0	1	17507	150211-Fr_I_Helo-red-alk-tryp-001.raw	2	2	P84190.1		
**ITC(+57.02)FLTAAGYC(+57.02)N(+.98)TEC(+57.02)TLKKG**	38,03	2308,054	20	0,4	1155,035	18,26	0	1	5998	150211-Fr_I_Helo-red-alk-tryp-001.raw	1	1	AAC25688.1:O77463.1:AAC25689.1	Carbamidomethylation; Deamidation (NQ)	C3:Carbamidomethylation:1000.00;C11:Carbamidomethylation:1000.00;N12:Deamidation (NQ):1000.00;C15:Carbamidomethylation:1000.00
**LKEALAPEPAPEPAPEPAPEA**	37,99	2123,079	21	-1,6	708,699	18,72	0	1	6173	150211-Fr_I_Helo-red-alk-tryp-001.raw	1	1	JAW07022.1:P86821.1		
**APEAAPEPAAAAP(-27.99)ER**	37,93	1418,715	15	-0,1	710,3649	12,83	0	1	3991	150211-Fr_I_Helo-red-alk-tryp-001.raw	8	8	JAW07022.1	Pyrrolidone from Proline	P13:Pyrrolidone from Proline:89.05
**EPAAAAPEAAPE**	37,87	1122,519	12	0,1	562,267	29,89	1,26E+07	1	10038	150211-Fr_I_Helo-red-alk-tryp-001.raw	22	22	P0C8W6.1		
**C(+57.02)VAS(+58.03)DGFIMGDSIGENKEK**	37,87	2113,953	19	-1,8	529,4947	30,61	0	1	10278	150211-Fr_I_Helo-red-alk-tryp-001.raw	2	2	AMO02558.1:JAW07025.1:JAW06984.1	Carbamidomethylation; 2 3-dihydro-2 2-dimethyl-7-benzofuranol N-methyl carbamate	C1:Carbamidomethylation:1000.00;S4:2 3-dihydro-2 2-dimethyl-7-benzofuranol N-methyl carbamate:10.14
**N(sub R)AEIDFSGIPEDIIK**	37,8	1659,835	15	-0,3	830,9247	46,25	6,92E+08	1	15900	150211-Fr_I_Helo-red-alk-tryp-001.raw	2	2	JAW06987.1:JAW07038.1:JAW06992.1		
**ADVDFTGIADSII**	37,73	1335,656	13	0,3	668,8353	45,74	0	1	15720	150211-Fr_I_Helo-red-alk-tryp-001.raw	5	5	JAW07002.1:P86824.1		
**ADVDFTGIADSIIKE(sub K)IK**	37,72	1833,972	17	0,5	612,3317	37,92	0	1	12906	150211-Fr_I_Helo-red-alk-tryp-001.raw	1	1	JAW07002.1		
**GREGYPADSK**	37,71	1078,504	10	0,7	540,2598	5,37	3,88E+07	1	1319	150211-Fr_I_Helo-red-alk-tryp-001.raw	66	66	AAC25688.1:O77463.1:AAC25689.1		
**APEPAPEAAPEAAPEPAAAAPE(+17.03)**	37,69	2069,991	22	-0,2	1036,002	16,74	2,38E+09	1	5422	150211-Fr_I_Helo-red-alk-tryp-001.raw	8	8	JAW07022.1:P86821.1	Replacement of proton with ammonium ion	E22:Replacement of proton with ammonium ion:41.52
**Q(sub E)DGTT(sub V)DINTAGIANSAGVC(+57.02)KPC(+57.02)FK**	37,58	2524,169	24	1	842,3976	35,45	0	1	11997	150211-Fr_I_Helo-red-alk-tryp-001.raw	3	3	AIJ02109.2:P86392.3:V9Z9A3.1:AHE40589.1:AHE40588.1	Carbamidomethylation	C19:Carbamidomethylation:1000.00;C22:Carbamidomethylation:1000.00
**RLTSQS(+58.03)EYAC(+57.02)PAIDKFC(+57.02)EDHC(+57.02)AAK**	37,56	2914,292	24	-1,4	729,5793	15,09	0	1	4809	150211-Fr_I_Helo-red-alk-tryp-001.raw	2	2	P86822.2:P0C8W4.2:AFP87551.1	2 3-dihydro-2 2-dimethyl-7-benzofuranol N-methyl carbamate; Carbamidomethylation	S6:2 3-dihydro-2 2-dimethyl-7-benzofuranol N-methyl carbamate:0.00;C10:Carbamidomethylation:1000.00;C17:Carbamidomethylation:1000.00;C21:Carbamidomethylation:1000.00
**EAAPEAAPEPAAAAPE(+17.03)**	37,52	1507,715	16	-1	754,8642	31,34	0	1	10528	150211-Fr_I_Helo-red-alk-tryp-001.raw	1	1	JAW07022.1:P86821.1	Replacement of proton with ammonium ion	E16:Replacement of proton with ammonium ion:55.73
**K(+42.01)(+43.99)VNEPSFIGDSAILGHQQVLDPVDLVK**	37,48	3003,555	27	-3	751,8938	43,05	0	1	14762	150211-Fr_I_Helo-red-alk-tryp-001.raw	1	1	JAG85213.1	Acetylation (N-term); Carboxylation (DKW)	K1:Acetylation (N-term):1000.00;K1:Carboxylation (DKW):0.00
**FSC(+57.02)FIRPSGFC(+57.02)DGYC(+57.02)K(-.98)(+42.01)**	37,15	2040,864	16	-0,3	681,2952	32,27	0	1	10852	150211-Fr_I_Helo-red-alk-tryp-001.raw	4	4	C0HJM9.1:JAG85185.1:CEF39517.1:P60276.1	Carbamidomethylation; Amidation; Acetylation (K)	C3:Carbamidomethylation:1000.00;C11:Carbamidomethylation:1000.00;C15:Carbamidomethylation:1000.00;K16:Amidation:1000.00;K16:Acetylation (K):1000.00

**ITC(+57.02)FLTAAGYC(+57.02)NTE(+53.92)C(+57.02)TLK**	37,13	2175,873	18	-1,9	726,2968	35,19	0	1	11905	150211-Fr_I_Helo-red-alk-tryp-001.raw	3	3	AAC25688.1:O77463.1:AAC25689.1	Carbamidomethylation; Replacement of 2 protons by iron	C3:Carbamidomethylation:1000.00;C11:Carbamidomethylation:1000.00;E14:Replacement of 2 protons by iron:20.17;C15:Carbamidomethylation:1000.00
**YYC(+57.02)NHQTGLAK**	37,03	1353,614	11	2,4	452,2129	5,77	0	1	1448	150211-Fr_I_Helo-red-alk-tryp-001.raw	1	1	AIJ02109.2:P86392.3:V9Z9A3.1:AHE40589.1:AHE40588.1:JAG85220.1:AHE40596.1:V9ZAY0.1	Carbamidomethylation	C3:Carbamidomethylation:1000.00
**KEALAPE(+21.98)PAPEP**	37,03	1269,622	12	0,4	635,8183	30,72	0	1	10316	150211-Fr_I_Helo-red-alk-tryp-001.raw	12	12	JAW07022.1:P86821.1	Sodium adduct	E7:Sodium adduct:11.59
**WLPLS(+75.98)GPVSGILPHEGK**	36,9	1861,958	17	2,3	621,6614	19,49	0	1	6467	150211-Fr_I_Helo-red-alk-tryp-001.raw	1	1	XP_023242894.1	EDT	S5:EDT:21.91
**KTEPSFIKDNLIPGPPAAFDPDVLIS**	36,9	2780,464	26	1,5	927,8299	52,25	0	1	17939	150211-Fr_I_Helo-red-alk-tryp-001.raw	1	1	AIJ02109.2:P86392.3:V9Z9A3.1:AHE40589.1:AHE40588.1		
**ITC(+57.02)FLTAAGYC(+57.02)NTEC(+57.02)TL**	36,89	1993,858	17	0,1	665,6268	39,82	0	1	13594	150211-Fr_I_Helo-red-alk-tryp-001.raw	7	7	AAC25688.1:O77463.1:AAC25689.1	Carbamidomethylation	C3:Carbamidomethylation:1000.00;C11:Carbamidomethylation:1000.00;C15:Carbamidomethylation:1000.00
**TEPSFIKDNLIPGPPAAFDPDVLIS(+58.03)AMSK**	36,84	3127,603	29	-2,3	1043,539	51,08	0	1	17552	150211-Fr_I_Helo-red-alk-tryp-001.raw	2	2	AIJ02109.2:P86392.3:V9Z9A3.1:AHE40589.1:AHE40588.1	2 3-dihydro-2 2-dimethyl-7-benzofuranol N-methyl carbamate	S25:2 3-dihydro-2 2-dimethyl-7-benzofuranol N-methyl carbamate:3.29
**APEPAPEAAPEAAPE(+17.03)PAAAAPE**	36,73	2069,991	22	-0,5	1036,002	24,23	9,80E+08	1	8193	150211-Fr_I_Helo-red-alk-tryp-001.raw	15	15	JAW07022.1:P86821.1	Replacement of proton with ammonium ion	E15:Replacement of proton with ammonium ion:0.00
**AEIDFSGIPEDIIK(+114.04)QI**	36,64	1900,978	16	0,4	951,4967	50,18	1,46E+08	1	17252	150211-Fr_I_Helo-red-alk-tryp-001.raw	4	4	JAW06987.1:JAW07038.1:JAW06992.1	Ubiquitin	K14:Ubiquitin:58.06
**APEAAPE(+37.96)PAAAAPE**	36,55	1328,565	14	-2,8	665,2879	30,67	0	1	10298	150211-Fr_I_Helo-red-alk-tryp-001.raw	8	8	JAW07022.1:P86821.1:P0C8W6.1	Replacement of proton by potassium	E7:Replacement of proton by potassium:68.46
**AEIDFS(+58.03)GIPEDIIK**	36,53	1603,822	14	-1,3	535,6139	41,45	0	1	14187	150211-Fr_I_Helo-red-alk-tryp-001.raw	19	19	JAW06987.1:JAW07038.1:JAW06992.1:P84190.1	2 3-dihydro-2 2-dimethyl-7-benzofuranol N-methyl carbamate	S6:2 3-dihydro-2 2-dimethyl-7-benzofuranol N-methyl carbamate:1000.00
**W(sub A)EIDFSGIPEDIIK**	36,52	1660,835	14	-0,7	831,4241	50,59	0	1	17387	150211-Fr_I_Helo-red-alk-tryp-001.raw	5	5	JAW06987.1:JAW07038.1:JAW06992.1:P84190.1		
**ET(sub R)AEIDFSGIPEDIIK**	36,5	1775,883	16	-0,6	888,9482	43,17	0	1	14804	150211-Fr_I_Helo-red-alk-tryp-001.raw	2	2	JAW06987.1:JAW07038.1:JAW06992.1		
**APEAAPEAAPEPAAAAPE(+17.03)**	36,48	1675,805	18	-0,4	838,9095	15,32	8,63E+08	1	4893	150211-Fr_I_Helo-red-alk-tryp-001.raw	47	47	JAW07022.1:P86821.1	Replacement of proton with ammonium ion	E18:Replacement of proton with ammonium ion:46.74
**C(+57.02)VAS(+58.03)DGFIMGDSIGENK**	36,31	1856,816	17	-3	619,944	31,1	0	1	10446	150211-Fr_I_Helo-red-alk-tryp-001.raw	2	2	AMO02558.1:JAW07025.1:JAW06984.1	Carbamidomethylation; 2 3-dihydro-2 2-dimethyl-7-benzofuranol N-methyl carbamate	C1:Carbamidomethylation:1000.00;S4:2 3-dihydro-2 2-dimethyl-7-benzofuranol N-methyl carbamate:13.04
**E(+175.04)IDFSGIPEDIIK**	36,14	1649,798	13	2,4	825,908	48,57	0	1	16702	150211-Fr_I_Helo-red-alk-tryp-001.raw	1	1	JAW06987.1:JAW07038.1:JAW06992.1:P84190.1	Naphthalene-2 3-dicarboxaldehyde	E1:Naphthalene-2 3-dicarboxaldehyde:83.60
**KGSSGYC(+57.02)AWPAC(+57.02)Y(+164.06)C(+57.02)**	36,12	1829,698	14	1,8	915,8577	20,49	0	1	6846	150211-Fr_I_Helo-red-alk-tryp-001.raw	1	1	AAC25688.1:O77463.1:AAC25689.1:AAW	Carbamidomethylation; O-	C7:Carbamidomethylation:1000.00;C12:Carbamidomethylati

													72453.1:Q5G8B8.1:AAB29128.1:P56612.1:AIA81145.1:CAA46982.1:P15226.2:P0DMM8.1:1NPI:1B7D:AAB36321.1:AAB36318.1:P56611.1:JAG85191.1:JAG85210.1	Diisopropylphosphorylation	on:1000.00;Y13:O-Diisopropylphosphorylation:66.25;C14:Carbamidomethylation:1000.00
**GIFSIDWGK**	36,04	1021,523	9	-0,2	511,7688	34,25	0	1	11568	150211-Fr_I_Helo-red-alk-tryp-001.raw	7	7	JAW07031.1:JAW06995.1		
**FSC(+57.02)FPRPAGFC(+57.02)D(+17.03)GYC(+57.02)K**	36,03	1984,838	16	-0,4	993,426	21,76	0	1	7312	150211-Fr_I_Helo-red-alk-tryp-001.raw	38	38		Carbamidomethylation; Replacement of proton with ammonium ion	C3:Carbamidomethylation:1000.00;C11:Carbamidomethylation:1000.00;D12:Replacement of proton with ammonium ion:52.86;C15:Carbamidomethylation:1000.00
**ETNAKPPA(+21.98)**	36	848,4004	8	0,3	425,2076	5,53	1,44E+07	1	1366	150211-Fr_I_Helo-red-alk-tryp-001.raw	12	12	JAW06987.1:JAW07038.1:JAW06992.1:P84190.1:JAW07002.1	Sodium adduct	A8:Sodium adduct:55.18
**LVALIPNDQLR**	35,81	1250,735	11	-1	626,374	17,3	0	1	5634	150211-Fr_I_Helo-red-alk-tryp-001.raw	1	1	P0C2F3.1:P69940.2:Q5G8A6.1:JAG85216.1:AAW72464.1:AAW72465.1		
**DQ(sub A)DIIMLTTDRPLADISSEGK**	35,77	2317,147	21	-0,9	773,3889	34,37	7,89E+08	1	11610	150211-Fr_I_Helo-red-alk-tryp-001.raw	5	5	AIJ02113.2		
**NVLVIAPPDPSVFSDPD**	35,77	1780,888	17	2,2	891,4534	47,39	0	1	16298	150211-Fr_I_Helo-red-alk-tryp-001.raw	1	1	AIJ02118.2		
**V(+27.99)NEPSFIGDS(+79.97)AILGHQQVLDPVDLVK**	35,76	2897,421	26	0,5	966,8148	46,4	0	1	15953	150211-Fr_I_Helo-red-alk-tryp-001.raw	1	1	JAG85213.1	Formylation; Phosphorylation (STY)	V1:Formylation:32.15;S10:Phosphorylation (STY):0.00
**FGC(+57.02)PAYEGYC(+57.02)N(+.98)DHC(+57.02)NDIERK**	35,75	2504,978	20	4,9	627,2549	31,12	0	1	10451	150211-Fr_I_Helo-red-alk-tryp-001.raw	2	2	P0C2F3.1:P69940.2	Carbamidomethylation; Deamidation (NQ)	C3:Carbamidomethylation:1000.00;C10:Carbamidomethylation:1000.00;N11:Deamidation (NQ):38.03;C14:Carbamidomethylation:1000.00
**QNEPSFH(sub I)EDSAIPGHQQVLDPVDLVK**	35,7	2898,415	26	2,2	967,1476	46,01	0	1	15818	150211-Fr_I_Helo-red-alk-tryp-001.raw	1	1	AIJ02110.2		
**APEPAPEPAPEAAPEAAPEPAAAAPERRR(+144.04)**	35,69	3059,495	29	4,2	1020,843	41,08	0	1	14051	150211-Fr_I_Helo-red-alk-tryp-001.raw	1	1	JAW07022.1	Condensation product of 3-deoxyglucosone	R29:Condensation product of 3-deoxyglucosone:0.00
**APEAAPEAAPEPAAAAP(+15.99)ER**	35,66	1830,875	19	2,7	916,4471	14,8	0	1	4704	150211-Fr_I_Helo-red-alk-tryp-001.raw	1	1	JAW07022.1	Oxidation or Hydroxylation	P17:Oxidation or Hydroxylation:5.47
**S(+164.06)TQDEIQC(+57.02)EEFC(+57.02)EK**	35,64	1965,774	14	3,4	983,8975	31,67	0	1	10642	150211-Fr_I_Helo-red-alk-tryp-001.raw	1	1	JAW07006.1	O-Diisopropylphosphorylation; Carbamidomethylation	S1:O-Diisopropylphosphorylation:0.00;C8:Carbamidomethylation:1000.00;C12:Carbamidomethylation:1000.00
**ADVDFTGIADS**	35,55	1109,488	11	-1,5	555,7503	18,3	4,64E+08	1	6013	150211-Fr_I_Helo-red-alk-tryp-001.raw	108	108	JAW07002.1:P86824.1		
**IYIGTVFVQTQTMMDTLK**	35,4	2088,064	18	-1,2	1045,038	50,27	0	1	17281	150211-Fr_I_Helo-red-alk-tryp-001.raw	1	1	AMO02558.1:JAW07025.1:JAW06984.1		

**V(+104.03)NEPSFIGDSAILGHQQVLDPVDLVK**	35,32	2893,486	26	-2,2	724,3772	46,19	0	1	15881	150211-Fr_I_Helo-red-alk-tryp-001.raw	1	1	JAG85213.1	Benzoyl	V1:Benzoyl:39.42
**VC(+57.02)DYPANVK**	35,3	1064,496	9	-1,3	533,2546	5,88	0	1	1485	150211-Fr_I_Helo-red-alk-tryp-001.raw	1	1		Carbamidomethylation	C2:Carbamidomethylation:1000.00
**EALAPEPAPEPAPE(+17.03)P**	35,19	1530,757	15	-0,8	766,3849	34,15	3,88E+08	1	11532	150211-Fr_I_Helo-red-alk-tryp-001.raw	10	10	JAW07022.1:P86821.1	Replacement of proton with ammonium ion	E14:Replacement of proton with ammonium ion:0.00
**LTSQS(+58.03)EYAC(+57.02)PAIDKFC(+57.02)EDHC(+57.02)AAK**	35,17	2758,191	23	-3,4	690,5527	17,13	0	1	5569	150211-Fr_I_Helo-red-alk-tryp-001.raw	4	4	P86822.2:P0C8W4.2:AFP87551.1	2 3-dihydro-2 2-dimethyl-7-benzofuranol N-methyl carbamate; Carbamidomethylation	S5:2 3-dihydro-2 2-dimethyl-7-benzofuranol N-methyl carbamate:5.99;C9:Carbamidomethylation:1000.00;C16:Carbamidomethylation:1000.00;C20:Carbamidomethylation:1000.00
**ADVDFTGIADSIIP(sub K)K**	35,17	1560,804	15	-0,3	781,4088	45,74	0	1	15722	150211-Fr_I_Helo-red-alk-tryp-001.raw	2	2	JAW07002.1		
**AEIDFSGIPED**	35,12	1191,53	11	0,2	596,7722	32,43	5,48E+08	1	10909	150211-Fr_I_Helo-red-alk-tryp-001.raw	90	90	JAW06987.1:JAW07038.1:JAW06992.1:P84190.1		
**SEYAC(+57.02)PAIDK**	35,07	1152,512	10	-0,8	577,2629	5,91	0	1	1494	150211-Fr_I_Helo-red-alk-tryp-001.raw	2	2	P86822.2:P0C8W4.2:AFP87551.1:Q0GY42.1:ABE98265.1:JAG85223.1	Carbamidomethylation	C5:Carbamidomethylation:1000.00
**LAPEPAPE(+37.96)PAPEPAPEA**	34,99	1719,776	17	-1,4	860,894	33,89	0	1	11437	150211-Fr_I_Helo-red-alk-tryp-001.raw	1	1	JAW07022.1:P86821.1	Replacement of proton by potassium	E8:Replacement of proton by potassium:9.95
**SDVTGGFPVNSNNC(+57.02)IYPC(+57.02)Y(-94.04)STQDEIQC(+57.02)EEFC(+57.02)EKL**	34,79	3965,649	34	-2,9	1322,887	44,6	0	1	15319	150211-Fr_I_Helo-red-alk-tryp-001.raw	4	4	JAW07006.1	Carbamidomethylation; Dehydroalanine (Y)	C14:Carbamidomethylation:1000.00;C18:Carbamidomethylation:1000.00;Y19:Dehydroalanine (Y):6.63;C27:Carbamidomethylation:1000.00;C31:Carbamidomethylation:1000.00
**ETRPITSWHTIGIL**	34,79	1622,878	14	-0,7	541,9662	36,12	0	1	12241	150211-Fr_I_Helo-red-alk-tryp-001.raw	1	1	JAW07031.1:JAW06995.1		
**EPAPEPAPEPAPEAAPEAAPEPAAAAP(+31.99)ERRR**	34,76	3173,538	31	1,1	794,3926	40,2	0	1	13732	150211-Fr_I_Helo-red-alk-tryp-001.raw	1	1	JAW07022.1	Dihydroxy	P27:Dihydroxy:0.00
**TQFGC(+57.02)PAYEGYC(+57.02)N(+.98)DHC(+57.02)N(+.98)DIER(-.98)**	34,74	2605,99	21	5,1	1304,009	20,95	0	1	7016	150211-Fr_I_Helo-red-alk-tryp-001.raw	2	2	P0C2F3.1:P69940.2	Carbamidomethylation; Deamidation (NQ); Amidation	C5:Carbamidomethylation:1000.00;C12:Carbamidomethylation:1000.00;N13:Deamidation (NQ):0.00;C16:Carbamidomethylation:1000.00;N17:Deamidation (NQ):0.00;R21:Amidation:1000.00
**TLAN(sub T)DDQCIVIEILSVTDK**	34,68	2089,061	19	0,1	697,3611	48,8	0	1	16780	150211-Fr_I_Helo-red-alk-tryp-001.raw	1	1	AIJ02113.2		
**FFT(+164.06)LPQASC(+57.02)LR**	34,67	1502,736	11	4,5	501,9214	37,91	0	1	12900	150211-Fr_I_Helo-red-alk-tryp-001.raw	2	2	JAW07039.1	O-Diisopropylphosphorylation; Carbamidomethylation	T3:O-Diisopropylphosphorylation:0.00;C9:Carbamidomethylation:1000.00

**PSFIEDSAIPGHQQVLDPVDLVK(+14.02)NMAK(+42.01)**	34,63	3003,538	27	-0,1	1002,186	43,74	0	1	15010	150211-Fr_I_Helo-red-alk-tryp-001.raw	3	3	AIJ02110.2	Methylation(KR); Acetylation (K)	K23:Methylation(KR):0.00;K27:Acetylation (K):0.00
**EALAPEPAPE(+17.03)PAPEP**	34,63	1530,757	15	-1,1	766,3846	32,69	0	1	11004	150211-Fr_I_Helo-red-alk-tryp-001.raw	41	41	JAW07022.1:P86821.1	Replacement of proton with ammonium ion	E10:Replacement of proton with ammonium ion:19.58
**EPAPEAAPEAAPEPAAAAPER(+28.03)**	34,54	2069,006	21	-0,5	1035,51	15,01	0	1	4781	150211-Fr_I_Helo-red-alk-tryp-001.raw	10	10	JAW07022.1	Dimethylation(KR)	R21:Dimethylation(KR):1000.00
**S(+58.03)QIENEAVGLALGGGAC(+57.02)LTC(+57.02)EK**	34,42	2334,107	22	-4,3	779,0396	36,76	0	1	12476	150211-Fr_I_Helo-red-alk-tryp-001.raw	1	1	AIJ02111.3	2 3-dihydro-2 2-dimethyl-7-benzofuranol N-methyl carbamate; Carbamidomethylation	S1:2 3-dihydro-2 2-dimethyl-7-benzofuranol N-methyl carbamate:1000.00;C17:Carbamidomethylation:1000.00;C20:Carbamidomethylation:1000.00
**PAPEPAPEPAPEAAPEAAPEPAAAAPERR(+160.04)R**	34,41	3172,542	30	-2,5	1587,274	41,23	0	1	14108	150211-Fr_I_Helo-red-alk-tryp-001.raw	2	2	JAW07022.1	Condensation product of glucosone	R29:Condensation product of glucosone:0.00
**TDQALTEYVTQMYT(+42.01)GVQNMYDTMNLEIK**	34,3	3341,514	28	4,7	1114,851	53,62	0	1	18386	150211-Fr_I_Helo-red-alk-tryp-001.raw	1	1	JAW07039.1	Acetylation (TSCYH)	T14:Acetylation (TSCYH):0.00
**EPAPEPAPEAAPEAAPEPAAAAPER(+14.02)R(-.98)**	34,18	2604,293	26	-3,7	1303,149	37,28	0	1	12669	150211-Fr_I_Helo-red-alk-tryp-001.raw	1	1	JAW07022.1	Methylation(KR); Amidation	R25:Methylation(KR):1000.00;R26:Amidation:1000.00
**EPAPEPAPEPAPEAAPEAAPEPAAAAPER(+31.99)RR**	33,98	3173,538	31	-0,5	1587,775	40,63	0	1	13887	150211-Fr_I_Helo-red-alk-tryp-001.raw	2	2	JAW07022.1	Dihydroxy	R29:Dihydroxy:0.00
**DAPPLN(sub D)LPQ(sub D)GVGFK**	33,95	1451,777	14	-0,6	726,8954	36,18	0	1	12261	150211-Fr_I_Helo-red-alk-tryp-001.raw	5	5	XP_023239442.1		
**AEIDFSGIPEDII**	33,95	1417,698	13	-0,3	709,8559	47,03	7,20E+08	1	16173	150211-Fr_I_Helo-red-alk-tryp-001.raw	9	9	JAW06987.1:JAW07038.1:JAW06992.1:P84190.1		
**AEIDFSGIPEDIIK(+43.01)QIK(+14.02)**	33,89	1972,052	17	-0,8	658,3573	47,73	0	1	16415	150211-Fr_I_Helo-red-alk-tryp-001.raw	1	1	JAW06987.1:JAW07038.1:JAW06992.1	Carbamylation; Methylation(KR)	K14:Carbamylation:0.00;K17:Methylation(KR):0.00
**C(+57.02)K(+172.05)ITC(+57.02)FLTAAGYC(+57.02)NTEC(+57.02)TLK**	33,8	2582,131	20	4,9	861,7219	36,41	0	1	12345	150211-Fr_I_Helo-red-alk-tryp-001.raw	1	1	AAC25688.1:O77463.1:AAC25689.1	Carbamidomethylation; Menadione hydroquinone derivative	C1:Carbamidomethylation:1000.00;K2:Menadione hydroquinone derivative:100.77;C5:Carbamidomethylation:1000.00;C13:Carbamidomethylation:1000.00;C17:Carbamidomethylation:1000.00
**ET(+79.97)NAKPP(+31.99)AR**	33,78	1094,476	9	-1	548,2446	5,69	0	1	1418	150211-Fr_I_Helo-red-alk-tryp-001.raw	3	3	JAW06987.1:JAW07038.1:JAW06992.1:JAW07002.1	Phosphorylation (STY); Dihydroxy	T2:Phosphorylation (STY):1000.00;P7:Dihydroxy:0.00
**EPAPEAAPEAAPEPAAAAPERRR(+453.21)**	33,75	2806,39	23	-1,9	936,4688	42,4	0	1	14531	150211-Fr_I_Helo-red-alk-tryp-001.raw	2	2	JAW07022.1	Puromycin	R23:Puromycin:1000.00
**EPAAAAPE(+43.99)AAPE**	33,7	1166,509	12	-2,2	584,2605	21,44	0	1	7197	150211-Fr_I_Helo-red-alk-tryp-001.raw	55	55	P0C8W6.1	Carboxylation (E)	E8:Carboxylation (E):16.51
**E(-18.01)IDFSGIPEDIIKQ**	33,59	1584,804	14	2,1	793,4106	46,67	0	1	16048	150211-Fr_I_Helo-red-alk-tryp-001.raw	1	1	JAW06987.1:JAW07038.1:JAW06992.1	Pyro-glu from E	E1:Pyro-glu from E:1000.00
**AEIDFSGIPED(+21.98)**	33,23	1213,512	11	0,5	607,7633	33,21	0	1	11191	150211-Fr_I_Helo-red-alk-tryp-001.raw	3	3	JAW06987.1:JAW07038.1:JAW06992.1:P84190.1	Sodium adduct	D11:Sodium adduct:0.00
**S(+59.02)SGYC(+57.02)AWPAC(+57.02)YC(+57.02)YGLPNWVK**	33,2	2497,048	20	0,1	833,3566	49,06	0	1	16869	150211-Fr_I_Helo-red-alk-tryp-001.raw	1	1	AAW72453.1:Q5G8B8.1:AAB29128.1:P56	Aminoethylcysteine; Carbamidomethylation	S1:Aminoethylcysteine:0.00;C5:Carbamidomethylation:1000

													612.1:AIA81145.1:CAA46982.1:P15226.2:P0DMM8.1:1NPI:1B7D:AAB36321.1:AAB36318.1:P56611.1:JAG85191.1:JAG85210.1		.00;C10:Carbamidomethylation:1000.00;C12:Carbamidomethylation:1000.00
**QIGIFDSK**	33,16	906,4811	8	0,5	454,248	28,88	0	1	9709	150211-Fr_I_Helo-red-alk-tryp-001.raw	13	13	AMO02558.1:JAW07025.1:JAW06984.1		
**APEAAPEPAAAAPER(+212.01)RRR**	33,13	2127,022	18	-2,5	1064,516	17,25	0	1	5615	150211-Fr_I_Helo-red-alk-tryp-001.raw	6	6	JAW07022.1	phosphate-ribosylation	R15:phosphate-ribosylation:0.00
**PEAAPEAAPEPAAAAP(+15.99)ER**	33,04	1759,838	18	3,4	880,9291	14,25	0	1	4502	150211-Fr_I_Helo-red-alk-tryp-001.raw	6	6	JAW07022.1	Oxidation or Hydroxylation	P16:Oxidation or Hydroxylation:0.00
**PEPAPEPAPEPAPEAAPEAAPEPAAAAPE(+129.04)RR**	32,99	3211,542	31	-1	1606,777	43,64	0	1	14974	150211-Fr_I_Helo-red-alk-tryp-001.raw	1	1	JAW07022.1	Monoglutamyl	E29:Monoglutamyl:2.90
**PEAAPEAAPEPAAAAPERR**	32,94	1899,944	19	-4,5	950,9749	17,59	0	1	5745	150211-Fr_I_Helo-red-alk-tryp-001.raw	1	1	JAW07022.1		
**ADVDFTGIAD(+21.98)S**	32,93	1131,47	11	0,1	566,7421	33,34	0	1	11236	150211-Fr_I_Helo-red-alk-tryp-001.raw	6	6	JAW07002.1:P86824.1	Sodium adduct	D10:Sodium adduct:20.00
**AEIDFSGIPEDIIK(+71.04)EIK**	32,89	1987,051	17	-0,4	663,3574	48,76	0	1	16768	150211-Fr_I_Helo-red-alk-tryp-001.raw	1	1	P84190.1	Propionamide (K X@N-term)	K14:Propionamide (K X@N-term):6.63
**ALAPEPAPEPAPEPAPEAAPEAAPEPAAAAPE**	32,86	3025,456	32	0,7	757,3717	40,46	0	1	13827	150211-Fr_I_Helo-red-alk-tryp-001.raw	1	1	JAW07022.1:P86821.1		
**T(+298.19)C(+57.02)FLTAAGYC(+57.02)NTEC(+57.02)TLK**	32,8	2307,063	17	2,8	770,0303	21,26	0	1	7130	150211-Fr_I_Helo-red-alk-tryp-001.raw	1	1	AAC25688.1:O77463.1:AAC25689.1	Levuglandinyl-lysine anhyropyrrole adduct; Carbamidomethylation	T1:Levuglandinyl-lysine anhyropyrrole adduct:92.02;C2:Carbamidomethylation:1000.00;C10:Carbamidomethylation:1000.00;C14:Carbamidomethylation:1000.00
**IDFSGIPEDIIKEI**	32,38	1587,84	14	-0,2	794,9268	50,16	0	1	17245	150211-Fr_I_Helo-red-alk-tryp-001.raw	2	2	P84190.1		
**KETNAKPPA**	32,32	954,5134	9	0	478,264	5,2	2,06E+08	1	1266	150211-Fr_I_Helo-red-alk-tryp-001.raw	125	125	JAW06987.1:JAW07038.1:JAW06992.1:P84190.1:JAW07002.1		
**AEIDFSGIPEDI**	32,2	1304,614	12	0	653,314	42,54	0	1	14579	150211-Fr_I_Helo-red-alk-tryp-001.raw	13	13	JAW06987.1:JAW07038.1:JAW06992.1:P84190.1		
**M(sub A)M(ins)EIDFSGIPEDIIK**	32,18	1736,836	15	-0,8	869,4248	47,67	0	1	16395	150211-Fr_I_Helo-red-alk-tryp-001.raw	1	1	P84190.1		
**Q(sub A)SSGYC(+57.02)AWPAC(+57.02)YC(+57.02)YGVPDHIK**	32,11	2518,05	21	-0,9	840,3566	36,88	4,81E+09	1	12520	150211-Fr_I_Helo-red-alk-tryp-001.raw	1	1	JAW07016.1:JAW06979.1:P68411.1:1913435A:P68410.1:AAB36322.1	Carbamidomethylation	C6:Carbamidomethylation:1000.00;C11:Carbamidomethylation:1000.00;C13:Carbamidomethylation:1000.00
**ITC(+57.02)FLT(+59.02)AAGYC(+57.02)NTEC(+57.02)TLK**	32,08	2180,973	18	1,9	1091,496	35,31	0	1	11946	150211-Fr_I_Helo-red-alk-tryp-001.raw	1	1	AAC25688.1:O77463.1:AAC25689.1	Carbamidomethylation; Aminoethylcysteine	C3:Carbamidomethylation:1000.00;T6:Aminoethylcysteine:32.48;C11:Carbamidomethylation:1000.00;C15:Carbamidomethylation:1000.00

**PAPEAAPEAAPEPAAAAPERRR(+380.15)**	32,06	2604,282	22	-0,8	1303,147	37	0	1	12566	150211-Fr_I_Helo-red-alk-tryp-001.raw	3	3	JAW07022.1	Nucleophilic addition to cytopiloyne+H2O	R22:Nucleophilic addition to cytopiloyne+H2O:14.04
**QNQ(sub E)PY(sub S)FIEDSAIPGHQQVLDPVDLVK**	32,06	2949,487	26	3	738,3813	46,46	0	1	15976	150211-Fr_I_Helo-red-alk-tryp-001.raw	1	1	AIJ02110.2		
**APEPAPEAAPEPVAAAAPER**	32,05	1939,964	20	-0,8	970,9885	33,13	0	1	11162	150211-Fr_I_Helo-red-alk-tryp-001.raw	1	1	JAG85215.1		
**QIKET(+114.04)NAKPPA**	32,05	1309,699	11	-1,5	655,8558	5,78	0	1	1449	150211-Fr_I_Helo-red-alk-tryp-001.raw	1	1	JAW06987.1:JAW07038.1:JAW06992.1	Ubiquitin	T5:Ubiquitin:9.40
**KLGELQDDGTVAYNTAGIS(+58.03)LGSGVC(+57.02)K**	32,03	2710,336	26	-1,3	1356,173	36,04	0	1	12212	150211-Fr_I_Helo-red-alk-tryp-001.raw	1	1	AIJ02110.2:JAG85213.1	2 3-dihydro-2 2-dimethyl-7-benzofuranol N-methyl carbamate; Carbamidomethylation	S19:2 3-dihydro-2 2-dimethyl-7-benzofuranol N-methyl carbamate:47.09;C25:Carbamidomethylation:1000.00
**LVGILFYDILR**	32,03	1320,781	11	0,3	661,3977	50,25	0	1	17274	150211-Fr_I_Helo-red-alk-tryp-001.raw	1	1	JAW07019.1:JAW06978.1		
**S(+58.03)PC(+57.02)IIIDYLC(+57.02)VTETTFTER**	32	2375,126	19	-3,3	792,7134	51,7	0	1	17757	150211-Fr_I_Helo-red-alk-tryp-001.raw	1	1	AIJ02110.2	2 3-dihydro-2 2-dimethyl-7-benzofuranol N-methyl carbamate; Carbamidomethylation	S1:2 3-dihydro-2 2-dimethyl-7-benzofuranol N-methyl carbamate:1000.00;C3:Carbamidomethylation:1000.00;C10:Carbamidomethylation:1000.00
**AEIDFSGIPEDIIK(+162.08)EIK**	31,82	2078,095	17	-4,1	1040,051	46,75	0	1	16077	150211-Fr_I_Helo-red-alk-tryp-001.raw	1	1	P84190.1	O-Pinacolylmethylphosphonylation	K14:O-Pinacolylmethylphosphonylation:9.42
**APEPAPEPAPE(+21.98)PAPEA**	31,82	1590,718	16	-0,1	796,366	32,29	0	1	10862	150211-Fr_I_Helo-red-alk-tryp-001.raw	1	1	JAW07022.1:P86821.1:P0C8W6.1	Sodium adduct	E11:Sodium adduct:33.58
**DW(sub A)DIIMLTTDRPLADISSEGK**	31,78	2375,168	21	1	792,7307	35,72	0	1	12094	150211-Fr_I_Helo-red-alk-tryp-001.raw	1	1	AIJ02113.2		
**D(-15.01)SAIPGHQQVLDPVDLVK**	31,7	1915,005	18	-1,7	958,5082	49,35	0	1	16971	150211-Fr_I_Helo-red-alk-tryp-001.raw	1	1	AIJ02110.2	ISD (z+2)-series	D1:ISD (z+2)-series:1000.00
**TFDC(+57.02)SNGPWDLSTV**	31,67	1597,672	14	3,6	799,8461	39,96	0	1	13644	150211-Fr_I_Helo-red-alk-tryp-001.raw	3	3	JAW07006.1	Carbamidomethylation	C4:Carbamidomethylation:1000.00
**EPAPEPAPEAAPEAAPEPAAAAPERR(-.98)(+14.02)**	31,48	2604,293	26	-4,9	1303,147	37,51	0	1	12752	150211-Fr_I_Helo-red-alk-tryp-001.raw	1	1	JAW07022.1	Amidation; Methylation(KR)	R26:Amidation:1000.00;R26:Methylation(KR):0.00
**EIKETNAKPPA**	31,48	1196,64	11	-2,5	599,3258	9,98	0	1	2974	150211-Fr_I_Helo-red-alk-tryp-001.raw	2	2	P84190.1		
**AEIDFS(+114.04)GIPEDIIK**	31,41	1659,835	14	-0,6	830,9245	47	0	1	16164	150211-Fr_I_Helo-red-alk-tryp-001.raw	6	6	JAW06987.1:JAW07038.1:JAW06992.1:P84190.1	Ubiquitin	S6:Ubiquitin:32.01
**LTSKSE(+129.04)YAC(+57.02)PVIDKFC(+57.02)EDHC(+57.02)AAK**	31,39	2857,272	23	0	572,4617	14,94	0	1	4756	150211-Fr_I_Helo-red-alk-tryp-001.raw	3	3	Q0GY43.1:ABE98264.1	Monoglutamyl; Carbamidomethylation	E6:Monoglutamyl:56.41;C9:Carbamidomethylation:1000.00;C16:Carbamidomethylation:1000.00;C20:Carbamidomethylation:1000.00
**IW(+31.99)TSETNK**	31,37	1009,472	8	0,3	505,7432	5,8	0	1	1456	150211-Fr_I_Helo-red-alk-tryp-001.raw	30	30	AAC25688.1:O77463.1:AAC25689.1	Dihydroxy	W2:Dihydroxy:53.33
**APEPAPEAAPEAAPEPA**	31,37	1613,757	17	-0,2	807,8857	32,22	0	1	10834	150211-Fr_I_Helo-red-alk-tryp-001.raw	3	3	JAW07022.1:P86821.1		

**TQFGC(+57.02)PAYEGY(+44.99)C(+57.02)NNHC(+57.02)QDIER**	31,27	2663,023	21	0,9	888,6822	24,17	0	1	8173	150211-Fr_I_Helo-red-alk-tryp-001.raw	9	9	Q5G8A6.1:JAG85216.1:AAW72464.1:AAW72465.1	Carbamidomethylation; Oxidation to nitro	C5:Carbamidomethylation:1000.00;Y11:Oxidation to nitro:19.05;C12:Carbamidomethylation:1000.00;C16:Carbamidomethylation:1000.00
**YC(+57.02)YGLPDSVK**	31,23	1200,549	10	-1	601,2809	31,65	0	1	10633	150211-Fr_I_Helo-red-alk-tryp-001.raw	1	1	AAC25688.1:O77463.1:AAC25689.1	Carbamidomethylation	C2:Carbamidomethylation:1000.00
**EALAPEPAP**	31,18	893,4494	9	0,2	447,7321	30,51	0	1	10243	150211-Fr_I_Helo-red-alk-tryp-001.raw	1	1	JAW07022.1:P86821.1:P0C8W6.1		
**AEIDFS(+58.03)GIPEDIIKQIK**	31,05	1973,059	17	-2,7	987,5344	46,85	0	1	16111	150211-Fr_I_Helo-red-alk-tryp-001.raw	6	6	JAW06987.1:JAW07038.1:JAW06992.1	2 3-dihydro-2 2-dimethyl-7-benzofuranol N-methyl carbamate	S6:2 3-dihydro-2 2-dimethyl-7-benzofuranol N-methyl carbamate:1000.00
**KEALAPEPAP**	31,02	1021,544	10	0	511,7794	20,36	0	1	6795	150211-Fr_I_Helo-red-alk-tryp-001.raw	78	78	JAW07022.1:P86821.1:P0C8W6.1		
**FKTNEALTEYVTQ(+.98)MFTGVQNMYDTM(+15.99)NLDIKI**	30,73	3674,72	31	-0,1	735,9511	49,59	0	1	17053	150211-Fr_I_Helo-red-alk-tryp-001.raw	1	1	JAG85190.1	Deamidation (NQ); Oxidation (M)	Q13:Deamidation (NQ):0.00;M25:Oxidation (M):3.11
**Q(sub A)EIDFSGIPEDIIKEI**	30,72	1844,941	16	-0,6	923,4771	51,2	0	1	17592	150211-Fr_I_Helo-red-alk-tryp-001.raw	3	3	P84190.1		
**AEIDFSGIPEDIIKE(-.98)**	30,71	1673,851	15	1,2	837,9338	67,33	4,64E+06	1	22808	150211-Fr_I_Helo-red-alk-tryp-001.raw	36	36	P84190.1	Amidation	E15:Amidation:1000.00
**ALAPEPAPEP**	30,69	990,5021	10	0,1	496,2584	31,36	0	1	10534	150211-Fr_I_Helo-red-alk-tryp-001.raw	3	3	JAW07022.1:P86821.1		
**E(+27.99)ALAPEPAPEPAPEPA**	30,68	1612,762	16	-1,2	807,3873	21,44	0	1	7198	150211-Fr_I_Helo-red-alk-tryp-001.raw	24	24	JAW07022.1	Formylation	E1:Formylation:1000.00
**APEPAPEPAPEAAPEAAPEPAAAAPE(+258.09)RRR**	30,68	3173,538	29	0	1587,776	42,73	0	1	14649	150211-Fr_I_Helo-red-alk-tryp-001.raw	1	1	JAW07022.1	Diglutamyl	E26:Diglutamyl:0.00
**V(+42.01)NEPSFIGD(+43.99)SAILGHQQVLDPVDLVK**	30,56	2875,46	26	-5	959,4893	52,05	0	1	17872	150211-Fr_I_Helo-red-alk-tryp-001.raw	1	1	JAG85213.1	Acetylation (N-term); Carboxylation (DKW)	V1:Acetylation (N-term):1000.00;D9:Carboxylation (DKW):67.73
**SDPPFITK**	30,55	903,4702	8	1,1	452,7429	29,28	0	1	9839	150211-Fr_I_Helo-red-alk-tryp-001.raw	1	1	AMO02558.1:JAW07025.1:JAW06984.1		
**S(+71.04)VTPDGPQIR**	30,5	1139,594	10	0,1	570,8041	30,1	0	1	10107	150211-Fr_I_Helo-red-alk-tryp-001.raw	2	2	JAG85161.1	Propionamide (K X@N-term)	S1:Propionamide (K X@N-term):1000.00
**NLLDTLNLGIK**	30,47	1212,708	11	2,6	607,3627	36,72	0	1	12461	150211-Fr_I_Helo-red-alk-tryp-001.raw	1	1	AIJ02110.2:JAG85213.1		
**TEPSFIKDNLIPGPPAAFDPDVLIS(+59.02)AMSK**	30,45	3128,593	29	1,6	1043,873	50,7	0	1	17423	150211-Fr_I_Helo-red-alk-tryp-001.raw	2	2	AIJ02109.2:P86392.3:V9Z9A3.1:AHE40589.1:AHE40588.1	Aminoethylcysteine	S25:Aminoethylcysteine:0.00
**K(+14.96)GSSGYC(+57.02)AWPAC(+57.02)YC(+57.02)YGLPDSVK**	30,43	2540,045	22	5	847,6931	37,36	0	1	12698	150211-Fr_I_Helo-red-alk-tryp-001.raw	1	1	AAC25688.1:O77463.1:AAC25689.1	Alpha-amino adipic acid; Carbamidomethylation	K1:Alpha-amino adipic acid:54.14;C7:Carbamidomethylation:1000.00;C12:Carbamidomethylation:1000.00;C14:Carbamidomethylation:1000.00
**TITIAHEAGHMLGLP(+31.99)HDGQ(+.98)ESTEVGVPNGPGAK**	30,43	3352,599	33	-0,2	671,527	17,9	0	1	5862	150211-Fr_I_Helo-red-alk-tryp-001.raw	1	1	JAW07039.1	Dihydroxy; Deamidation (NQ)	P15:Dihydroxy:16.22;Q19:Deamidation (NQ):18.04
**F(+164.06)FTLPQASC(+57.02)LR**	30,29	1502,736	11	3,2	752,3775	38,91	0	1	13265	150211-Fr_I_Helo-red-alk-tryp-001.raw	2	2	JAW07039.1	O-Diisopropylphosphorylati	F1:O-Diisopropylphosphorylation:32

														on; Carbamidomethylation	.28;C9:Carbamidomethylation: 1000.00
**S(+58.01)SGYC(+57.02)AWPAC (+57.02)YC(+57.02)YGLPDSVK**	30,29	2397,971	20	2,1	800,3325	44,01	0	1	15109	150211-Fr_I_Helo-red-alk-tryp-001.raw	1	1	AAC25688.1:O77463 .1:AAC25689.1	Carboxymethyl (KW X@N-term); Carbamidomethylation	S1:Carboxymethyl (KW X@N-term):0.00;C5:Carbamidomethylation:1000.00;C10:Carbamidomethylation:1000.00;C12:Carbamidomethylation:1000.00
**KVNEPSFIGDS(+86.00)AILGHQ QVLDPVDLVK**	30,2	3003,555	27	-4,7	1002,188	41,94	0	1	14363	150211-Fr_I_Helo-red-alk-tryp-001.raw	1	1	JAG85213.1	Malonylation	S11:Malonylation:0.00
**Q(sub A)EIDFSGIPEDIIKR(sub E)**	30,03	1758,915	15	0,2	587,3124	37,01	0	1	12569	150211-Fr_I_Helo-red-alk-tryp-001.raw	2	2	P84190.1		
**Q(+.98)IKETNAKPPA**	30,02	1196,64	11	-2,6	599,3257	15,07	0	1	4803	150211-Fr_I_Helo-red-alk-tryp-001.raw	6	6	JAW06987.1:JAW07 038.1:JAW06992.1	Deamidation (NQ)	Q1:Deamidation (NQ):14.62
**T(+87.03)SKSEYAC(+57.02)PVI DKFC(+57.02)EDHC(+57.02)AA K**	29,78	2702,178	22	-3,7	676,5491	31,97	0	1	10744	150211-Fr_I_Helo-red-alk-tryp-001.raw	8	8	Q0GY43.1:ABE98264 .1	Glycidamide adduct; Carbamidomethylation	T1:Glycidamide adduct:0.00;C8:Carbamidomethylation:1000.00;C15:Carbamidomethylation:1000.00;C19:Carbamidomethylation:1000.00
**PAPEPAPEAAPEAAPEPAAAAPE R(+199.07)R**	29,71	2661,286	25	4,4	1331,656	37,28	0	1	12670	150211-Fr_I_Helo-red-alk-tryp-001.raw	1	1	JAW07022.1	Oxidized Arginine biotinylated with biotin hydrazide	R24:Oxidized Arginine biotinylated with biotin hydrazide:0.00
**AEIDFSGIPEDIIK(+114.04)EIK**	29,65	2030,057	17	4,4	1016,04	52,31	0	1	17958	150211-Fr_I_Helo-red-alk-tryp-001.raw	1	1	P84190.1	Ubiquitin	K14:Ubiquitin:5.77
**E(-18.01)IKETN(+.98)AKPPA**	29,61	1179,614	11	-3,3	590,8121	5,88	0	1	1487	150211-Fr_I_Helo-red-alk-tryp-001.raw	1	1	P84190.1	Pyro-glu from E; Deamidation (NQ)	E1:Pyro-glu from E:1000.00;N6:Deamidation (NQ):1000.00
**Q(sub E)IDFSGIPEDIIK**	29,57	1473,772	13	-0,6	737,8926	46,74	0	1	16072	150211-Fr_I_Helo-red-alk-tryp-001.raw	2	2	JAW06987.1:JAW07038.1:JAW06992.1:P84190.1		
**GKEGYAMDHEGC(+57.02)K**	29,53	1480,607	13	-0,2	494,543	5,38	3,49E+06	1	1320	150211-Fr_I_Helo-red-alk-tryp-001.raw	3	3	JAW07016.1:JAW06 979.1:C0HJM9.1:JAG 85185.1:CEF39517.1	Carbamidomethylation	C12:Carbamidomethylation:1000.00
**AQ(sub E)V(sub I)DFSGIPEDIIK**	29,52	1530,793	14	-0,7	766,4032	46,1	0	1	15850	150211-Fr_I_Helo-red-alk-tryp-001.raw	3	3	JAW06987.1:JAW07038.1:JAW06992.1:P84190.1		
**PEAAPEAAPEPAAAAPERR(+14. 02)R**	29,47	2070,061	20	-0,2	691,0273	12,45	0	1	3851	150211-Fr_I_Helo-red-alk-tryp-001.raw	1	1	JAW07022.1	Methylation(KR)	R19:Methylation(KR):0.00
**EALAPEPAPEPAPEPAPE(+37.03)AAPEAAPEPA**	29,46	2752,323	28	1,7	918,4498	38,42	0	1	13089	150211-Fr_I_Helo-red-alk-tryp-001.raw	1	1	JAW07022.1:P86821 .1	Propargylamine	E18:Propargylamine:0.00
**GGIFDIADEILPITTN(+.98)FFR(- .98)**	29,41	2138,105	19	4,6	1070,065	61,44	8,61E+05	1	20918	150211-Fr_I_Helo-red-alk-tryp-001.raw	1	1	JAW07023.1	Deamidation (NQ); Amidation	N16:Deamidation (NQ):1000.00;R19:Amidation: 1000.00
**PEAAPEAAPEPAAAAPER(+380. 15)RR**	29,32	2436,192	20	-2,4	1219,1	19,55	0	1	6491	150211-Fr_I_Helo-red-alk-tryp-001.raw	1	1	JAW07022.1	Nucleophilic addition to cytopiloyne+H2O	R18:Nucleophilic addition to cytopiloyne+H2O:0.00
**ITC(+57.02)FLTAAGYC(+57.02) NTE(+17.03)**	29,32	1636,723	14	-1,9	819,367	20,24	0	1	6751	150211-Fr_I_Helo-red-alk-tryp-001.raw	13	13	AAC25688.1:O77463 .1:AAC25689.1	Carbamidomethylation; Replacement of proton with ammonium ion	C3:Carbamidomethylation:1000.00;C11:Carbamidomethylation:1000.00;E14:Replacement of proton with ammonium ion:1000.00

**APEAAPEPAAAAPE(+17.03)AAPE**	29,29	1675,805	18	-0,4	838,9095	25,72	0	1	8692	150211-Fr_I_Helo-red-alk-tryp-001.raw	3	3	P0C8W6.1	Replacement of proton with ammonium ion	E14:Replacement of proton with ammonium ion:7.89
**KMFPDIEK**	29,29	1006,516	8	-0,1	504,2651	12,23	0	1	3775	150211-Fr_I_Helo-red-alk-tryp-001.raw	30	30			
**AEV(sub I)DFSGIPEDIIK**	29,22	1531,777	14	-2,2	766,894	41,26	0	1	14116	150211-Fr_I_Helo-red-alk-tryp-001.raw	3	3	JAW06987.1:JAW07038.1:JAW06992.1:P84190.1		
**ETRPITSWHT(+59.02)IGILMRWDKELQISYVFK**	29,17	3505,837	28	2,3	585,3148	48,15	0	1	16559	150211-Fr_I_Helo-red-alk-tryp-001.raw	1	1	JAW07031.1:JAW06995.1	Aminoethylcysteine	T10:Aminoethylcysteine:6.65
**RLTSESEYAC(+57.02)PAIDKFC(+57.02)EDHC(+57.02)AAK**	29,15	2857,247	24	3	953,4257	32,23	0	1	10840	150211-Fr_I_Helo-red-alk-tryp-001.raw	1	1	Q0GY42.1:ABE98265.1:JAG85223.1	Carbamidomethylation	C10:Carbamidomethylation:1000.00;C17:Carbamidomethylation:1000.00;C21:Carbamidomethylation:1000.00
**G(sub E)IDFSGIPEDIIK**	29,07	1402,734	13	-0,7	702,374	39,56	1,49E+09	1	13500	150211-Fr_I_Helo-red-alk-tryp-001.raw	7	7	JAW06987.1:JAW07038.1:JAW06992.1:P84190.1		
**AEIDFSGIPEDIIK(+109.05)QI**	29,07	1895,988	16	-0,3	949,001	50,57	0	1	17383	150211-Fr_I_Helo-red-alk-tryp-001.raw	2	2	JAW06987.1:JAW07038.1:JAW06992.1	Methylpyrroline	K14:Methylpyrroline:1000.00
**G(sub N)DYLLYC(+57.02)DFSDKDPK**	28,94	1834,808	15	-1,8	918,4098	20,08	0	1	6690	150211-Fr_I_Helo-red-alk-tryp-001.raw	2	2	JAG85161.1	Carbamidomethylation	C7:Carbamidomethylation:1000.00
**E(sub A)SSGYCAWPAC(+57.02)YC(+57.02)YGVPDHIK**	28,9	2462,013	21	4,4	821,6819	36,49	0	1	12373	150211-Fr_I_Helo-red-alk-tryp-001.raw	2	2	JAW07016.1:JAW06979.1:P68411.1:1913435A:P68410.1:AAB36322.1	Carbamidomethylation	C11:Carbamidomethylation:1000.00;C13:Carbamidomethylation:1000.00
**EPAPEAAPEAAPEPAAAAPERRR(+380.15)**	28,87	2733,325	23	1,9	912,1172	37,78	0	1	12854	150211-Fr_I_Helo-red-alk-tryp-001.raw	3	3	JAW07022.1	Nucleophilic addition to cytopiloyne+H2O	R23:Nucleophilic addition to cytopiloyne+H2O:5.03
**FTGIADSIIK**	28,86	1063,591	10	0,8	532,8033	15,57	0	1	4985	150211-Fr_I_Helo-red-alk-tryp-001.raw	1	1	JAW07002.1:P86824.1		
**P(sub A)EV(sub I)DFSGIPEDIIK**	28,86	1557,793	14	0,3	779,9038	44,61	0	1	15324	150211-Fr_I_Helo-red-alk-tryp-001.raw	10	10	JAW06987.1:JAW07038.1:JAW06992.1:P84190.1		
**FINGGTDNSGIIITLLPGS(sub T)DS(sub P)SVTK**	28,85	2505,296	25	1,2	1253,657	47,63	1,84E+08	1	16381	150211-Fr_I_Helo-red-alk-tryp-001.raw	1	1	XP_023239442.1		
**IDFSGIPE(+53.92)DIIK**	28,83	1399,632	12	0,6	467,5516	37,81	0	1	12865	150211-Fr_I_Helo-red-alk-tryp-001.raw	1	1	JAW06987.1:JAW07038.1:JAW06992.1:P84190.1	Replacement of 2 protons by iron	E8:Replacement of 2 protons by iron:0.00
**TNEALTEYVTQMFTGVQN(+.98)MYDTM(+15.99)NLDIKI**	28,81	3399,556	29	-0,6	850,8958	49,35	0	1	16969	150211-Fr_I_Helo-red-alk-tryp-001.raw	2	2	JAG85190.1	Deamidation (NQ); Oxidation (M)	N18:Deamidation (NQ):0.00;M23:Oxidation (M):11.12
**AQVIGITPF**	28,75	944,5331	9	0,4	473,274	33,01	0	1	11118	150211-Fr_I_Helo-red-alk-tryp-001.raw	12	12	AIJ02110.2:JAG85213.1		
**P(+41.03)SGFC(+57.02)DGYC(+57.02)K**	28,69	1230,48	10	-1,4	616,2463	6,02	0	1	1533	150211-Fr_I_Helo-red-alk-tryp-001.raw	38	38	C0HJM9.1:JAG85185.1:CEF39517.1:P60276.1	Amidination of lysines or N-terminal amines with methyl acetimidate; Carbamidomethylation	P1:Amidination of lysines or N-terminal amines with methyl acetimidate:77.72;C5:Carbamidomethylation:1000.00;C9:Carbamidomethylation:1000.00
**PEAAPEPAAAAPER(+380.15)RR**	28,66	2068,023	16	-2,6	1035,016	33,66	0	1	11352	150211-Fr_I_Helo-red-alk-tryp-001.raw	4	4	JAW07022.1	Nucleophilic addition to cytopiloyne+H2O	R14:Nucleophilic addition to cytopiloyne+H2O:11.06
**EALAPEPAPEPAPE(+17.03)PAPEAAPEAAPEPA**	28,62	2732,318	28	2,8	1367,17	38,64	0	1	13168	150211-Fr_I_Helo-red-alk-tryp-001.raw	1	1	JAW07022.1:P86821.1	Replacement of proton with ammonium ion	E14:Replacement of proton with ammonium ion:0.00

**AEIDFSGIPE(+37.96)D**	28,5	1229,485	11	-2,2	615,7486	34,07	0	1	11504	150211-Fr_I_Helo-red-alk-tryp-001.raw	3	3	JAW06987.1:JAW07038.1:JAW06992.1:P84190.1	Replacement of proton by potassium	E10:Replacement of proton by potassium:12.28
**FC(+57.02)EDHC(+57.02)AA**	28,46	1008,343	8	-0,1	505,1787	5,73	0	1	1432	150211-Fr_I_Helo-red-alk-tryp-001.raw	1	1	P86822.2:P0C8W4.2:AFP87551.1:Q0GY42.1:ABE98265.1:JAG85223.1:ABV64385.1:ABE98262.1:Q0GY45.1:Q0GY43.1:ABE98264.1	Carbamidomethylation	C2:Carbamidomethylation:1000.00;C6:Carbamidomethylation:1000.00
**APEPAPEAAPEPVAAAAPER(-.98)**	28,44	1938,98	20	-1	970,4962	32,89	0	1	11075	150211-Fr_I_Helo-red-alk-tryp-001.raw	1	1	JAG85215.1	Amidation	R20:Amidation:1000.00
**KQNEPSFIEDS(+59.02)AIPGHQQVLDPVDLVK**	28,3	3061,554	27	1,4	1021,527	43,82	0	1	15039	150211-Fr_I_Helo-red-alk-tryp-001.raw	1	1	AIJ02110.2	Aminoethylcysteine	S11:Aminoethylcysteine:0.00
**APEPAPEPAPEAAPEAAPEPAAAAPERR(+44.03)**	28,26	2803,377	28	2	1402,699	38,6	0	1	13154	150211-Fr_I_Helo-red-alk-tryp-001.raw	1	1	JAW07022.1	Ethanolation (KR)	R28:Ethanolation (KR):0.00
**FGPNVAGETAASEVNFHFSAPHLIFPEGM(+15.99)K(+42.02)**	28,17	3258,556	30	1,3	815,6472	47,36	0	1	16288	150211-Fr_I_Helo-red-alk-tryp-001.raw	1	1	JAW07031.1:JAW06995.1	Oxidation (M); Guanidination	M29:Oxidation (M):1000.00;K30:Guanidination:1000.00
**Q(sub A)EIDFSGIPEDIIKQ**	28,03	1730,873	15	-1	866,4427	45,15	5,85E+07	1	15513	150211-Fr_I_Helo-red-alk-tryp-001.raw	39	39	JAW06987.1:JAW07038.1:JAW06992.1		
**A(+43.01)MDHEGC(+57.02)K**	27,97	989,3695	8	0	495,692	5,41	6,91E+06	1	1330	150211-Fr_I_Helo-red-alk-tryp-001.raw	3	3	JAW07016.1:JAW06979.1:P68411.1:1913435A:P68410.1:AAB36322.1:AAW72453.1:Q5G8B8.1:C0HJM9.1:JAG85185.1:CEF39517.1:P60276.1	Carbamylation; Carbamidomethylation	A1:Carbamylation:43.57;C7:Carbamidomethylation:1000.00
**ASSGYC(+57.02)AWPAC(+57.02)YC(+57.02)YGVPDHIKVWDYAT(+59.02)NK**	27,95	3497,509	29	1,7	875,386	46,56	0	1	16011	150211-Fr_I_Helo-red-alk-tryp-001.raw	1	1	JAW07016.1:JAW06979.1:P68411.1:1913435A:P68410.1:AAB36322.1	Carbamidomethylation; Aminoethylcysteine	C6:Carbamidomethylation:1000.00;C11:Carbamidomethylation:1000.00;C13:Carbamidomethylation:1000.00;T27:Aminoethylcysteine:0.00
**AEID(+15.99)FSGIPEDIIKQ**	27,89	1689,846	15	-0,7	845,9297	49,44	0	1	17002	150211-Fr_I_Helo-red-alk-tryp-001.raw	1	1	JAW06987.1:JAW07038.1:JAW06992.1	Oxidation or Hydroxylation	D4:Oxidation or Hydroxylation:61.30
**APEAAPEAAPEPAAAAPER(-.98)(+14.02)**	27,88	1827,911	19	-4,9	914,9585	19,85	0	1	6603	150211-Fr_I_Helo-red-alk-tryp-001.raw	2	2	JAW07022.1	Amidation; Methylation(KR)	R19:Amidation:1000.00;R19:Methylation(KR):1000.00
**APEAAPEPAAAAP**	27,87	1161,567	13	-0,1	581,7905	30,65	0	1	10291	150211-Fr_I_Helo-red-alk-tryp-001.raw	2	2	JAW07022.1:P86821.1:P0C8W6.1		
**EPAPEPAPEAAPEAAPEPAA**	27,86	1910,89	20	-2,9	956,4493	15,92	0	1	5116	150211-Fr_I_Helo-red-alk-tryp-001.raw	5	5	JAW07022.1:P86821.1		
**EALAPE(+37.95)PAPEP**	27,85	1157,492	11	3,2	579,755	31,72	0	1	10658	150211-Fr_I_Helo-red-alk-tryp-001.raw	1	1	JAW07022.1:P86821.1	Replacement of 2 protons by calcium	E6:Replacement of 2 protons by calcium:54.46
**AEIDFSGIPEDIIK(+163.05)QIK**	27,81	2078,076	17	3,9	1040,049	46,97	0	1	16152	150211-Fr_I_Helo-red-alk-tryp-001.raw	1	1	JAW06987.1:JAW07038.1:JAW06992.1	Phenethyl isothiocyanate	K14:Phenethyl isothiocyanate:13.38
**AEID(+21.98)FSGIPED**	27,81	1213,512	11	1,1	607,7637	34,47	0	1	11647	150211-Fr_I_Helo-red-alk-tryp-001.raw	2	2	JAW06987.1:JAW07038.1:JAW06992.1:P84190.1	Sodium adduct	D4:Sodium adduct:0.00

**C(+57.02)VEYLLSKPTASC(+57.02)IFQQC(+57.02)S**	27,64	2290,043	19	1,9	764,3564	37,86	0	1	12882	150211-Fr_I_Helo-red-alk-tryp-001.raw	1	1	AIJ02109.2	Carbamidomethylation	C1:Carbamidomethylation:1000.00;C13:Carbamidomethylation:1000.00;C18:Carbamidomethylation:1000.00
**PAPEPAPEPAPEAAPEAAPEPAAAAPERR(+79.97)RR**	27,64	3248,573	31	3,1	1083,868	41,03	0	1	14034	150211-Fr_I_Helo-red-alk-tryp-001.raw	1	1	JAW07022.1	Phosphorylation (HCDR)	R29:Phosphorylation (HCDR):0.00
**YYC(+57.02)NNAK**	27,59	931,3858	7	1,5	466,7009	5,57	0	1	1379	150211-Fr_I_Helo-red-alk-tryp-001.raw	19	19	AIJ02110.2:JAG85213.1	Carbamidomethylation	C3:Carbamidomethylation:1000.00
**ESLIPGIDTIF**	27,45	1203,639	11	-1,7	602,8256	49,1	0	1	16883	150211-Fr_I_Helo-red-alk-tryp-001.raw	2	2	AMO02560.1		
**AAPEAAPEPAAAAPER(-.98)(+14.02)**	27,41	1530,779	16	-4,7	766,3931	39,18	0	1	13362	150211-Fr_I_Helo-red-alk-tryp-001.raw	1	1	JAW07022.1	Amidation; Methylation(KR)	R16:Amidation:1000.00;R16:Methylation(KR):1000.00
**G(sub R)LTSQSEYAC(+57.02)PAIDK**	27,04	1638,756	15	-0,1	820,3851	30,83	0	1	10352	150211-Fr_I_Helo-red-alk-tryp-001.raw	8	8	P86822.2:P0C8W4.2:AFP87551.1	Carbamidomethylation	C10:Carbamidomethylation:1000.00
**R(-42.02)LTSQSEYAC(+57.02)PAIDKFC(+57.02)EDHC(+57.02)AA**	27,02	2686,146	23	-0,5	896,3889	34,21	0	1	11554	150211-Fr_I_Helo-red-alk-tryp-001.raw	1	1	P86822.2:P0C8W4.2:AFP87551.1	Ornithine from Arginine; Carbamidomethylation	R1:Ornithine from Arginine:1000.00;C10:Carbamidomethylation:1000.00;C17:Carbamidomethylation:1000.00;C21:Carbamidomethylation:1000.00
**C(+57.02)NC(+57.02)IKL**	26,99	806,3779	6	-0,5	404,196	23,94	0	1	8095	150211-Fr_I_Helo-red-alk-tryp-001.raw	17	17	P86822.2:Q0GY42.1:ABE98265.1:JAG85223.1:ABV64385.1:ABE98262.1:Q0GY45.1	Carbamidomethylation	C1:Carbamidomethylation:1000.00;C3:Carbamidomethylation:1000.00
**APEPAPEAAPE(+37.95)AAPEPAA**	26,97	1722,741	18	0,5	575,2546	32,52	0	1	10943	150211-Fr_I_Helo-red-alk-tryp-001.raw	1	1	JAW07022.1:P86821.1	Replacement of 2 protons by calcium	E11:Replacement of 2 protons by calcium:22.72
**AEIDFSGIPEDIIK(+136.03)QI**	26,95	1922,964	16	-0,9	641,9948	50,25	0	1	17275	150211-Fr_I_Helo-red-alk-tryp-001.raw	1	1	JAW06987.1:JAW07038.1:JAW06992.1	O-Diethylphosphorylation	K14:O-Diethylphosphorylation:64.00
**ALAPEPAPEPAPE(+17.03)PAPEA**	26,95	1769,883	18	-0,2	885,9488	34,02	0	1	11483	150211-Fr_I_Helo-red-alk-tryp-001.raw	24	24	JAW07022.1:P86821.1	Replacement of proton with ammonium ion	E13:Replacement of proton with ammonium ion:0.00
**ADVDFTGIADSIIKKIKETNAKPPAR(+212.01)**	26,93	3009,542	26	-0,3	1505,778	38,33	0	1	13060	150211-Fr_I_Helo-red-alk-tryp-001.raw	1	1	JAW07002.1	phosphate-ribosylation	R26:phosphate-ribosylation:0.00
**AEIDFSGIPEDIIKQIKETN(-17.03)AKPPARFDP**	26,9	3221,661	29	1,7	1611,84	39,29	0	1	13402	150211-Fr_I_Helo-red-alk-tryp-001.raw	1	1	JAW06987.1:JAW07038.1:JAW06992.1	Ammonia-loss (N)	N20:Ammonia-loss (N):1000.00
**SC(+57.02)PYDDGYIMGSTIE(+17.03)PN**	26,88	1934,803	17	-0,9	968,4077	35,63	0	1	12060	150211-Fr_I_Helo-red-alk-tryp-001.raw	1	1	JAG85190.1	Carbamidomethylation; Replacement of proton with ammonium ion	C2:Carbamidomethylation:1000.00;E15:Replacement of proton with ammonium ion:16.78
**KGSSGYC(+57.02)AWPAC(+57.02)YC(+57.02)YGLPNWVK(+411.26)**	26,83	3034,404	22	-4,3	1012,471	51,93	0	1	17833	150211-Fr_I_Helo-red-alk-tryp-001.raw	1	1	AAW72453.1:Q5G8B8.1:AAB29128.1:P56612.1:AIA81145.1:CAA46982.1:P15226.2:P0DMM8.1:1NPI:1B7D:AAB36321.1:AAB36318.1:P56611.1:JAG85191.1:JAG85210.1	Carbamidomethylation; LeudimethylArgGlyGly	C7:Carbamidomethylation:1000.00;C12:Carbamidomethylation:1000.00;C14:Carbamidomethylation:1000.00;K22:LeudimethylArgGlyGly:47.28

**PEAAPEPAAAAPER(+80.99)R**	26,77	1612,759	15	-1,5	807,3857	18,5	0	1	6091	150211-Fr_I_Helo-red-alk-tryp-001.raw	6	6	JAW07022.1	Arginine replacement by Nitropyrimidyl ornithine	R14:Arginine replacement by Nitropyrimidyl ornithine:0.00
**GSDVTGGFPVNSNNC(+57.02)IY(-94.04)PC(+57.02)YSTQDEIQC(+57.02)EEFC(+57.02)EKL**	26,76	4022,67	35	-3,4	1341,893	44,57	0	1	15309	150211-Fr_I_Helo-red-alk-tryp-001.raw	1	1	JAW07006.1	Carbamidomethylation; Dehydroalanine (Y)	C15:Carbamidomethylation:1000.00;Y17:Dehydroalanine (Y):0.00;C19:Carbamidomethylation:1000.00;C28:Carbamidomethylation:1000.00;C32:Carbamidomethylation:1000.00
**APEAAPEAAPEPA**	26,75	1219,572	13	-0,2	610,7932	30,41	0	1	10208	150211-Fr_I_Helo-red-alk-tryp-001.raw	8	8	JAW07022.1:P86821.1		
**PEPAAPEPAPEAAPEPVAA**	26,73	1809,878	19	-0,5	905,946	34,85	0	1	11783	150211-Fr_I_Helo-red-alk-tryp-001.raw	1	1	JAG85215.1		
**AEIDFS(+162.08)GIPEDIIK**	26,69	1707,874	14	-1	854,9432	48,72	0	1	16753	150211-Fr_I_Helo-red-alk-tryp-001.raw	1	1	JAW06987.1:JAW07038.1:JAW06992.1:P84190.1	O-Pinacolylmethylphosphonylation	S6:O-Pinacolylmethylphosphonylation:24.61
**ITC(+57.02)FLTAAGYC(+57.02)NTEC(+57.02)TLK(+43.01)K(+14.02)**	26,68	2307,07	19	-0,2	770,0304	20,4	0	1	6810	150211-Fr_I_Helo-red-alk-tryp-001.raw	1	1	AAC25688.1:O77463.1:AAC25689.1	Carbamidomethylation; Carbamylation; Methylation(KR)	C3:Carbamidomethylation:1000.00;C11:Carbamidomethylation:1000.00;C15:Carbamidomethylation:1000.00;K18:Carbamylation:0.00;K19:Methylation(KR):0.00
**LAPEPAPEPAPEPAPEAAPE(+17.03)AAPEPAA**	26,67	2603,275	27	-1,1	868,7648	37,16	2,39E+09	1	12624	150211-Fr_I_Helo-red-alk-tryp-001.raw	2	2	JAW07022.1:P86821.1	Replacement of proton with ammonium ion	E20:Replacement of proton with ammonium ion:22.99
**AEIDFSGIPEDIIK(+149.03)QIK**	26,64	2064,06	17	3,1	1033,041	48,49	0	1	16674	150211-Fr_I_Helo-red-alk-tryp-001.raw	1	1	JAW06987.1:JAW07038.1:JAW06992.1	Benzyl isothiocyanate	K14:Benzyl isothiocyanate:7.77
**SGIPEDIIK**	26,63	970,5334	9	0,1	486,274	30,83	0	1	10353	150211-Fr_I_Helo-red-alk-tryp-001.raw	4	4	JAW06987.1:JAW07038.1:JAW06992.1:P84190.1		
**AEIDFSGIPEDIIKQIK(+188.03)ETNAKPPAR**	26,58	3067,572	26	-1,3	1023,53	38,49	0	1	13115	150211-Fr_I_Helo-red-alk-tryp-001.raw	2	2	JAW06987.1:JAW07038.1:JAW06992.1	Lipoyl	K17:Lipoyl:0.00
**EPS(+79.97)Y(+79.97)IKESDVQNGK**	26,57	1752,701	14	-3,1	877,355	11,1	0	1	3378	150211-Fr_I_Helo-red-alk-tryp-001.raw	3	3	JAW07039.1:JAG85190.1:JAW07042.1	Phosphorylation (STY)	S3:Phosphorylation (STY):14.62;Y4:Phosphorylation (STY):16.51
**PEAAPEAAPEPAAAAPER(-.98)(+14.02)**	26,56	1756,874	18	-4,2	879,4407	23,5	0	1	7939	150211-Fr_I_Helo-red-alk-tryp-001.raw	1	1	JAW07022.1	Amidation; Methylation(KR)	R18:Amidation:1000.00;R18:Methylation(KR):1000.00
**LSSW(sub A)ANMMQMEWDDELAAVAQAHANQC(+57.02)K**	26,54	3234,399	28	0,5	647,8875	33,88	0	1	11435	150211-Fr_I_Helo-red-alk-tryp-001.raw	1	1	JAW07031.1:JAW06995.1	Carbamidomethylation	C27:Carbamidomethylation:1000.00
**EPAAAAPEAAPE(+43.99)**	26,49	1166,509	12	-3,4	584,2598	23,9	0	1	8081	150211-Fr_I_Helo-red-alk-tryp-001.raw	5	5	P0C8W6.1	Carboxylation (E)	E12:Carboxylation (E):0.00
**KEALAPEPAPKP(+31.99)APEPAPEPA**	26,43	2138,089	21	-1,8	713,7025	32,05	0	1	10774	150211-Fr_I_Helo-red-alk-tryp-001.raw	1	1	P0C8W6.1	Dihydroxy	P12:Dihydroxy:0.00
**ESDVQNGK**	26,41	875,3984	8	0,3	438,7066	5,56	0	1	1375	150211-Fr_I_Helo-red-alk-tryp-001.raw	1	1	JAW07039.1:JAG85190.1:JAW07042.1		
**APEPAAAAPEAAPE**	26,37	1290,609	14	-0,1	646,3118	58,3	1,02E+07	1	19920	150211-Fr_I_Helo-red-alk-tryp-001.raw	49	49	P0C8W6.1		
**ADVDFTGIAD(-18.01)SIIK(-.98)**	26,36	1444,756	14	-0,4	723,385	12,44	0	1	3848	150211-Fr_I_Helo-red-alk-tryp-001.raw	5	5	JAW07002.1:P86824.1	Dehydration; Amidation	D10:Dehydration:0.00;K14:Amidation:1000.00

**E(+27.99)ALAPEPAPEPAPEPA**	26,31	1612,762	16	-3,1	807,3857	7,78	0	1	2174	150211-Fr_I_Helo-red-alk-tryp-001.raw	12	12	P86821.1	Formylation (Protein N-term)	E1:Formylation (Protein N-term):1000.00
**ALAPEPAPEPAP**	26,29	1158,592	12	0,8	580,3038	32,2	0	1	10827	150211-Fr_I_Helo-red-alk-tryp-001.raw	1	1	JAW07022.1:P86821.1		
**GY(sub S)SGYCAWPAC(+57.02)YC(+57.02)YGLPDSVK**	26,29	2415,996	21	5,1	806,3435	41,22	0	1	14102	150211-Fr_I_Helo-red-alk-tryp-001.raw	1	1	AAC25688.1:O77463.1:AAC25689.1	Carbamidomethylation	C11:Carbamidomethylation:1000.00;C13:Carbamidomethylation:1000.00
**Q(sub A)DVDFTGIADSIIL(sub K)K**	26,28	1633,856	15	0,7	817,9359	49,38	0	1	16983	150211-Fr_I_Helo-red-alk-tryp-001.raw	3	3	JAW07002.1		
**NVLVIAPPD(+17.03)PSVFSDPD**	26,25	1797,915	17	0,7	899,9653	47,42	0	1	16310	150211-Fr_I_Helo-red-alk-tryp-001.raw	1	1	AIJ02118.2	Replacement of proton with ammonium ion	D9:Replacement of proton with ammonium ion:9.87
**ASSM(sub G)YCAWPAC(+57.02)YC(+57.02)YGVPDHIK**	26,25	2478,027	21	0,9	827,0169	36,09	0	1	12229	150211-Fr_I_Helo-red-alk-tryp-001.raw	2	2	JAW07016.1:JAW06979.1:P68411.1:1913435A:P68410.1:AAB36322.1	Carbamidomethylation	C11:Carbamidomethylation:1000.00;C13:Carbamidomethylation:1000.00
**G(sub K)SSLLFLTPIFVK**	26,19	1420,833	13	-0,5	711,4233	50,91	0	1	17493	150211-Fr_I_Helo-red-alk-tryp-001.raw	1	1	JAW07036.1		
**G(sub K)YLFLTDSNFR**	26,15	1331,651	11	-0,8	666,8322	35,65	0	1	12068	150211-Fr_I_Helo-red-alk-tryp-001.raw	4	4	AIJ02112.2		
**APEPAPEAAPEAAPE(+37.03)PAAAAPE**	26,14	2089,996	22	2,3	697,6741	33,74	0	1	11381	150211-Fr_I_Helo-red-alk-tryp-001.raw	1	1	JAW07022.1:P86821.1	Propargylamine	E15:Propargylamine:18.48
**LTS(+59.02)QSEYAC(+57.02)PAIDKFC(+57.02)EDHC(+57.02)AAK**	26,06	2759,181	23	2,2	690,8041	18,51	0	1	6094	150211-Fr_I_Helo-red-alk-tryp-001.raw	9	9	P86822.2:P0C8W4.2:AFP87551.1	Aminoethylcysteine; Carbamidomethylation	S3:Aminoethylcysteine:0.00;C9:Carbamidomethylation:1000.00;C16:Carbamidomethylation:1000.00;C20:Carbamidomethylation:1000.00
**Q(sub A)EIDY(sub F)SGIPEDIIK**	26,05	1618,809	14	-1,8	810,4103	39,8	0	1	13587	150211-Fr_I_Helo-red-alk-tryp-001.raw	5	5	JAW06987.1:JAW07038.1:JAW06992.1		
**DQ(sub A)DIIMLISNR**	26	1316,676	11	-0,2	659,345	35,91	0	1	12164	150211-Fr_I_Helo-red-alk-tryp-001.raw	1	1	AIJ02110.2:JAG85213.1		
**TNEALTEYVTQ(+.98)MFTGVQNMYDTM(+15.99)NLDIKI**	25,98	3399,556	29	-0,8	1134,192	49,16	0	1	16906	150211-Fr_I_Helo-red-alk-tryp-001.raw	1	1	JAG85190.1	Deamidation (NQ); Oxidation (M)	Q11:Deamidation (NQ):1.44;M23:Oxidation (M):11.12
**AEIDFSGIPED(+356.19)IIK**	25,96	1901,981	14	-0,6	951,9971	50,78	0	1	17450	150211-Fr_I_Helo-red-alk-tryp-001.raw	1	1	JAW06987.1:JAW07038.1:JAW06992.1:P84190.1	Biotin polyethyleneoxide amine	D11:Biotin polyethyleneoxide amine:0.00
**DQ(sub A)C(+57.02)YC(+57.02)EHLPESVK**	25,96	1663,697	13	-1,6	832,8544	30,89	0	1	10373	150211-Fr_I_Helo-red-alk-tryp-001.raw	1	1	JAW07006.1	Carbamidomethylation	C3:Carbamidomethylation:1000.00;C5:Carbamidomethylation:1000.00
**APEPAPEAAPEAAPE(+37.95)PAAAAPE**	25,87	2090,911	22	-0,3	697,9774	75,04	0	1	25292	150211-Fr_I_Helo-red-alk-tryp-001.raw	1	1	JAW07022.1:P86821.1	Replacement of 2 protons by calcium	E15:Replacement of 2 protons by calcium:8.15
**H(sub A)H(sub E)IDFSGIPEDIIK**	25,85	1619,831	14	1,2	810,9236	48,6	0	1	16712	150211-Fr_I_Helo-red-alk-tryp-001.raw	1	1	P84190.1		
**PEPAPEPAPEAAPEAAPEPAAAAPER(+144.04)RR**	25,81	2988,458	28	4,5	997,1642	39,11	0	1	13336	150211-Fr_I_Helo-red-alk-tryp-001.raw	1	1	JAW07022.1	Condensation product of 3-deoxyglucosone	R26:Condensation product of 3-deoxyglucosone:0.00
**APEPAAAAPE**	25,77	922,4396	10	-0,4	462,2269	15,75	7,78E+07	1	5054	150211-Fr_I_Helo-red-alk-tryp-001.raw	73	73	JAW07022.1:P86821.1:P0C8W6.1		

**EIDFS(+121.04)GIPEDIIK**	25,57	1595,791	13	-1,1	798,9017	38,32	0	1	13057	150211-Fr_I_Helo-red-alk-tryp-001.raw	1	1	JAW06987.1:JAW07038.1:JAW06992.1:P84190.1	Phosphorylation to pyridyl thiol	S5:Phosphorylation to pyridyl thiol:1000.00
**LTSESEYAC(+57.02)PAIDKFC(+57.02)EDHC(+57.02)AAK**	25,52	2701,146	23	5,4	1351,587	15,33	0	1	4900	150211-Fr_I_Helo-red-alk-tryp-001.raw	2	2	Q0GY42.1:ABE98265.1:JAG85223.1	Carbamidomethylation	C9:Carbamidomethylation:1000.00;C16:Carbamidomethylation:1000.00;C20:Carbamidomethylation:1000.00
**SLAHEAAHLLAVP(+29.04)HDGDDFKVS**	25,5	2357,189	22	-1,1	590,3039	35,91	0	1	12161	150211-Fr_I_Helo-red-alk-tryp-001.raw	3	3	AIJ02111.3	Dimethylation of proline residue	P13:Dimethylation of proline residue:1000.00
**LIPNDQLR**	25,49	967,545	8	0,1	484,7798	25,92	0	1	8755	150211-Fr_I_Helo-red-alk-tryp-001.raw	18	18	P0C2F3.1:P69940.2:Q5G8A6.1:JAG85216.1:AAW72464.1:AAW72465.1		
**PEAAPEPAAAAPER(+44.03)**	25,42	1419,699	14	4,7	710,8603	11,58	0	1	3547	150211-Fr_I_Helo-red-alk-tryp-001.raw	3	3	JAW07022.1	Ethanolation (KR)	R14:Ethanolation (KR):1000.00
**LTSQSEYAC(+57.02)PAIEK(+43.01)**	25,4	1638,756	14	-0,1	820,3851	30,65	0	1	10290	150211-Fr_I_Helo-red-alk-tryp-001.raw	1	1	ABV64385.1:ABE98262.1:Q0GY45.1	Carbamidomethylation; Carbamylation	C9:Carbamidomethylation:1000.00;K14:Carbamylation:39.87
**APEAAPEAAPEP(+15.99)AAAAPE**	25,38	1674,774	18	-0,7	838,3935	11,33	0	1	3459	150211-Fr_I_Helo-red-alk-tryp-001.raw	1	1	JAW07022.1:P86821.1	Oxidation or Hydroxylation	P12:Oxidation or Hydroxylation:0.00
**APEPAPEPAPEPAPEA**	25,37	1568,736	16	-0,9	785,3745	32,25	0	1	10846	150211-Fr_I_Helo-red-alk-tryp-001.raw	7	7	JAW07022.1:P86821.1:P0C8W6.1		
**KVTCFLTAAGYC(+57.02)NTEC(+57.02)T(sub K)LQK**	25,28	2307,07	20	-0,5	1154,542	20,46	0	1	6832	150211-Fr_I_Helo-red-alk-tryp-001.raw	2	2	AAW72463.1:Q5G8A8.1	Carbamidomethylation	C12:Carbamidomethylation:1000.00;C16:Carbamidomethylation:1000.00
**QIK(+114.04)ETNAKPPA**	25,22	1309,699	11	0,4	437,5738	5,78	0	1	1450	150211-Fr_I_Helo-red-alk-tryp-001.raw	1	1	JAW06987.1:JAW07038.1:JAW06992.1	Ubiquitin	K3:Ubiquitin:0.00
**IDFSGIPEDIIKR(sub E)**	25,21	1501,814	13	-0,2	501,6118	35,95	0	1	12179	150211-Fr_I_Helo-red-alk-tryp-001.raw	1	1	P84190.1		
**AAGYC(+57.02)NTEC(+57.02)TLK**	25,2	1386,591	12	-2	694,3013	5,83	0	1	1470	150211-Fr_I_Helo-red-alk-tryp-001.raw	1	1	AAC25688.1:O77463.1:AAC25689.1	Carbamidomethylation	C5:Carbamidomethylation:1000.00;C9:Carbamidomethylation:1000.00
**GIPEDIIK**	25,13	883,5015	8	0,5	442,7582	30,23	0	1	10148	150211-Fr_I_Helo-red-alk-tryp-001.raw	24	24	JAW06987.1:JAW07038.1:JAW06992.1:P84190.1		
**AEIDFSGIPEDIIK(+117.02)QIK**	25,12	2032,055	17	-3,4	678,3566	49,27	0	1	16944	150211-Fr_I_Helo-red-alk-tryp-001.raw	1	1	JAW06987.1:JAW07038.1:JAW06992.1	N-Homocysteine thiolactone	K14:N-Homocysteine thiolactone:0.00
**IKETNAKPPA**	25,08	1067,597	10	0,1	534,806	11,17	0	1	3404	150211-Fr_I_Helo-red-alk-tryp-001.raw	43	43	JAW06987.1:JAW07038.1:JAW06992.1:P84190.1:JAW07002.1		
**AEIDFSGIPEDIIKQIKEL(sub T)**	24,98	2157,157	19	-0,9	720,0589	51,74	8,69E+08	1	17772	150211-Fr_I_Helo-red-alk-tryp-001.raw	8	8	JAW06987.1:JAW07038.1:JAW06992.1		
**APEPAPEPAPEP**	24,97	1200,566	12	1,2	601,2911	31,11	0	1	10449	150211-Fr_I_Helo-red-alk-tryp-001.raw	2	2	JAW07022.1:P86821.1:P0C8W6.1		
**APEPAPEAAPEAAPEP**	24,91	1542,72	16	0,2	772,3675	14,23	0	1	4494	150211-Fr_I_Helo-red-alk-tryp-001.raw	3	3	JAW07022.1:P86821.1		
**KELPEDIK**	24,87	970,5334	8	-1,1	486,2735	27,19	0	1	9162	150211-Fr_I_Helo-red-alk-tryp-001.raw	52	52			
**A(+380.15)C(+57.02)PAIDKFC(+57.02)EDHC(+57.02)AA**	24,84	2143,854	15	-3,9	1072,93	15,65	0	1	5014	150211-Fr_I_Helo-red-alk-tryp-001.raw	5	5	P86822.2:P0C8W4.2:AFP87551.1:Q0GY42.1:ABE98265.1:JAG85223.1	Nucleophilic addition to cytopiloyne+H2O; Carbamidomethylation	A1:Nucleophilic addition to cytopiloyne+H2O:53.33;C2:Carbamidomethylation:1000.00;C9:Carbamidomethylation:1000

															.00;C13:Carbamidomethylation:1000.00
**PAPEAAPEAAPEPAAAAPER(+199.07)**	24,75	2110,999	20	-4,9	1056,502	33,98	0	1	11469	150211-Fr_I_Helo-red-alk-tryp-001.raw	2	2	JAW07022.1	Oxidized Arginine biotinylated with biotin hydrazide	R20:Oxidized Arginine biotinylated with biotin hydrazide:1000.00
**IW(+13.98)TSETNK**	24,71	991,4611	8	-1,5	496,7371	5,81	0	1	1460	150211-Fr_I_Helo-red-alk-tryp-001.raw	1	1	AAC25688.1:O77463.1:AAC25689.1	Tryptophan oxidation to oxolactone	W2:Tryptophan oxidation to oxolactone:1000.00
**PEPAPEAAPEAAPEPAAAAPER(+44.03)**	24,6	2182,054	22	1,8	1092,036	14,55	0	1	4612	150211-Fr_I_Helo-red-alk-tryp-001.raw	1	1	JAW07022.1	Ethanolation (KR)	R22:Ethanolation (KR):1000.00
**APEPAPEPAPEAAPEAAPE(+129.04)PAAAAPERRR**	24,57	3044,495	29	0,8	1015,84	41,47	0	1	14192	150211-Fr_I_Helo-red-alk-tryp-001.raw	1	1	JAW07022.1	Monoglutamyl	E19:Monoglutamyl:0.00
**PEPAPEAAPEPVAAAAPER(-.98)**	24,56	1867,943	19	1,1	934,9797	37,9	9,36E+08	1	12897	150211-Fr_I_Helo-red-alk-tryp-001.raw	1	1	JAG85215.1	Amidation	R19:Amidation:1000.00
**LAPEPAPEPAP**	24,49	1087,555	11	0,2	544,7849	31,42	0	1	10555	150211-Fr_I_Helo-red-alk-tryp-001.raw	2	2	JAW07022.1:P86821.1		
**PEAAPEPAAAAPERRR(+380.15)**	24,48	2068,023	16	-2,4	690,3464	33,81	0	1	11407	150211-Fr_I_Helo-red-alk-tryp-001.raw	2	2	JAW07022.1	Nucleophilic addition to cytopiloyne+H2O	R16:Nucleophilic addition to cytopiloyne+H2O:0.00
**WADQ(sub E)T(sub V)LNLDDFETIR**	24,47	1835,869	15	4	918,9454	47,34	0	1	16282	150211-Fr_I_Helo-red-alk-tryp-001.raw	1	1	JAW07036.1:JAW06986.1		
**ITC(+57.02)FLT(-18.01)AAGYC(+57.02)NTEC(+57.02)TLK**	24,45	2103,943	18	-0,4	1052,978	47,54	0	1	16352	150211-Fr_I_Helo-red-alk-tryp-001.raw	1	1	AAC25688.1:O77463.1:AAC25689.1	Carbamidomethylation; Dehydration	C3:Carbamidomethylation:1000.00;T6:Dehydration:0.00;C11:Carbamidomethylation:1000.00;C15:Carbamidomethylation:1000.00
**E(sub A)IITVVDNGIMIGC(+57.02)GIFDVASPSVK**	24,44	2633,345	25	5,3	878,7934	49,05	0	1	16867	150211-Fr_I_Helo-red-alk-tryp-001.raw	1	1	JAW07019.1:JAW06978.1	Carbamidomethylation	C14:Carbamidomethylation:1000.00
**AEIDFSGIPEDIIKQIK(+14.02)ET(-.98)**	24,43	2158,152	19	1,4	720,3923	50	0	1	17192	150211-Fr_I_Helo-red-alk-tryp-001.raw	4	4	JAW06987.1:JAW07038.1:JAW06992.1	Methylation(KR); Amidation	K17:Methylation(KR):0.00;T19:Amidation:1000.00
**DADIIMLTTDRPLADIS(+58.03)SEGK**	24,37	2318,155	21	-3,3	773,7231	34,16	0	1	11534	150211-Fr_I_Helo-red-alk-tryp-001.raw	8	8	AIJ02113.2	2 3-dihydro-2 2-dimethyl-7-benzofuranol N-methyl carbamate	S17:2 3-dihydro-2 2-dimethyl-7-benzofuranol N-methyl carbamate:14.02
**ADVDFTGIADS(-18.01)IIK(-.98)**	24,37	1444,756	14	0,2	723,3855	11,76	0	1	3611	150211-Fr_I_Helo-red-alk-tryp-001.raw	3	3	JAW07002.1:P86824.1	Dehydration; Amidation	S11:Dehydration:0.00;K14:Amidation:1000.00
**EIDFS(+340.09)GIPEDIIK**	24,36	1814,841	13	1	908,4288	48,22	0	1	16582	150211-Fr_I_Helo-red-alk-tryp-001.raw	1	1	JAW06987.1:JAW07038.1:JAW06992.1:P84190.1	Phosphopantetheine	S5:Phosphopantetheine:1000.00
**KEALAPEPAPK(+170.04)PAPEP**	24,34	1810,914	16	0,9	906,4651	33,27	0	1	11213	150211-Fr_I_Helo-red-alk-tryp-001.raw	1	1	P0C8W6.1	Menadione quinone derivative	K11:Menadione quinone derivative:84.36
**DGLCAFLIGR**	24,19	1063,549	10	3,6	532,7834	32,55	0	1	10953	150211-Fr_I_Helo-red-alk-tryp-001.raw	1	1	JAG85161.1		
**KKQNEPSFIEDSAIPGHQQVLDPVDLVK**	24,08	3130,63	28	4,2	1044,555	38,03	0	1	12947	150211-Fr_I_Helo-red-alk-tryp-001.raw	1	1	AIJ02110.2		
**EALAPE(+17.03)PAPEPAPEP**	24,08	1530,757	15	-0,7	766,3849	26,08	0	1	8807	150211-Fr_I_Helo-red-alk-tryp-001.raw	1	1	JAW07022.1:P86821.1	Replacement of proton with ammonium ion	E6:Replacement of proton with ammonium ion:6.29
**ITC(+57.02)FLTAAGYC(+57.02)NTEC(+57.02)TLG(sub K)K**	23,96	2178,975	19	-2,5	1090,492	35,51	0	1	12018	150211-Fr_I_Helo-red-alk-tryp-001.raw	1	1	AAC25688.1:O77463.1:AAC25689.1	Carbamidomethylation	C3:Carbamidomethylation:1000.00;C11:Carbamidomethylati

															on:1000.00;C15:Carbamidomethylation:1000.00
**APEAAPEPAAAAPE(+17.03)**	23,94	1307,636	14	-0,3	654,8249	15,67	7,98E+08	1	5021	150211-Fr_I_Helo-red-alk-tryp-001.raw	127	127	JAW07022.1:P86821.1:P0C8W6.1	Replacement of proton with ammonium ion	E14:Replacement of proton with ammonium ion:47.00
**LAPEPAPEPAPE(+21.98)PAPEA**	23,92	1703,802	17	1,5	852,9094	34,21	0	1	11555	150211-Fr_I_Helo-red-alk-tryp-001.raw	1	1	JAW07022.1:P86821.1	Sodium adduct	E12:Sodium adduct:11.12
**EALAPEPAPE(+17.03)P**	23,87	1136,571	11	0,7	569,2933	31,97	0	1	10745	150211-Fr_I_Helo-red-alk-tryp-001.raw	2	2	JAW07022.1:P86821.1	Replacement of proton with ammonium ion	E10:Replacement of proton with ammonium ion:0.00
**EALAPE(+17.03)PAPEPAPEPAPEA**	23,83	1898,926	19	-1,3	950,4691	74,89	0	1	25246	150211-Fr_I_Helo-red-alk-tryp-001.raw	1	1	JAW07022.1:P86821.1	Replacement of proton with ammonium ion	E6:Replacement of proton with ammonium ion:0.00
**PEPAPKPAPEPAPEPAPEPAPEAAPEPAAAAPE**	23,82	3189,551	33	0,5	1064,191	43,42	0	1	14895	150211-Fr_I_Helo-red-alk-tryp-001.raw	16	16	P0C8W6.1		
**GSSGYC(+57.02)AWPAC(+57.02)YC(+57.02)YGLP(-27.99)DSVP**	23,69	2337,949	21	2,5	1169,985	44,28	0	1	15207	150211-Fr_I_Helo-red-alk-tryp-001.raw	1	1		Carbamidomethylation; Pyrrolidone from Proline	C6:Carbamidomethylation:1000.00;C11:Carbamidomethylation:1000.00;C13:Carbamidomethylation:1000.00;P17:Pyrrolidone from Proline:0.00
**KKVNEPSFIGDS(+86.00)AILGHQQVLDPVDLVK**	23,68	3131,65	28	-4,7	627,3344	37,84	0	1	12877	150211-Fr_I_Helo-red-alk-tryp-001.raw	2	2	JAG85213.1	Malonylation	S12:Malonylation:0.00
**K(+43.01)(+14.02)EGYAMDHEGC(+57.02)K**	23,61	1480,607	12	-1	741,3102	6,97	3,49E+06	1	1877	150211-Fr_I_Helo-red-alk-tryp-001.raw	2	2	P68411.1:1913435A:P68410.1:AAB36322.1:AAW72453.1:Q5G8B8.1:P60276.1	Carbamylation; Methylation(KR); Carbamidomethylation	K1:Carbamylation:48.34;K1:Methylation(KR):50.04;C11:Carbamidomethylation:1000.00
**AEIDFSGIPED(+17.03)II**	23,49	1434,724	13	-1	718,3687	46,93	0	1	16139	150211-Fr_I_Helo-red-alk-tryp-001.raw	2	2	JAW06987.1:JAW07038.1:JAW06992.1:P84190.1	Replacement of proton with ammonium ion	D11:Replacement of proton with ammonium ion:0.00
**AQVIGITPF(+21.98)**	23,41	966,515	9	-0,1	484,2647	32,92	0	1	11084	150211-Fr_I_Helo-red-alk-tryp-001.raw	1	1	AIJ02110.2:JAG85213.1	Sodium adduct	F9:Sodium adduct:1000.00
**EAAPEPAAAAPEAAPE**	23,38	1490,689	16	-2	746,3502	13,15	0	1	4106	150211-Fr_I_Helo-red-alk-tryp-001.raw	2	2	P0C8W6.1		
**A(+88.00)EIDFSGIPEDIIK**	23,36	1633,791	14	1	817,9035	65,52	0	1	22226	150211-Fr_I_Helo-red-alk-tryp-001.raw	1	1	JAW06987.1:JAW07038.1:JAW06992.1:P84190.1	3-sulfanylpropanoyl	A1:3-sulfanylpropanoyl:39.02
**AEIDFSGIPEDIIKQIKETNAKPPAR(+132.02)**	23,35	3011,56	26	-3,5	1004,857	38,49	0	1	13114	150211-Fr_I_Helo-red-alk-tryp-001.raw	3	3	JAW06987.1:JAW07038.1:JAW06992.1	Hydroxyphenylglyoxal arginine	R26:Hydroxyphenylglyoxal arginine:1000.00
**APEAAPEAAPEPAAAAPE(+453.21)**	23,31	2111,991	18	0,4	1057,003	16,56	0	1	5353	150211-Fr_I_Helo-red-alk-tryp-001.raw	1	1	JAW07022.1:P86821.1	Puromycin	E18:Puromycin:1000.00
**A(+164.06)NLGEFPWMVFIR**	23,3	1742,862	13	3,7	872,4415	56,9	0	1	19467	150211-Fr_I_Helo-red-alk-tryp-001.raw	1	1	JAG85164.1	O-Diisopropylphosphorylation	A1:O-Diisopropylphosphorylation:1000.00
**GSSGYC(+57.02)AWPAC(+57.02)YC(+57.02)YGLPD(+17.03)**	23,22	2099,818	18	0	1050,916	44,64	0	1	15331	150211-Fr_I_Helo-red-alk-tryp-001.raw	1	1	AAC25688.1:O77463.1:AAC25689.1	Carbamidomethylation; Replacement of proton with ammonium ion	C6:Carbamidomethylation:1000.00;C11:Carbamidomethylation:1000.00;C13:Carbamidomethylation:1000.00;D18:Replacement of proton with ammonium ion:1000.00
**PSFIEDSAIPGHQQVLDPVDLVK(+127.06)NM**	23,21	2875,443	25	-0,8	959,4874	48,28	0	1	16604	150211-Fr_I_Helo-red-alk-tryp-001.raw	8	8	AIJ02110.2	N-Succinimidyl-2-morpholine acetate	K23:N-Succinimidyl-2-morpholine acetate:23.92

**APEAAPEAAPE(+17.03)PAAAAPE**	23,2	1675,805	18	0,2	838,91	27,39	0	1	9227	150211-Fr_I_Helo-red-alk-tryp-001.raw	3	3	JAW07022.1:P86821.1	Replacement of proton with ammonium ion	E11:Replacement of proton with ammonium ion:0.00
**EALAPEPAPK(+218.17)PAPEPAPEPAP**	23,17	2293,224	21	-4,9	765,4116	33,63	0	1	11340	150211-Fr_I_Helo-red-alk-tryp-001.raw	1	1	P0C8W6.1	Michael addition of BHT quinone methide	K10:Michael addition of BHT quinone methide:1000.00
**R(sub A)EN(sub I)DFSGIPEDIIK**	23,16	1631,815	14	-0,7	816,9144	46,72	0	1	16065	150211-Fr_I_Helo-red-alk-tryp-001.raw	1	1	P84190.1		
**KEALAPEPAPE(+21.98)P**	23,11	1269,622	12	-0,5	635,8177	13,2	0	1	4123	150211-Fr_I_Helo-red-alk-tryp-001.raw	3	3	JAW07022.1:P86821.1	Sodium adduct	E11:Sodium adduct:24.44
**S(+71.04)LEDEQENMEERAEIDFSGIPEDIIK**	23,08	3106,429	26	-4,5	1036,479	49,2	0	1	16920	150211-Fr_I_Helo-red-alk-tryp-001.raw	1	1	JAW06987.1:JAW07038.1:JAW06992.1	Propionamide (K X@N-term)	S1:Propionamide (K X@N-term):39.42
**ADVDFTGIADSIIKKIKE(+212.01)TNAKPPAR**	23,08	3009,542	26	0,5	1004,189	38,26	0	1	13032	150211-Fr_I_Helo-red-alk-tryp-001.raw	2	2	JAW07002.1	phosphate-ribosylation	E18:phosphate-ribosylation:0.00
**PEAAPEPAAAAPER(+14.02)RR**	23,06	1701,891	16	0,1	851,9529	36,85	0	1	12511	150211-Fr_I_Helo-red-alk-tryp-001.raw	1	1	JAW07022.1	Methylation(KR)	R14:Methylation(KR):0.00
**KC(+57.02)DDFK**	23,06	811,3534	6	0,3	406,6841	5,69	0	1	1420	150211-Fr_I_Helo-red-alk-tryp-001.raw	1	1	P86822.2:P0C8W4.2:AFP87551.1:Q0GY42.1:ABE98265.1:JAG85223.1:ABV64385.1:ABE98262.1:Q0GY45.1:Q0GY43.1:ABE98264.1	Carbamidomethylation	C2:Carbamidomethylation:1000.00
**PEAAPEPAAAAPER(-43.05)R**	23,03	1488,721	15	-2,1	745,3661	38,67	0	1	13178	150211-Fr_I_Helo-red-alk-tryp-001.raw	1	1	JAW07022.1	Arginine oxidation to glutamic semialdehyde	R14:Arginine oxidation to glutamic semialdehyde:0.00
**E(-18.01)TNAKPPA**	23,01	808,4079	8	0,2	405,2113	5,71	0	1	1425	150211-Fr_I_Helo-red-alk-tryp-001.raw	1	1	JAW06987.1:JAW07038.1:JAW06992.1:P84190.1:JAW07002.1	Pyro-glu from E	E1:Pyro-glu from E:1000.00
**PPDPSVFSDPDRDIELAK(+27.99)K**	23	2153,064	19	-0,4	1077,539	46,12	0	1	15855	150211-Fr_I_Helo-red-alk-tryp-001.raw	1	1	AIJ02118.2	Formylation	K18:Formylation:0.00
**EY(sub S)AKYLLSLPR**	22,97	1351,75	11	-2,9	451,5893	13,66	0	1	4287	150211-Fr_I_Helo-red-alk-tryp-001.raw	1	1			
**APEPAPEPAPEPAPEAAPEAAPEPAAAAPER(-42.02)**	22,93	2955,414	31	3,7	986,1488	38,17	0	1	12999	150211-Fr_I_Helo-red-alk-tryp-001.raw	1	1	JAW07022.1	Ornithine from Arginine	R31:Ornithine from Arginine:1000.00
**IDFS(+58.03)GIPEDIIK**	22,92	1403,742	12	-1,2	702,8775	40,03	0	1	13671	150211-Fr_I_Helo-red-alk-tryp-001.raw	3	3	JAW06987.1:JAW07038.1:JAW06992.1:P84190.1	2 3-dihydro-2 2-dimethyl-7-benzofuranol N-methyl carbamate	S4:2 3-dihydro-2 2-dimethyl-7-benzofuranol N-methyl carbamate:1000.00
**DC(+57.02)PEN(+30.01)DGYIMGSGNNK**	22,92	1799,709	16	0,6	900,8624	6,57	0	1	1732	150211-Fr_I_Helo-red-alk-tryp-001.raw	2	2	JAG85213.1	Carbamidomethylation; Hydroxymethyl	C2:Carbamidomethylation:1000.00;N5:Hydroxymethyl:12.36
**Y(sub F)ATDN(sub E)ALTQHMTLTYVK**	22,91	1968,962	17	3,9	985,4919	34,46	0	1	11645	150211-Fr_I_Helo-red-alk-tryp-001.raw	2	2	AIJ02113.2		
**PAPEPAPEAAPEAAPEPAAAAPERR(+453.21)**	22,84	2915,431	25	-0,7	1458,722	36,75	0	1	12474	150211-Fr_I_Helo-red-alk-tryp-001.raw	2	2	JAW07022.1	Puromycin	R25:Puromycin:1000.00
**SC(+57.02)FIRPAGFC(+57.02)DGYC(+57.02)K(+56.06)**	22,75	1892,837	15	-2,2	631,9516	34,38	0	1	11616	150211-Fr_I_Helo-red-alk-tryp-001.raw	1	1	JAW07016.1:JAW06979.1:P68411.1:1913435A:P68410.1:AAB36322.1	Carbamidomethylation; Diethylation	C2:Carbamidomethylation:1000.00;C10:Carbamidomethylation:1000.00;C14:Carbamidomethylation:1000.00;K15:Diethylation:39.78

**AEIDFSGIPEDIIKQIKETNAKPPARF(+123.01)**	22,73	3149,616	27	-2,6	1575,811	39,89	0	1	13617	150211-Fr_I_Helo-red-alk-tryp-001.raw	6	6	JAW06987.1:JAW07038.1:JAW06992.1	glycosylphosphatidylinositol	F27:glycosylphosphatidylinositol:1000.00
**EALAPEPAPEPAPEPAPEAAP**	22,7	2049,989	21	1	684,3377	35,6	0	1	12050	150211-Fr_I_Helo-red-alk-tryp-001.raw	1	1	JAW07022.1:P86821.1		
**WS(sub A)DN(sub E)VLNLDDFETIR**	22,69	1835,869	15	4,5	918,9459	47,09	0	1	16194	150211-Fr_I_Helo-red-alk-tryp-001.raw	2	2	JAW07036.1:JAW06986.1		
**EPAPEPAPEPAPE(+356.19)AAPE**	22,66	2053,967	17	-3,4	1027,987	18,74	0	1	6181	150211-Fr_I_Helo-red-alk-tryp-001.raw	8	8	JAW07022.1:P86821.1:P0C8W6.1	Biotin polyethyleneoxide amine	E13:Biotin polyethyleneoxide amine:15.39
**G(sub K)YFFTLPQASC(+57.02)LR**	22,56	1558,76	13	-0,4	780,3871	38,26	0	1	13035	150211-Fr_I_Helo-red-alk-tryp-001.raw	1	1	JAW07039.1	Carbamidomethylation	C11:Carbamidomethylation:1000.00
**LPSAAN(+203.08)MMQ(+.98)MEWDDELAAIAQAHANQC(+57.02)K**	22,54	3347,457	28	-4,5	670,4956	14,73	0	1	4678	150211-Fr_I_Helo-red-alk-tryp-001.raw	1	1	JAG85161.1	HexNAcylation (N); Deamidation (NQ); Carbamidomethylation	N6:HexNAcylation (N):32.45;Q9:Deamidation (NQ):16.22;C27:Carbamidomethylation:1000.00
**SDVTGGFPVNY(sub S)NNCIYPC(+57.02)YSTQDEIQC(+57.02)EEFC(+57.02)EK**	22,51	3965,617	33	2,1	1322,882	44,83	0	1	15400	150211-Fr_I_Helo-red-alk-tryp-001.raw	2	2	JAW07006.1	Carbamidomethylation	C18:Carbamidomethylation:1000.00;C27:Carbamidomethylation:1000.00;C31:Carbamidomethylation:1000.00
**Q(sub A)EIDFSGIE(sub P)EDIIK**	22,48	1634,804	14	-2,2	818,4074	39,6	0	1	13516	150211-Fr_I_Helo-red-alk-tryp-001.raw	1	1	JAW06987.1:JAW07038.1:JAW06992.1		
**M(sub R)M(sub A)EIDFSGIPEDIIK**	22,44	1736,836	15	-0,8	869,4248	47,67	0	1	16395	150211-Fr_I_Helo-red-alk-tryp-001.raw	1	1	JAW06987.1:JAW07038.1:JAW06992.1		
**IWTSETN(+.98)K(-.98)**	22,43	977,4818	8	0,3	489,7484	72,79	0	1	24572	150211-Fr_I_Helo-red-alk-tryp-001.raw	1	1	AAC25688.1:O77463.1:AAC25689.1	Deamidation (NQ); Amidation	N7:Deamidation (NQ):1000.00;K8:Amidation:1000.00
**LIIQ(sub E)QGDVLAAR**	22,42	1295,756	12	0,6	648,8857	32,26	0	1	10850	150211-Fr_I_Helo-red-alk-tryp-001.raw	7	7	XP_023239442.1		
**PEAAPEPAAAAPE**	22,37	1219,572	13	-0,8	610,7928	13,77	0	1	4328	150211-Fr_I_Helo-red-alk-tryp-001.raw	14	14	JAW07022.1:P86821.1:P0C8W6.1		
**M(sub A)EM(ins)IDFSGIPEDIIK**	22,34	1736,836	15	0,7	869,4261	48,33	0	1	16621	150211-Fr_I_Helo-red-alk-tryp-001.raw	1	1	P84190.1		
**KEALAPEPAPK(+58.01)PAPEPA**	22,32	1769,92	17	0	590,9806	31,14	0	1	10459	150211-Fr_I_Helo-red-alk-tryp-001.raw	1	1	P0C8W6.1	Carboxymethyl (KW X@N-term)	K11:Carboxymethyl (KW X@N-term):88.74
**SSLLFLTPIFVK(+21.98)**	22,31	1385,794	12	1,2	693,9048	50,25	0	1	17276	150211-Fr_I_Helo-red-alk-tryp-001.raw	1	1	JAW07036.1	Sodium adduct	K12:Sodium adduct:1000.00
**VGTFDASGAFMENK**	22,27	1472,661	14	-1,6	737,3364	24,44	0	1	8266	150211-Fr_I_Helo-red-alk-tryp-001.raw	1	1			
**KEALAPE(+37.95)PAPEP**	22,24	1285,587	12	0,1	643,8007	30,75	0	1	10327	150211-Fr_I_Helo-red-alk-tryp-001.raw	1	1	JAW07022.1:P86821.1	Replacement of 2 protons by calcium	E7:Replacement of 2 protons by calcium:35.20
**Q(sub A)EIDFSGIPEDIIG(sub K)QIK**	22,21	1900,978	17	-1,5	634,6657	50,02	0	1	17197	150211-Fr_I_Helo-red-alk-tryp-001.raw	2	2	JAW06987.1:JAW07038.1:JAW06992.1		
**E(+21.98)TNAKPPA**	22,17	848,4004	8	-0,5	425,2072	22,84	0	1	7705	150211-Fr_I_Helo-red-alk-tryp-001.raw	6	6	JAW06987.1:JAW07038.1:JAW06992.1:P84190.1:JAW07002.1	Sodium adduct	E1:Sodium adduct:17.62
**APEPAPEAAPEAAPE(+17.03)PAAAAP**	22,13	1940,948	21	-1,3	971,48	34,36	0	1	11608	150211-Fr_I_Helo-red-alk-tryp-001.raw	1	1	JAW07022.1:P86821.1	Replacement of proton with ammonium ion	E15:Replacement of proton with ammonium ion:0.00

**DVDFTGIADSIIL(sub K)K**	22,1	1505,798	14	0,1	753,9062	50,14	0	1	17237	150211-Fr_I_Helo-red-alk-tryp-001.raw	1	1	JAW07002.1		
**G(sub K)IWTSETNK**	22,03	1034,503	9	0,1	518,259	17,47	0	1	5698	150211-Fr_I_Helo-red-alk-tryp-001.raw	43	43	AAC25688.1:O77463.1:AAC25689.1		
**DNLIPGPPAAFDPDVL**	22,02	1649,83	16	-0,2	825,9221	49,77	0	1	17113	150211-Fr_I_Helo-red-alk-tryp-001.raw	1	1	AIJ02109.2:P86392.3:V9Z9A3.1:AHE40589.1:AHE40588.1:JAG85220.1		
**AN(sub D)T(sub V)DFTGIADSIIKK**	21,91	1592,841	15	4,7	531,9567	36,52	0	1	12385	150211-Fr_I_Helo-red-alk-tryp-001.raw	1	1	JAW07002.1		
**AEIDFSGIPEDIIKP(sub E)**	21,85	1642,845	15	-3,7	822,4269	46,03	0	1	15826	150211-Fr_I_Helo-red-alk-tryp-001.raw	2	2	P84190.1		
**EPAPEPAPE(+356.19)PAPEAAPE**	21,81	2053,967	17	-1,5	1027,989	20,38	0	1	6803	150211-Fr_I_Helo-red-alk-tryp-001.raw	11	11	JAW07022.1:P86821.1:P0C8W6.1	Biotin polyethyleneoxide amine	E9:Biotin polyethyleneoxide amine:0.00
**EPAPK(+14.96)PAPEPAPEPAPEPAPEA**	21,8	2203,032	22	-3,4	1102,52	17,11	0	1	5562	150211-Fr_I_Helo-red-alk-tryp-001.raw	2	2	P0C8W6.1	Alpha-amino adipic acid	K5:Alpha-amino adipic acid:1000.00
**E(+104.03)IDFSGIPEDIIK**	21,79	1578,782	13	4,1	790,4014	38,9	0	1	13262	150211-Fr_I_Helo-red-alk-tryp-001.raw	1	1	JAW06987.1:JAW07038.1:JAW06992.1:P84190.1	Benzoyl	E1:Benzoyl:73.35
**K(+114.04)EGYAMDHEGC(+57.02)K**	21,79	1537,629	12	-1,8	769,8204	5,64	0	1	1401	150211-Fr_I_Helo-red-alk-tryp-001.raw	5	5	JAW07016.1:JAW06979.1:P68411.1:1913435A:P68410.1:AAB36322.1:AAW72453.1:Q5G8B8.1:C0HJM9.1:JAG85185.1:CEF39517.1:P60276.1	Ubiquitin; Carbamidomethylation	K1:Ubiquitin:18.75;C11:Carbamidomethylation:1000.00
**KGSSGYC(+57.02)AW(+19.99)PAC(+57.02)YC(+57.02)YGLPDSVK**	21,73	2545,071	22	0,7	849,3649	37,08	0	1	12595	150211-Fr_I_Helo-red-alk-tryp-001.raw	1	1	AAC25688.1:O77463.1:AAC25689.1	Carbamidomethylation; Tryptophan oxidation to hydroxykynurenin	C7:Carbamidomethylation:1000.00;W9:Tryptophan oxidation to hydroxykynurenin:1000.00;C12:Carbamidomethylation:1000.00;C14:Carbamidomethylation:1000.00
**AEY(ins)T(sub I)DFSGIPEDIIK**	21,72	1696,82	15	0	849,4171	47,12	0	1	16204	150211-Fr_I_Helo-red-alk-tryp-001.raw	1	1	P84190.1		
**AQ(sub E)W(sub I)DFSGIPEDIIK**	21,64	1617,804	14	-3,9	809,9061	40,2	0	1	13731	150211-Fr_I_Helo-red-alk-tryp-001.raw	1	1	JAW06987.1:JAW07038.1:JAW06992.1:P84190.1		
**PEPAAPEPAPEAAPEPVAAA**	21,6	1880,916	20	-2	941,4632	37,68	0	1	12816	150211-Fr_I_Helo-red-alk-tryp-001.raw	1	1	JAG85215.1		
**VDTVAHETAHLIGAPHDEEGPEE(sub Q)TGISGSPGAK**	21,59	3307,559	33	5,6	662,5228	15,72	0	1	5041	150211-Fr_I_Helo-red-alk-tryp-001.raw	1	1	AIJ02110.2		
**PEDIIKQIKETNAKPPAR(-42.02)FDPAAF**	21,58	2653,411	24	-2,9	885,4752	48,84	0	1	16794	150211-Fr_I_Helo-red-alk-tryp-001.raw	1	1	JAW06987.1:JAW07038.1:JAW06992.1	Ornithine from Arginine	R18:Ornithine from Arginine:1000.00
**T(sub V)DQ(sub E)ETFNEVYVC(+57.02)NYSPATIK**	21,55	2378,074	20	3,2	1190,048	37,78	0	1	12852	150211-Fr_I_Helo-red-alk-tryp-001.raw	1	1	JAW07036.1:JAW06986.1	Carbamidomethylation	C12:Carbamidomethylation:1000.00
**APEPAPEAAPE(+14.02)PAAAAP**	21,55	1569,767	17	-2,2	785,8892	33,96	0	1	11465	150211-Fr_I_Helo-red-alk-tryp-001.raw	1	1	P0C8W6.1	Methylation(others)	E11:Methylation(others):16.51
**DVTMTGSK(+28.03)PFTTQ(+.98)K**	21,52	1568,776	14	-4	785,392	30,39	0	1	10200	150211-Fr_I_Helo-red-alk-tryp-001.raw	2	2	JAG85161.1	Dimethylation(KR); Deamidation (NQ)	K8:Dimethylation(KR):5.89;Q13:Deamidation (NQ):1000.00

**WWGN(sub G)EPIWVTAR**	21,5	1513,747	12	-1,6	757,8793	42,62	0	1	14608	150211-Fr_I_Helo-red-alk-tryp-001.raw	1	1	XP_023242894.1		
**AEIDFSGIPE(+21.97)DIIK**	21,47	1567,762	14	-4,5	784,8847	93,47	0	1	31160	150211-Fr_I_Helo-red-alk-tryp-001.raw	1	1	JAW06987.1:JAW07038.1:JAW06992.1:P84190.1	Replacement of 2 protons by magnesium	E10:Replacement of 2 protons by magnesium:0.00
**LAPEPAPEP**	21,43	919,465	9	-0,2	460,7397	30,61	0	1	10276	150211-Fr_I_Helo-red-alk-tryp-001.raw	1	1	JAW07022.1:P86821.1		
**KQ(sub V)NEPSFIGDSE(sub A)ILGHQQVLDPVDLVK**	21,36	3004,551	27	-2,9	752,1427	43,69	0	1	14994	150211-Fr_I_Helo-red-alk-tryp-001.raw	3	3	JAG85213.1		
**IMPIV(sub I)AVADS(sub Q)GYLV(sub I)AK**	21,35	1645,911	16	1,2	549,645	38,09	0	1	12970	150211-Fr_I_Helo-red-alk-tryp-001.raw	2	2	XP_023242894.1		
**APEPAPEAAPEAAPEPAAA**	21,27	1755,831	19	-1,2	586,2837	32,81	0	1	11048	150211-Fr_I_Helo-red-alk-tryp-001.raw	1	1	JAW07022.1:P86821.1		
**A(sub E)V(sub I)DFSGIPEDIIK**	21,25	1402,734	13	1,1	702,3752	39,13	0	1	13343	150211-Fr_I_Helo-red-alk-tryp-001.raw	10	10	JAW06987.1:JAW07038.1:JAW06992.1:P84190.1		
**AEIDFSGIPEDIIK(+162.08)EI**	21,21	1950	16	-1,1	976,0063	49,89	0	1	17154	150211-Fr_I_Helo-red-alk-tryp-001.raw	1	1	P84190.1	O-Pinacolylmethylphosphonylation	K14:O-Pinacolylmethylphosphonylation:24.34
**ADVDFTGIAD(+37.96)S**	21,17	1147,444	11	-3	574,7273	33,17	0	1	11175	150211-Fr_I_Helo-red-alk-tryp-001.raw	1	1	JAW07002.1:P86824.1	Replacement of proton by potassium	D10:Replacement of proton by potassium:7.65
**AEIDFSGIPEDIIK(+42.05)QIK**	21,04	1957,077	17	0	653,3663	53,84	0	1	18458	150211-Fr_I_Helo-red-alk-tryp-001.raw	1	1		Trimethylation	K14:Trimethylation:6.63
**EALAPEPAPK(+164.06)PAPEPAPEPA**	20,98	2142,065	20	-4,5	1072,035	18,75	0	1	6186	150211-Fr_I_Helo-red-alk-tryp-001.raw	1	1	P0C8W6.1	O-Diisopropylphosphorylation	K10:O-Diisopropylphosphorylation:21.89
**TEPSFIKDNLIPGPPAAFDPDVLISAM(+15.99)SK(+42.02)**	20,95	3127,59	29	0,9	626,5258	50,74	0	1	17438	150211-Fr_I_Helo-red-alk-tryp-001.raw	1	1	AIJ02109.2:P86392.3:V9Z9A3.1:AHE40589.1:AHE40588.1	Oxidation (M); Guanidination	M27:Oxidation (M):1000.00;K29:Guanidination:6.58
**GSSGYC(+57.02)AWPAC(+57.02)YC(+57.02)YGLPDSVP(-27.99)**	20,86	2337,949	21	0,3	1169,982	43,97	0	1	15092	150211-Fr_I_Helo-red-alk-tryp-001.raw	1	1		Carbamidomethylation; Pyrrolidone from Proline	C6:Carbamidomethylation:1000.00;C11:Carbamidomethylation:1000.00;C13:Carbamidomethylation:1000.00;P21:Pyrrolidone from Proline:0.00
**KTQFGC(+57.02)PAYEGY(+44.99)C(+57.02)NNHC(+57.02)QDIER**	20,83	2791,117	22	2,9	931,3823	31,53	0	1	10592	150211-Fr_I_Helo-red-alk-tryp-001.raw	1	1	Q5G8A6.1:JAG85216.1:AAW72464.1:AAW72465.1	Carbamidomethylation; Oxidation to nitro	C6:Carbamidomethylation:1000.00;Y12:Oxidation to nitro:28.96;C13:Carbamidomethylation:1000.00;C17:Carbamidomethylation:1000.00
**AEIDFSGIPEDIIKQIK(+54.01)ETNA**	20,78	2384,211	21	3,5	1193,117	39,68	0	1	13543	150211-Fr_I_Helo-red-alk-tryp-001.raw	1	1	JAW06987.1:JAW07038.1:JAW06992.1	MDA adduct +54	K17:MDA adduct +54:16.66
**WWN(sub G)GEPIWVTAR**	20,73	1513,747	12	-1,3	757,8796	43,58	0	1	14954	150211-Fr_I_Helo-red-alk-tryp-001.raw	4	4	XP_023242894.1		
**EALAPE(+17.03)PAPEP**	20,72	1136,571	11	0,6	569,2933	31,19	0	1	10475	150211-Fr_I_Helo-red-alk-tryp-001.raw	1	1	JAW07022.1:P86821.1	Replacement of proton with ammonium ion	E6:Replacement of proton with ammonium ion:0.00
**E(sub A)EIY(sub D)FSGIPEDIIK**	20,54	1651,835	14	1,2	826,9255	46,23	0	1	15895	150211-Fr_I_Helo-red-alk-tryp-001.raw	2	2	JAW06987.1:JAW07038.1:JAW06992.1:P84190.1		
**R(-42.02)AEIDFSGIPEDIIK**	20,53	1659,872	15	3,4	830,946	49,47	0	1	17011	150211-Fr_I_Helo-red-alk-tryp-001.raw	1	1	JAW06987.1:JAW07038.1:JAW06992.1	Ornithine from Arginine	R1:Ornithine from Arginine:1000.00

**APEPAPEAAPEAAPEPAAAAP(+13.98)ERR**	20,51	2379,145	24	-0,6	1190,579	40,44	0	1	13818	150211-Fr_I_Helo-red-alk-tryp-001.raw	1	1	JAW07022.1	Proline oxidation to pyroglutamic acid	P21:Proline oxidation to pyroglutamic acid:9.30
**DC(+57.02)PES(+58.03)DGYIMGSGNNK**	20,4	1800,717	16	-3,4	901,3627	5,81	0	1	1462	150211-Fr_I_Helo-red-alk-tryp-001.raw	1	1	AIJ02110.2	Carbamidomethylation; 2 3-dihydro-2 2-dimethyl-7-benzofuranol N-methyl carbamate	C2:Carbamidomethylation:1000.00;S5:2 3-dihydro-2 2-dimethyl-7-benzofuranol N-methyl carbamate:15.08
**Y(sub F)V(sub I)NGGTDNSGIIITLLPGS(sub T)DS(sub P)SVTK**	20,37	2507,275	25	2,8	1254,648	48,35	0	1	16626	150211-Fr_I_Helo-red-alk-tryp-001.raw	1	1	XP_023239442.1		
**SGAYFWP(+29.04)ASDIPIK**	20,28	1579,816	14	-3,8	790,9123	45,62	0	1	15681	150211-Fr_I_Helo-red-alk-tryp-001.raw	1	1	XP_023242894.1	Dimethylation of proline residue	P7:Dimethylation of proline residue:38.32
**S(+58.03)C(+57.02)PYDDGYIMGSTIEPNMLK**	20,25	2348,025	20	-1,7	783,6809	34,14	0	1	11529	150211-Fr_I_Helo-red-alk-tryp-001.raw	1	1	JAG85190.1	2 3-dihydro-2 2-dimethyl-7-benzofuranol N-methyl carbamate; Carbamidomethylation	S1:2 3-dihydro-2 2-dimethyl-7-benzofuranol N-methyl carbamate:5.16;C2:Carbamidomethylation:1000.00
**EPAPEPAPEPAPE(+17.03)AAPEAAPEP**	20,21	2180,027	22	2,4	727,6847	34,38	0	1	11614	150211-Fr_I_Helo-red-alk-tryp-001.raw	1	1	JAW07022.1:P86821.1	Replacement of proton with ammonium ion	E13:Replacement of proton with ammonium ion:0.00
**EPAPEPAPEAAPEP(-27.99)AAAAPEAAPE**	20,19	2251,064	24	0,3	751,3622	37,03	0	1	12579	150211-Fr_I_Helo-red-alk-tryp-001.raw	4	4	P0C8W6.1	Pyrrolidone from Proline	P14:Pyrrolidone from Proline:0.00
**EALAPE(+17.03)PAPEPAP**	20,16	1304,661	13	0,1	653,3379	19,38	2,30E+08	1	6427	150211-Fr_I_Helo-red-alk-tryp-001.raw	11	11	JAW07022.1:P86821.1	Replacement of proton with ammonium ion	E6:Replacement of proton with ammonium ion:4.75
**AEIDFSGIPE(+17.03)D**	19,97	1208,556	11	0,2	605,2854	33,4	0	1	11257	150211-Fr_I_Helo-red-alk-tryp-001.raw	5	5	JAW06987.1:JAW07038.1:JAW06992.1:P84190.1	Replacement of proton with ammonium ion	E10:Replacement of proton with ammonium ion:0.00
**Q(sub A)EQ(sub I)DFSGIPEDIIK**	19,96	1617,789	14	1,9	809,9031	41,22	0	1	14104	150211-Fr_I_Helo-red-alk-tryp-001.raw	1	1	JAW06987.1:JAW07038.1:JAW06992.1:P84190.1		
**DQ(sub A)PPLN(sub D)LPQ(sub D)GVGFK**	19,89	1508,799	14	-0,1	755,4066	36,61	0	1	12419	150211-Fr_I_Helo-red-alk-tryp-001.raw	2	2	XP_023239442.1		
**AEIDFSGIPEDIIKQIKETNAKPPAR(+39.99)FDP**	19,89	3278,682	29	-4	1093,897	41,82	0	1	14320	150211-Fr_I_Helo-red-alk-tryp-001.raw	1	1	JAW06987.1:JAW07038.1:JAW06992.1	Glyoxal-derived hydroimiadazolone	R26:Glyoxal-derived hydroimiadazolone:1000.00
**AEID(+43.04)FSGIPEDIIK**	19,81	1588,835	14	4,4	795,4282	50,54	0	1	17373	150211-Fr_I_Helo-red-alk-tryp-001.raw	1	1	JAW06987.1:JAW07038.1:JAW06992.1:P84190.1	Carboxyl modification with ethanolamine	D4:Carboxyl modification with ethanolamine:0.00
**LLGTEPIIRI**	19,79	1123,696	10	-0,1	562,8554	33,79	0	1	11400	150211-Fr_I_Helo-red-alk-tryp-001.raw	1	1	AIJ02118.2		
**AEIDFSGIPEDIIK(+27.99)QI**	19,77	1814,93	16	2,1	908,4742	52,47	0	1	18011	150211-Fr_I_Helo-red-alk-tryp-001.raw	1	1	JAW06987.1:JAW07038.1:JAW06992.1	Formylation	K14:Formylation:21.35
**P(+71.04)APEPAPEAAPEAAPEPAAAAPERR**	19,7	2533,256	25	-4,7	1267,629	36,8	0	1	12492	150211-Fr_I_Helo-red-alk-tryp-001.raw	1	1	JAW07022.1	Propionamide (K X@N-term)	P1:Propionamide (K X@N-term):1000.00
**W(sub A)Q(sub E)F(sub I)DFSGIPEDIIK**	19,68	1693,835	14	-3,8	847,9216	44,89	0	1	15421	150211-Fr_I_Helo-red-alk-tryp-001.raw	4	4	JAW06987.1:JAW07038.1:JAW06992.1:P84190.1		
**N(sub V)SESDKQTILKT(sub V)HNELR**	19,64	2012,029	17	3	671,6855	6,85	0	1	1832	150211-Fr_I_Helo-red-alk-tryp-001.raw	2	2			
**EN(sub I)DFSGIPEDIIK**	19,62	1475,714	13	3,2	738,8668	47,22	0	1	16241	150211-Fr_I_Helo-red-alk-tryp-001.raw	1	1	JAW06987.1:JAW07038.1:JAW06992.1:P84190.1		
**Y(sub S)DVTGGFPVNSNNCIYPC(+57.0**	19,59	3965,617	33	2,5	1322,883	45,28	0	1	15559	150211-Fr_I_Helo-red-alk-tryp-001.raw	1	1	JAW07006.1	Carbamidomethylation	C18:Carbamidomethylation:1000.00;C27:Carbamidomethylat

**2)YSTQDEIQC(+57.02)EEFC(+57.02)EK**															ion:1000.00;C31:Carbamidomethylation:1000.00
**ESDPIHVQLLK**	19,57	1277,698	11	-1,9	639,855	40,72	0	1	13919	150211-Fr_I_Helo-red-alk-tryp-001.raw	2	2			
**EPAPK(+154.10)PAPEPAPEPAPEPAPEAAPEPAAAAPE**	19,55	3246,597	32	-1	1083,205	48,28	0	1	16605	150211-Fr_I_Helo-red-alk-tryp-001.raw	1	1	P0C8W6.1	4-Oxononenal (ONE)	K5:4-Oxononenal (ONE):1000.00
**AIITVVDNGIMIGC(+57.02)GIFDVAS(+58.03)PSVK**	19,54	2633,368	25	-3,1	659,3473	49,13	0	1	16896	150211-Fr_I_Helo-red-alk-tryp-001.raw	1	1	JAW07019.1:JAW06978.1	Carbamidomethylation; 2 3-dihydro-2 2-dimethyl-7-benzofuranol N-methyl carbamate	C14:Carbamidomethylation:1000.00;S21:2 3-dihydro-2 2-dimethyl-7-benzofuranol N-methyl carbamate:14.04
**SN(sub L)FVGEDVFNF(sub I)ELIAK**	19,53	1827,904	16	3,7	914,9628	50,79	0	1	17454	150211-Fr_I_Helo-red-alk-tryp-001.raw	1	1	JAW06984.1		
**TEPSFIKDNLIPGPPAAFDPDVLIS(+58.03)AM**	19,46	2912,476	27	-1,5	971,8311	53,14	0	1	18232	150211-Fr_I_Helo-red-alk-tryp-001.raw	1	1	AIJ02109.2:P86392.3:V9Z9A3.1:AHE40589.1:AHE40588.1	2 3-dihydro-2 2-dimethyl-7-benzofuranol N-methyl carbamate	S25:2 3-dihydro-2 2-dimethyl-7-benzofuranol N-methyl carbamate:14.35
**EALAPE(+17.03)PAPEPAPEPAPEAAP**	19,46	2067,016	21	-0,5	1034,515	33,83	4,99E+08	1	11415	150211-Fr_I_Helo-red-alk-tryp-001.raw	1	1	JAW07022.1:P86821.1	Replacement of proton with ammonium ion	E6:Replacement of proton with ammonium ion:0.00
**SDVTGGFPVNF(sub S)NNCIYPC(+57.02)YSTQDEIQC(+57.02)EEFC(+57.02)EK**	19,42	3949,622	33	-3	988,4097	44,98	0	1	15453	150211-Fr_I_Helo-red-alk-tryp-001.raw	1	1	JAW07006.1	Carbamidomethylation	C18:Carbamidomethylation:1000.00;C27:Carbamidomethylation:1000.00;C31:Carbamidomethylation:1000.00
**ADT(sub V)N(sub D)FTGIADSIIKK**	19,38	1592,841	15	5,2	531,957	36,72	0	1	12463	150211-Fr_I_Helo-red-alk-tryp-001.raw	1	1	JAW07002.1		
**ES(+58.03)LIPGEEVFDFGHVVGNL**	19,34	2115,04	19	4,2	1058,532	54,96	0	1	18823	150211-Fr_I_Helo-red-alk-tryp-001.raw	1	1	AIJ02112.2	2 3-dihydro-2 2-dimethyl-7-benzofuranol N-methyl carbamate	S2:2 3-dihydro-2 2-dimethyl-7-benzofuranol N-methyl carbamate:1000.00
**EIKET(+79.96)NAKPPA**	19,33	1276,597	11	-2,9	639,3039	9,32	0	1	2735	150211-Fr_I_Helo-red-alk-tryp-001.raw	2	2	P84190.1	Sulfation	T5:Sulfation:1000.00
**T(sub V)TQ(sub E)SDKQTILETHNQLR**	19,29	2012,029	17	2,3	671,6851	10,1	0	1	3016	150211-Fr_I_Helo-red-alk-tryp-001.raw	1	1	JAW07031.1:JAW06995.1		
**AEIDFSGIPED(+17.03)**	19,23	1208,556	11	-0,1	605,2852	31,64	0	1	10631	150211-Fr_I_Helo-red-alk-tryp-001.raw	2	2	JAW06987.1:JAW07038.1:JAW06992.1:P84190.1	Replacement of proton with ammonium ion	D11:Replacement of proton with ammonium ion:0.00
**AEIDFSGIPE**	19,14	1076,503	10	-0,6	539,2582	32,09	0	1	10788	150211-Fr_I_Helo-red-alk-tryp-001.raw	2	2	JAW06987.1:JAW07038.1:JAW06992.1:P84190.1		
**T(+380.15)SESEYAC(+57.02)PAIDK**	19,07	1849,782	13	3,2	925,901	6,6	0	1	1744	150211-Fr_I_Helo-red-alk-tryp-001.raw	1	1	Q0GY42.1:ABE98265.1:JAG85223.1	Nucleophilic addition to cytopiloyne+H2O; Carbamidomethylation	T1:Nucleophilic addition to cytopiloyne+H2O:0.00;C8:Carbamidomethylation:1000.00
**PGEEVFDFGHVVGNLNVLNC(+57.02)KYK(+59.05)D**	18,92	2808,367	24	-3,6	937,1261	52,54	0	1	18034	150211-Fr_I_Helo-red-alk-tryp-001.raw	2	2	AIJ02112.2	Carbamidomethylation; 5-hydroxy-N6 N6 N6-trimethyl	C20:Carbamidomethylation:1000.00;K23:5-hydroxy-N6 N6 N6-trimethyl:7.32
**PEAAPEAAPEPAAAAPE(+43.04)**	18,9	1630,784	17	-0,3	816,3989	13,98	0	1	4402	150211-Fr_I_Helo-red-alk-tryp-001.raw	21	21	JAW07022.1:P86821.1	Carboxyl modification with ethanolamine	E17:Carboxyl modification with ethanolamine:26.08
**C(+43.01)NALLYRKVR**	18,89	1277,703	10	0,8	639,8591	42,8	0	1	14675	150211-Fr_I_Helo-red-alk-tryp-001.raw	1	1	JAW07023.1	Carbamylation	C1:Carbamylation:44.44
**EALAPE(+17.03)PAPEPAPEPAP**	18,87	1698,846	17	1	850,4313	14,94	0	1	4754	150211-Fr_I_Helo-red-alk-tryp-001.raw	8	8	JAW07022.1:P86821.1	Replacement of proton with ammonium ion	E6:Replacement of proton with ammonium ion:0.00

**K(+42.02)EGYAM(+15.99)DHEGC(+57.02)K**	18,78	1481,603	12	3,6	741,8113	7,46	0	1	2057	150211-Fr_I_Helo-red-alk-tryp-001.raw	5	5	P68411.1:1913435A:P68410.1:AAB36322.1:AAW72453.1:Q5G8B8.1:P60276.1	Guanidination; Oxidation (M); Carbamidomethylation	K1:Guanidination:26.12;M6:Oxidation (M):1000.00;C11:Carbamidomethylation:1000.00
**SLNHTMYSK**	18,67	1079,507	9	0	540,7607	18,24	0	1	5992	150211-Fr_I_Helo-red-alk-tryp-001.raw	1	1			
**AHEAAHLLAVPHDGDDFK(+127.06)VSDIPNSPGAK**	18,58	3134,542	29	-0,2	523,4308	36,01	0	1	12200	150211-Fr_I_Helo-red-alk-tryp-001.raw	1	1	AIJ02111.3	N-Succinimidyl-2-morpholine acetate	K18:N-Succinimidyl-2-morpholine acetate:2.27
**AEN(sub I)DFSGIPEH(sub D)IIK**	18,58	1568,783	14	-2,2	523,934	38,5	0	1	13120	150211-Fr_I_Helo-red-alk-tryp-001.raw	1	1	JAW06987.1:JAW07038.1:JAW06992.1		
**APEPAAAAPER(+212.01)RRR**	18,49	1758,853	14	-4,4	880,4297	15,14	0	1	4830	150211-Fr_I_Helo-red-alk-tryp-001.raw	2	2	JAW07022.1	phosphate-ribosylation	R11:phosphate-ribosylation:0.00
**EPAPEPAPEAAPE(+21.98)AAPE**	18,44	1693,745	17	-1,5	847,8783	35,08	0	1	11865	150211-Fr_I_Helo-red-alk-tryp-001.raw	1	1	JAW07022.1:P86821.1	Sodium adduct	E13:Sodium adduct:0.00
**AEIDFSGIPE(+37.95)DII**	18,42	1455,645	13	1,2	486,2227	47,02	0	1	16170	150211-Fr_I_Helo-red-alk-tryp-001.raw	1	1	JAW06987.1:JAW07038.1:JAW06992.1:P84190.1	Replacement of 2 protons by calcium	E10:Replacement of 2 protons by calcium:11.06
**SNSW(sub A)LTNYVTVMFTGVQNLMDTLELGIGVR**	18,38	3357,674	30	0,1	840,4258	48,78	0	1	16774	150211-Fr_I_Helo-red-alk-tryp-001.raw	1	1	AIJ02109.2:P86392.3:V9Z9A3.1:AHE40589.1:AHE40588.1:AHE40596.1:V9ZAY0.1		
**LAPEPAPEPAPEPAPE(+17.03)A**	18,31	1698,846	17	-0,9	850,4296	16,26	0	1	5242	150211-Fr_I_Helo-red-alk-tryp-001.raw	3	3	JAW07022.1:P86821.1	Replacement of proton with ammonium ion	E16:Replacement of proton with ammonium ion:0.00
**PAPEAAPEAAPEPAAAAPE(+6.01)R**	18,25	1917,941	20	-2,7	959,975	13,7	0	1	4302	150211-Fr_I_Helo-red-alk-tryp-001.raw	1	1	JAW07022.1	Replacement of proton by lithium	E19:Replacement of proton by lithium:7.21
**APEPAPE(+21.98)AAPEA**	18,22	1170,517	12	-2,8	586,264	30,18	0	1	10132	150211-Fr_I_Helo-red-alk-tryp-001.raw	1	1	JAW07022.1:P86821.1	Sodium adduct	E7:Sodium adduct:19.56
**EPAPEAAPEPVAAAAPER(-.98)**	18,19	1770,89	18	-2	886,4505	23,43	0	1	7914	150211-Fr_I_Helo-red-alk-tryp-001.raw	9	9	JAG85215.1	Amidation	R18:Amidation:1000.00
**I(+41.03)RPSGFC(+57.02)DGYC(+57.02)K**	18,15	1499,665	12	-0,9	500,8951	30,66	0	1	10294	150211-Fr_I_Helo-red-alk-tryp-001.raw	1	1	C0HJM9.1:JAG85185.1:CEF39517.1:P60276.1	Amidination of lysines or N-terminal amines with methyl acetimidate; Carbamidomethylation	I1:Amidination of lysines or N-terminal amines with methyl acetimidate:44.44;C7:Carbamidomethylation:1000.00;C11:Carbamidomethylation:1000.00
**KTQFGC(+57.02)PAYEGY(+44.99)C(+57.02)NNHC(+57.02)QDIERK**	18,03	2919,212	23	1,7	730,8116	31,09	0	1	10443	150211-Fr_I_Helo-red-alk-tryp-001.raw	2	2	Q5G8A6.1:JAG85216.1:AAW72464.1:AAW72465.1	Carbamidomethylation; Oxidation to nitro	C6:Carbamidomethylation:1000.00;Y12:Oxidation to nitro:9.42;C13:Carbamidomethylation:1000.00;C17:Carbamidomethylation:1000.00
**EALAPEPAPK(+362.14)PAPEPAPEPAPEPA**	18	2734,326	24	-0,2	912,4492	37,98	0	1	12929	150211-Fr_I_Helo-red-alk-tryp-001.raw	1	1	P0C8W6.1	Nucleophilic addtion to cytopiloyne	K10:Nucleophilic addtion to cytopiloyne:0.00
**N(sub S)VH(sub Y)DIEANPILLK**	17,98	1474,814	13	-3,2	738,4121	36,36	0	1	12327	150211-Fr_I_Helo-red-alk-tryp-001.raw	1	1	AIJ02118.2		
**NPC(+57.02)IIIDH(sub Y)LC(+57.02)VTETTFA(sub T)ER**	17,96	2288,093	19	-2,5	763,7031	51,49	0	1	17689	150211-Fr_I_Helo-red-alk-tryp-001.raw	1	1	JAG85213.1	Carbamidomethylation	C3:Carbamidomethylation:1000.00;C10:Carbamidomethylation:1000.00
**APEAAPEAAPEPAAAAPER(-42.02)**	17,96	1772,858	19	-1,1	887,4353	14,44	0	1	4572	150211-Fr_I_Helo-red-alk-tryp-001.raw	2	2	JAW07022.1	Ornithine from Arginine	R19:Ornithine from Arginine:1000.00

**TQFGC(+57.02)PAYEGY(+44.99)C(+57.02)NNHC(+57.02)QDIERK**	17,91	2791,117	22	1,2	931,3808	14,42	0	1	4565	150211-Fr_I_Helo-red-alk-tryp-001.raw	1	1	Q5G8A6.1:JAG85216.1:AAW72464.1:AAW72465.1	Carbamidomethylation; Oxidation to nitro	C5:Carbamidomethylation:1000.00;Y11:Oxidation to nitro:4.05;C12:Carbamidomethylation:1000.00;C16:Carbamidomethylation:1000.00
**G(sub D)FSGIPEDIIK**	17,9	1174,623	11	-0,7	588,3185	34,89	0	1	11799	150211-Fr_I_Helo-red-alk-tryp-001.raw	2	2	JAW06987.1:JAW07038.1:JAW06992.1:P84190.1		
**AN(sub D)T(sub V)DFTGIADSIIKR(sub K)**	17,9	1620,847	15	4,5	541,2921	36,71	0	1	12459	150211-Fr_I_Helo-red-alk-tryp-001.raw	1	1	JAW07002.1		
**ADVDFTGIADS(+162.05)IIK**	17,88	1625,804	14	-1,4	813,9078	46,8	0	1	16091	150211-Fr_I_Helo-red-alk-tryp-001.raw	4	4	JAW07002.1:P86824.1	Hexose (NSY)	S11:Hexose (NSY):1000.00
**AEIDFSGIPEDIIK(+117.02)EIK**	17,86	2033,039	17	4,7	1017,532	48,56	0	1	16697	150211-Fr_I_Helo-red-alk-tryp-001.raw	1	1	P84190.1	N-Homocysteine thiolactone	K14:N-Homocysteine thiolactone:7.77
**IDFSGIPEDII**	17,82	1217,618	11	-0,7	609,8158	45,87	0	1	15768	150211-Fr_I_Helo-red-alk-tryp-001.raw	1	1	JAW06987.1:JAW07038.1:JAW06992.1:P84190.1		
**D(sub K)ITCFLTAAGYC(+57.02)NTEC(+57.02)TLKK**	17,78	2308,054	20	5,2	770,3625	34,24	0	1	11564	150211-Fr_I_Helo-red-alk-tryp-001.raw	1	1	AAC25688.1:O77463.1:AAC25689.1	Carbamidomethylation	C12:Carbamidomethylation:1000.00;C16:Carbamidomethylation:1000.00
**LAPEPAPEPAPE(+17.03)PAPEA**	17,77	1698,846	17	0	850,4304	16,68	0	1	5398	150211-Fr_I_Helo-red-alk-tryp-001.raw	7	7	JAW07022.1:P86821.1	Replacement of proton with ammonium ion	E12:Replacement of proton with ammonium ion:16.65
**AR(sub E)M(sub I)DFSGIPEDIIK**	17,72	1590,808	14	3,7	796,4139	47	0	1	16162	150211-Fr_I_Helo-red-alk-tryp-001.raw	1	1	JAW06987.1:JAW07038.1:JAW06992.1		
**KW(sub G)M(sub S)SGYCAWPAC(+57.02)YC(+57.02)YGLPDSVK**	17,64	2641,126	22	1	1321,572	37,71	0	1	12825	150211-Fr_I_Helo-red-alk-tryp-001.raw	1	1	AAC25688.1:O77463.1:AAC25689.1	Carbamidomethylation	C12:Carbamidomethylation:1000.00;C14:Carbamidomethylation:1000.00
**Q(sub A)EIDFSS(sub G)V(sub I)PEDIIK**	17,63	1618,809	14	-2	810,4102	39,38	0	1	13435	150211-Fr_I_Helo-red-alk-tryp-001.raw	1	1	JAW06987.1:JAW07038.1:JAW06992.1		
**E(+108.00)NVVPENC(+57.02)PK**	17,6	1292,547	10	1	647,2815	5,59	0	1	1385	150211-Fr_I_Helo-red-alk-tryp-001.raw	2	2	JAW07031.1:JAW06995.1	O-Ethylphosphorylation; Carbamidomethylation	E1:O-Ethylphosphorylation:61.14;C8:Carbamidomethylation:1000.00
**FSC(+57.02)FIRPSGFC(+57.02)DGYC(+57.02)K(+42.02)**	17,59	2041,86	16	3,3	681,6294	33,1	0	1	11150	150211-Fr_I_Helo-red-alk-tryp-001.raw	1	1	C0HJM9.1:JAG85185.1:CEF39517.1:P60276.1	Carbamidomethylation; Guanidination	C3:Carbamidomethylation:1000.00;C11:Carbamidomethylation:1000.00;C15:Carbamidomethylation:1000.00;K16:Guanidination:1000.00
**DSWTE(sub Q)LYVC(+57.02)NYGPAGNLDDSELYK**	17,57	2808,222	24	5,6	937,0866	47,11	0	1	16202	150211-Fr_I_Helo-red-alk-tryp-001.raw	1	1	JAW06991.1:JAW07032.1	Carbamidomethylation	C9:Carbamidomethylation:1000.00
**FGPNVAGETAAS(+58.03)EVNFHFSAPHLIFPEGMKETR**	17,56	3644,76	33	-1,4	608,4664	45,98	0	1	15806	150211-Fr_I_Helo-red-alk-tryp-001.raw	1	1	JAW07031.1:JAW06995.1	2 3-dihydro-2 2-dimethyl-7-benzofuranol N-methyl carbamate	S12:2 3-dihydro-2 2-dimethyl-7-benzofuranol N-methyl carbamate:0.00
**NDYLLYCDFSDKDPK**	17,51	1834,808	15	-0,7	612,6096	20,36	0	1	6797	150211-Fr_I_Helo-red-alk-tryp-001.raw	2	2	JAG85161.1		
**TDQALTEYVTQ(+.98)MFTGVQ(+.98)NMYDTMNLEIKI**	17,49	3398,561	29	-3,4	1133,857	49,39	0	1	16984	150211-Fr_I_Helo-red-alk-tryp-001.raw	1	1	JAW07042.1	Deamidation (NQ)	Q11:Deamidation (NQ):1.34;Q17:Deamidation (NQ):7.65

**AEIDFSGIPEDIIK(+58.01)QI**	17,44	1844,941	16	1,3	923,4788	51,78	1,90E+08	1	17785	150211-Fr_I_Helo-red-alk-tryp-001.raw	1	1	JAW06987.1:JAW07038.1:JAW06992.1	Carboxymethyl (KW X@N-term)	K14:Carboxymethyl (KW X@N-term):32.13
**EPAPEPAPEAAPEP**	17,4	1400,646	14	1	701,3309	31,57	0	1	10607	150211-Fr_I_Helo-red-alk-tryp-001.raw	1	1	P0C8W6.1		
**SC(+57.02)PYDDGYIMGSTIEPNM(+15.99)LK(+42.02)**	17,36	2348,012	20	1,8	1175,016	20,57	0	1	6874	150211-Fr_I_Helo-red-alk-tryp-001.raw	1	1	JAG85190.1	Carbamidomethylation; Oxidation (M); Guanidination	C2:Carbamidomethylation:1000.00;M18:Oxidation (M):3.68;K20:Guanidination:1000.00
**APEAAPEPAAAAPERRR(+282.05)R**	17,35	2197,066	18	-1,7	1099,539	34,09	0	1	11512	150211-Fr_I_Helo-red-alk-tryp-001.raw	1	1	JAW07022.1	Bis(hydroxphenylglyoxal) arginine	R17:Bis(hydroxphenylglyoxal) arginine:0.00
**EPAPEPAPEPAPEAAPE(+17.03)AAPEP**	17,32	2180,027	22	2,1	727,6845	34,16	0	1	11535	150211-Fr_I_Helo-red-alk-tryp-001.raw	1	1	JAW07022.1:P86821.1	Replacement of proton with ammonium ion	E17:Replacement of proton with ammonium ion:6.65
**SGIPEDIIKE(-.98)**	17,32	1098,592	10	0,9	550,3038	31,07	0	1	10434	150211-Fr_I_Helo-red-alk-tryp-001.raw	1	1	P84190.1	Amidation	E10:Amidation:1000.00
**APEAAPEPAA**	17,26	922,4396	10	0	462,2271	5,83	0	1	1469	150211-Fr_I_Helo-red-alk-tryp-001.raw	2	2	JAW07022.1:P86821.1:P0C8W6.1		
**AEIDFS(+58.03)GIPEDIIKQ**	17,16	1731,881	15	-2,4	866,9454	41,56	0	1	14227	150211-Fr_I_Helo-red-alk-tryp-001.raw	2	2	JAW06987.1:JAW07038.1:JAW06992.1	2 3-dihydro-2 2-dimethyl-7-benzofuranol N-methyl carbamate	S6:2 3-dihydro-2 2-dimethyl-7-benzofuranol N-methyl carbamate:1000.00
**P(+58.01)APEPAPEAAPEAAPEPAAAAPERRR**	17,15	2676,325	26	-2,8	893,1133	37,26	0	1	12661	150211-Fr_I_Helo-red-alk-tryp-001.raw	1	1	JAW07022.1	Carboxymethyl (KW X@N-term)	P1:Carboxymethyl (KW X@N-term):1000.00
**EPQPSASEAFSK**	17,14	1276,594	12	-0,7	639,3036	6,36	0	1	1658	150211-Fr_I_Helo-red-alk-tryp-001.raw	4	4			
**D(-18.01)GEC(+57.02)HGFK**	17,1	930,3654	8	1,2	466,1905	11,98	0	1	3688	150211-Fr_I_Helo-red-alk-tryp-001.raw	2	2	P0C2F3.1:P69940.2:Q5G8A6.1:JAG85216.1:AAW72464.1:AAW72465.1	Dehydration; Carbamidomethylation	D1:Dehydration:1000.00;C4:Carbamidomethylation:1000.00
**K(+14.96)EGYLMDHEGC(+57.02)K**	17,08	1480,596	12	2,5	741,3073	5,64	3,49E+06	1	1404	150211-Fr_I_Helo-red-alk-tryp-001.raw	1	1	AAB29128.1:P56612.1:AIA81145.1:CAA46982.1:P15226.2:P0DMM8.1:1NPI:1B7D:AAB36321.1:AAB36318.1:P56611.1:JAG85191.1:JAG85210.1	Alpha-amino adipic acid; Carbamidomethylation	K1:Alpha-amino adipic acid:45.45;C11:Carbamidomethylation:1000.00
**PAC(+57.02)YC(+57.02)YGLPDSVKIWTSE(+37.95)TNK**	17,07	2526,087	21	-0,8	1264,05	37,16	0	1	12625	150211-Fr_I_Helo-red-alk-tryp-001.raw	2	2	AAC25688.1:O77463.1:AAC25689.1	Carbamidomethylation; Replacement of 2 protons by calcium	C3:Carbamidomethylation:1000.00;C5:Carbamidomethylation:1000.00;E18:Replacement of 2 protons by calcium:5.77
**LVML(sub A)DETDYNER**	17,03	1496,682	12	-0,5	749,3477	6,78	0	1	1809	150211-Fr_I_Helo-red-alk-tryp-001.raw	2	2	AIJ02111.3		
**KGSSGYC(+57.02)W(sub A)WPAC(+57.02)YC(+57.02)YGLPDSVK**	16,99	2640,124	22	-0,4	881,0481	37,54	0	1	12765	150211-Fr_I_Helo-red-alk-tryp-001.raw	1	1	AAC25688.1:O77463.1:AAC25689.1	Carbamidomethylation	C7:Carbamidomethylation:1000.00;C12:Carbamidomethylation:1000.00;C14:Carbamidomethylation:1000.00
**APEPAPEAAPEA**	16,97	1148,535	12	0,3	575,2749	6,01	0	1	1532	150211-Fr_I_Helo-red-alk-tryp-001.raw	9	9	JAW07022.1:P86821.1		
**EPAPEPAPEAAP(+29.04)EPAAAAPE**	16,95	1939,929	20	-1,4	970,9703	35,63	0	1	12061	150211-Fr_I_Helo-red-alk-tryp-001.raw	1	1	P0C8W6.1	Dimethylation of proline residue	P12:Dimethylation of proline residue:0.00

**ITC(+57.02)FLTAAGYC(+57.02)NTE(+43.99)C(+57.02)TLK(+14.02)**	16,88	2179,959	18	2,7	727,6622	35,1	0	1	11871	150211-Fr_I_Helo-red-alk-tryp-001.raw	1	1	AAC25688.1:O77463.1:AAC25689.1	Carbamidomethylation; Carboxylation (E); Methylation(KR)	C3:Carbamidomethylation:1000.00;C11:Carbamidomethylation:1000.00;E14:Carboxylation (E):1000.00;C15:Carbamidomethylation:1000.00;K18:Methylation(KR):1000.00
**GTIAHWIC(+57.02)LVE(+21.98)HESSYNTAAK**	16,86	2408,119	21	-2,7	1205,064	44,83	0	1	15401	150211-Fr_I_Helo-red-alk-tryp-001.raw	1	1	XP_023218922.1	Carbamidomethylation; Sodium adduct	C8:Carbamidomethylation:1000.00;E11:Sodium adduct:0.00
**N(sub L)LGV(sub I)QAFKIGTEPSFIK**	16,85	1848,015	17	2,7	925,017	46,71	0	1	16062	150211-Fr_I_Helo-red-alk-tryp-001.raw	1	1			
**IFWADSK**	16,81	865,4333	7	-4,7	433,7219	21,44	0	1	7196	150211-Fr_I_Helo-red-alk-tryp-001.raw	2	2			
**KGSSGYCE(sub A)WPAC(+57.02)YC(+57.02)YGLPDSVK**	16,79	2526,065	22	4	843,0325	43,89	0	1	15065	150211-Fr_I_Helo-red-alk-tryp-001.raw	1	1	AAC25688.1:O77463.1:AAC25689.1	Carbamidomethylation	C12:Carbamidomethylation:1000.00;C14:Carbamidomethylation:1000.00
**GF(sub S)DVTGGFPVNSNNCIYPCYSTQDEIQC(+57.02)EEFC(+57.02)EK**	16,77	3949,622	34	-2,1	1317,545	45,43	0	1	15612	150211-Fr_I_Helo-red-alk-tryp-001.raw	1	1	JAW07006.1	Carbamidomethylation	C28:Carbamidomethylation:1000.00;C32:Carbamidomethylation:1000.00
**C(+57.02)VQ(sub A)SDGFIMGDSIGENK**	16,76	1855,808	17	-0,2	928,9111	31,18	0	1	10473	150211-Fr_I_Helo-red-alk-tryp-001.raw	1	1	AMO02558.1:JAW07025.1:JAW06984.1	Carbamidomethylation	C1:Carbamidomethylation:1000.00
**ER(+14.02)AEIDFSGIPEDIIK**	16,75	1844,952	16	2,2	923,4852	50,81	0	1	17461	150211-Fr_I_Helo-red-alk-tryp-001.raw	1	1	JAW06987.1:JAW07038.1:JAW06992.1	Methylation(KR)	R2:Methylation(KR):26.51
**KEDHYNVM(+15.99)TK**	16,71	1279,587	10	-1,3	640,7998	8,21	0	1	2329	150211-Fr_I_Helo-red-alk-tryp-001.raw	6	6		Oxidation (M)	M8:Oxidation (M):1000.00
**TW(sub Q)FGC(+57.02)PAYEGYC(+57.02)NDHC(+57.02)NDIER**	16,69	2663,026	21	1	888,6836	15,13	0	1	4826	150211-Fr_I_Helo-red-alk-tryp-001.raw	5	5	P0C2F3.1:P69940.2	Carbamidomethylation	C5:Carbamidomethylation:1000.00;C12:Carbamidomethylation:1000.00;C16:Carbamidomethylation:1000.00
**TE(sub Q)FGC(+57.02)PAYEGYC(+57.02)NDHC(+57.02)NDIER**	16,68	2605,99	21	5,2	869,675	18,24	0	1	5991	150211-Fr_I_Helo-red-alk-tryp-001.raw	4	4	P0C2F3.1:P69940.2	Carbamidomethylation	C5:Carbamidomethylation:1000.00;C12:Carbamidomethylation:1000.00;C16:Carbamidomethylation:1000.00
**T(+75.98)VAYNTAGISLGSGVC(+57.02)K**	16,61	1772,826	17	-0,3	887,42	36,38	0	1	12333	150211-Fr_I_Helo-red-alk-tryp-001.raw	1	1	AIJ02110.2:JAG85213.1	EDT; Carbamidomethylation	T1:EDT:0.00;C16:Carbamidomethylation:1000.00
**TC(+57.02)FLTAAGYC(+57.02)NTEC(+57.02)TLK(+43.01)K**	16,6	2179,97	18	-0,7	727,6634	34,92	0	1	11807	150211-Fr_I_Helo-red-alk-tryp-001.raw	1	1	AAC25688.1:O77463.1:AAC25689.1	Carbamidomethylation; Carbamylation	C2:Carbamidomethylation:1000.00;C10:Carbamidomethylation:1000.00;C14:Carbamidomethylation:1000.00;K17:Carbamylation:0.00
**ETRPISSWHTIGILM(+15.99)RWNKELQISYVFK**	16,57	3447,813	28	3,3	690,572	53,88	0	1	18472	150211-Fr_I_Helo-red-alk-tryp-001.raw	1	1	JAG85161.1	Oxidation (M)	M15:Oxidation (M):1000.00
**ISFTYEINWKPSSIK**	16,53	1811,946	15	-2,2	906,9781	63,6	0	1	21612	150211-Fr_I_Helo-red-alk-tryp-001.raw	1	1			
**AEIDFS(+58.03)GIPEDIIKEIK**	16,52	1974,044	17	-2,7	988,0263	48,56	0	1	16698	150211-Fr_I_Helo-red-alk-tryp-001.raw	1	1	P84190.1	2 3-dihydro-2 2-dimethyl-7-benzofuranol N-methyl carbamate	S6:2 3-dihydro-2 2-dimethyl-7-benzofuranol N-methyl carbamate:1000.00
**PAPEAAPEAAPE(+43.99)PAAAAP**	16,48	1670,779	18	-0,5	836,3962	32,33	0	1	10872	150211-Fr_I_Helo-red-alk-tryp-001.raw	1	1	JAW07022.1:P86821.1	Carboxylation (E)	E12:Carboxylation (E):19.86

**FIRPS(+380.15)GFC(+57.02)DGYC(+57.02)K**	16,35	1985,854	13	-2,3	662,957	38,67	0	1	13180	150211-Fr_I_Helo-red-alk-tryp-001.raw	1	1	C0HJM9.1:JAG85185.1:CEF39517.1:P60276.1	Nucleophilic addition to cytopiloyne+H2O; Carbamidomethylation	S5:Nucleophilic addition to cytopiloyne+H2O:8.26;C8:Carbamidomethylation:1000.00;C12:Carbamidomethylation:1000.00
**KVTCFLTAAGYC(+57.02)NTEC(+57.02)T(sub K)LQ**	16,34	2178,975	19	-0,6	727,3318	36,64	4,00E+09	1	12429	150211-Fr_I_Helo-red-alk-tryp-001.raw	1	1	AAW72463.1:Q5G8A8.1	Carbamidomethylation	C12:Carbamidomethylation:1000.00;C16:Carbamidomethylation:1000.00
**ITC(+57.02)FLTAAGYC(+57.02)NTEC(+57.02)T(+59.02)LKK**	16,31	2309,068	19	0,6	1155,542	18,47	0	1	6077	150211-Fr_I_Helo-red-alk-tryp-001.raw	1	1	AAC25688.1:O77463.1:AAC25689.1	Carbamidomethylation; Aminoethylcysteine	C3:Carbamidomethylation:1000.00;C11:Carbamidomethylation:1000.00;C15:Carbamidomethylation:1000.00;T16:Aminoethylcysteine:0.00
**IYEYSR**	16,25	829,397	6	0	415,7058	4,02	0	1	959	150211-Fr_I_Helo-red-alk-tryp-001.raw	1	1			
**DYLLYC(+57.02)DFS(+58.03)DKDPK**	16,21	1835,816	14	-2,1	612,9447	34,82	0	1	11773	150211-Fr_I_Helo-red-alk-tryp-001.raw	2	2	JAW07031.1:JAW06995.1:JAG85161.1	Carbamidomethylation; 2 3-dihydro-2 2-dimethyl-7-benzofuranol N-methyl carbamate	C6:Carbamidomethylation:1000.00;S9:2 3-dihydro-2 2-dimethyl-7-benzofuranol N-methyl carbamate:1000.00
**QPEAAAGVLEYAMK**	16,2	1476,728	14	-0,5	739,371	29,72	0	1	9983	150211-Fr_I_Helo-red-alk-tryp-001.raw	1	1			
**TTMDGQLIK**	16,18	1005,517	9	-0,3	503,7654	6,09	0	1	1559	150211-Fr_I_Helo-red-alk-tryp-001.raw	1	1			
**AEIDFSGIPE(+37.95)D**	16,18	1229,476	11	2,2	615,7468	33,36	0	1	11244	150211-Fr_I_Helo-red-alk-tryp-001.raw	1	1	JAW06987.1:JAW07038.1:JAW06992.1:P84190.1	Replacement of 2 protons by calcium	E10:Replacement of 2 protons by calcium:0.00
**E(+42.01)ALAPEPAPK(+14.02)PAPEPAPEPAPEP**	16,18	2357,179	23	2,3	786,7354	36,05	0	1	12213	150211-Fr_I_Helo-red-alk-tryp-001.raw	1	1	P0C8W6.1	Acetylation (Protein N-term); Methylation(KR)	E1:Acetylation (Protein N-term):1000.00;K10:Methylation(KR):1000.00
**LIINVNK**	16,17	812,512	7	0	407,2633	29,18	0	1	9807	150211-Fr_I_Helo-red-alk-tryp-001.raw	3	3			
**AVGITSM(+15.99)SPEDILK**	16,17	1475,754	14	-1,2	738,8835	43,56	0	1	14948	150211-Fr_I_Helo-red-alk-tryp-001.raw	1	1		Oxidation (M)	M7:Oxidation (M):1000.00
**APEPAPEPAPEAAPEP**	16,17	1568,736	16	-3,1	785,3727	13,97	0	1	4401	150211-Fr_I_Helo-red-alk-tryp-001.raw	1	1	P0C8W6.1		
**AEIDFSGIPEDIIK(+14.02)EIK**	16,17	1930,03	17	-0,5	644,3502	48,48	0	1	16671	150211-Fr_I_Helo-red-alk-tryp-001.raw	1	1	P84190.1	Methylation(KR)	K14:Methylation(KR):9.42
**PEAAPEPAAAAPE(+43.04)AAPE**	16,14	1630,784	17	-1,8	816,3977	32,64	0	1	10984	150211-Fr_I_Helo-red-alk-tryp-001.raw	1	1	P0C8W6.1	Carboxyl modification with ethanolamine	E13:Carboxyl modification with ethanolamine:14.77
**TVKFRALNEEIELK**	16,13	1688,946	14	-1,8	563,9883	35,69	0	1	12085	150211-Fr_I_Helo-red-alk-tryp-001.raw	1	1			
**LLNSFDEM(+15.99)SEKLK**	16,05	1568,776	13	-1,6	785,3938	13,22	0	1	4131	150211-Fr_I_Helo-red-alk-tryp-001.raw	1	1		Oxidation (M)	M8:Oxidation (M):1000.00
**EPAPEPAPEPAPE(+541.06)AAPEAAPE**	16,03	2607,009	21	-0,5	870,0098	34,78	0	1	11760	150211-Fr_I_Helo-red-alk-tryp-001.raw	1	1	JAW07022.1:P86821.1	ADP Ribose addition	E13:ADP Ribose addition:0.00
**PEAAPEAAPEPAAAAPER(-42.02)**	16,02	1701,821	18	-1,3	851,9166	32,61	0	1	10974	150211-Fr_I_Helo-red-alk-tryp-001.raw	1	1	JAW07022.1	Ornithine from Arginine	R18:Ornithine from Arginine:1000.00

**PEPAAAAPER(-43.05)R**	15,97	1120,551	11	-2	561,2818	11,94	0	1	3671	150211-Fr_I_Helo-red-alk-tryp-001.raw	1	1	JAW07022.1	Arginine oxidation to glutamic semialdehyde	R10:Arginine oxidation to glutamic semialdehyde:0.00
**LSMNAIDMIEDLLQK**	15,84	1732,874	15	2,1	578,6331	40,61	0	1	13882	150211-Fr_I_Helo-red-alk-tryp-001.raw	1	1			
**ILINNVK**	15,77	812,512	7	-0,3	407,2631	28,99	0	1	9744	150211-Fr_I_Helo-red-alk-tryp-001.raw	5	5			
**LLLNNVK**	15,77	812,512	7	-0,3	407,2631	28,99	0	1	9744	150211-Fr_I_Helo-red-alk-tryp-001.raw	5	5			
**AVNFGGSSK**	15,77	865,4293	9	0	433,722	26,61	0	1	8974	150211-Fr_I_Helo-red-alk-tryp-001.raw	2	2			
**T(+225.09)FC(+57.02)TPSTC(+57.02)K**	15,75	1325,557	9	-0,5	663,7853	13,97	0	1	4399	150211-Fr_I_Helo-red-alk-tryp-001.raw	1	1	JAW07031.1:JAW06995.1:JAG85161.1	Iminobiotinylation; Carbamidomethylation	T1:Iminobiotinylation:58.11;C3:Carbamidomethylation:1000.00;C8:Carbamidomethylation:1000.00
**SVVNNLQYK**	15,71	1063,566	9	-0,7	532,79	24,7	0	1	8356	150211-Fr_I_Helo-red-alk-tryp-001.raw	1	1			
**PEAAPEPAAAAPERR(-.98)**	15,68	1530,79	15	-5	766,3986	41,47	0	1	14195	150211-Fr_I_Helo-red-alk-tryp-001.raw	1	1	JAW07022.1	Amidation	R15:Amidation:1000.00
**ADVDFTGIAD(+37.95)S**	15,67	1147,435	11	2,1	574,7258	32,97	0	1	11101	150211-Fr_I_Helo-red-alk-tryp-001.raw	1	1	JAW07002.1:P86824.1	Replacement of 2 protons by calcium	D10:Replacement of 2 protons by calcium:12.28
**PEPAAAAPER(+380.15)RR**	15,66	1699,853	12	-1,5	850,9324	21,36	0	1	7168	150211-Fr_I_Helo-red-alk-tryp-001.raw	1	1	JAW07022.1	Nucleophilic addition to cytopiloyne+H2O	R10:Nucleophilic addition to cytopiloyne+H2O:0.00
**C(sub T)QFGC(+57.02)PAYEGYC(+57.02)T(sub M)NHC(+57.02)QDIER**	15,63	2607,003	21	0,3	1304,509	15,22	0	1	4860	150211-Fr_I_Helo-red-alk-tryp-001.raw	1	1		Carbamidomethylation	C5:Carbamidomethylation:1000.00;C12:Carbamidomethylation:1000.00;C16:Carbamidomethylation:1000.00
**EPAPEPAPE(+356.19)PAPE**	15,62	1685,797	13	0	843,9058	14,51	0	1	4599	150211-Fr_I_Helo-red-alk-tryp-001.raw	1	1	JAW07022.1:P86821.1:P0C8W6.1	Biotin polyethyleneoxide amine	E9:Biotin polyethyleneoxide amine:16.76
**T(+601.21)GIADSIIKK**	15,6	1645,824	10	-3,4	549,6135	36,23	0	1	12279	150211-Fr_I_Helo-red-alk-tryp-001.raw	2	2	JAW07002.1	EDT-maleimide-PEO-biotin	T1:EDT-maleimide-PEO-biotin:61.30
**TVEPH(+616.18)ILK**	15,6	1551,721	8	-0,8	776,8673	45,97	0	1	15803	150211-Fr_I_Helo-red-alk-tryp-001.raw	1	1	JAW07042.1	Heme	H5:Heme:1000.00
**ITC(+57.02)FLTAAGYC(+57.02)N(+.98)TEC(+57.02)TLK**	15,56	2122,937	18	-1,1	708,6522	68,32	0	1	23130	150211-Fr_I_Helo-red-alk-tryp-001.raw	1	1	AAC25688.1:O77463.1:AAC25689.1	Carbamidomethylation; Deamidation (NQ)	C3:Carbamidomethylation:1000.00;C11:Carbamidomethylation:1000.00;N12:Deamidation (NQ):1000.00;C15:Carbamidomethylation:1000.00
**LNDELIPK**	15,56	940,5229	8	0,5	471,269	13,8	0	1	4339	150211-Fr_I_Helo-red-alk-tryp-001.raw	1	1			
**VGVQ(sub S)QDDS(sub T)DYNER**	15,49	1523,649	13	0,9	762,8324	5,85	0	1	1474	150211-Fr_I_Helo-red-alk-tryp-001.raw	1	1			
**DNLIPGPPAAFDPDVLISAMS(+58.03)K**	15,48	2325,18	22	-2,6	776,0652	47,52	0	1	16344	150211-Fr_I_Helo-red-alk-tryp-001.raw	1	1	AIJ02109.2:P86392.3:V9Z9A3.1:AHE40589.1:AHE40588.1	2 3-dihydro-2 2-dimethyl-7-benzofuranol N-methyl carbamate	S21:2 3-dihydro-2 2-dimethyl-7-benzofuranol N-methyl carbamate:0.00
**KEALAPEPAPKPAPE(+21.97)PAPEPAPEPAPEAAPEPA**	15,48	3284,609	33	-2,1	1643,308	37,24	0	1	12656	150211-Fr_I_Helo-red-alk-tryp-001.raw	1	1	P0C8W6.1	Replacement of 2 protons by magnesium	E15:Replacement of 2 protons by magnesium:0.00
**DEIKDNVK**	15,48	959,4923	8	0,1	480,7535	1,53	0	1	356	150211-Fr_I_Helo-red-alk-tryp-001.raw	1	1			

**LTSKSE(+129.04)YAC(+57.02)PVIDK**	15,45	1738,845	14	-3,6	580,6201	30,3	0	1	10170	150211-Fr_I_Helo-red-alk-tryp-001.raw	1	1	Q0GY43.1:ABE98264.1	Monoglutamyl; Carbamidomethylation	E6:Monoglutamyl:1000.00;C9:Carbamidomethylation:1000.00
**SEYAC(+57.02)PAIDKFC(+57.02)EDHC(+57.02)AAK(+175.04)K**	15,39	2574,077	20	-3,6	859,0297	33,7	0	1	11368	150211-Fr_I_Helo-red-alk-tryp-001.raw	1	1	P86822.2:P0C8W4.2:AFP87551.1:Q0GY42.1:ABE98265.1:JAG85223.1	Carbamidomethylation; Naphthalene-2 3-dicarboxaldehyde	C5:Carbamidomethylation:1000.00;C12:Carbamidomethylation:1000.00;C16:Carbamidomethylation:1000.00;K19:Naphthalene-2 3-dicarboxaldehyde:0.00
**APEAAPEPAAAAPER(+453.21)**	15,35	1899,923	15	2,9	950,9714	17,82	0	1	5830	150211-Fr_I_Helo-red-alk-tryp-001.raw	1	1	JAW07022.1	Puromycin	R15:Puromycin:1000.00
**NIYEGPYPTLLIADPELIK**	15,31	2158,156	19	1,7	1080,087	49,7	0	1	17089	150211-Fr_I_Helo-red-alk-tryp-001.raw	1	1			
**AEIDFSGIPEDIIKQIKET(+122.01)NAKPPARF**	15,3	3148,621	27	-3,8	1575,312	38,9	5,35E+08	1	13261	150211-Fr_I_Helo-red-alk-tryp-001.raw	1	1	JAW06987.1:JAW07038.1:JAW06992.1	O-Isopropylphosphorylation	T19:O-Isopropylphosphorylation:61.26
**AAPEPAPEAAPEPVAAAAPER**	15,27	2011,001	21	-3,5	1006,504	14,43	0	1	4568	150211-Fr_I_Helo-red-alk-tryp-001.raw	1	1	JAG85215.1		
**LTSESEYAC(+57.02)PAIDK(+114.04)FC(+57.02)EDHC(+57.02)AAK**	15,23	2815,189	23	3,8	939,4071	32,89	0	1	11073	150211-Fr_I_Helo-red-alk-tryp-001.raw	1	1	Q0GY42.1:ABE98265.1:JAG85223.1	Carbamidomethylation; Ubiquitin	C9:Carbamidomethylation:1000.00;K14:Ubiquitin:12.36;C16:Carbamidomethylation:1000.00;C20:Carbamidomethylation:1000.00
**PAPEPAPEAAPEAAPEPAAAAPERR(+132.02)R**	15,21	2750,341	26	3,2	917,7906	37,99	0	1	12932	150211-Fr_I_Helo-red-alk-tryp-001.raw	1	1	JAW07022.1	Hydroxyphenylglyoxal arginine	R25:Hydroxyphenylglyoxal arginine:0.00
