# Supplementary material for: Proteome of fraction from Tityus serrulatus venom reveals new enzymes and toxins
Source: J Venom Anim Toxins Incl Trop Dis. 2019 Apr 18;25:e148218. doi: 10.1590/1678-9199-JVATITD-1482-18 (PMC6483408; doi:10.1590/1678-9199-JVATITD-1482-18)
Supplement: Additional File 2: [file 1678-9199-jvatitd-25-e148218-s2.pdf]

Supplementary Material to "Proteome of fraction from *Tityus serrulatus* venom reveals new enzymes and toxins"

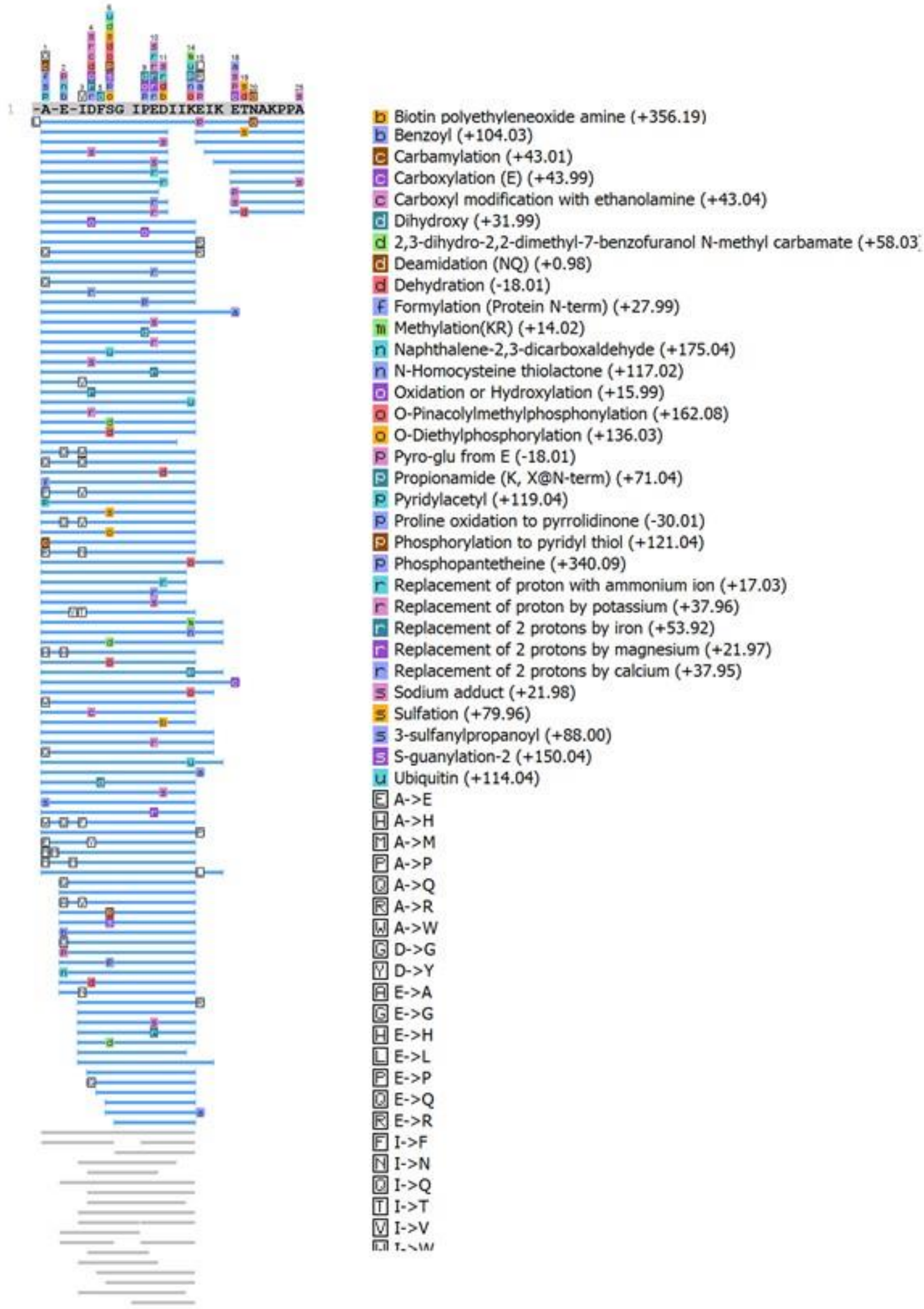

Additional file 2 - Hypotensin-2 (P84190.1) found in the Fraction I, in which the *de novo* sequencing showed 210 *de novo tags* resulted from at least 22 amino acid residues mutations.
